# Structural and Solution Speciation Studies on *fac*-Tricarbonylrhenium(I) Complexes of 2,2′-Bipyridine Analogues

**DOI:** 10.1021/acsomega.4c07117

**Published:** 2024-10-26

**Authors:** Tamás Pivarcsik, Jakob Kljun, Sergio Clemente Rodriguez, David Cortéz Alcaraz, Uroš Rapuš, Márta Nové, Egon F Várkonyi, József Nyári, Anita Bogdanov, Gabriella Spengler, Iztok Turel, Éva A. Enyedy

**Affiliations:** † MTA-SZTE Lendület Functional Metal Complexes Research Group, University of Szeged, Dóm tér 7-8., H-6720 Szeged, Hungary; ‡ Department of Molecular and Analytical Chemistry, Interdisciplinary Excellence Centre, University of Szeged, Dóm tér 7-8., H-6720 Szeged, Hungary; § Faculty of Chemistry and Chemical Technology, University of Ljubljana, Večna pot 113, SI-1000 Ljubljana, Slovenia; ∥ Universidad de Alcalá, 28805 Alcalá de Henares, Madrid, Spain; ⊥ Department of Medical Microbiology, Albert Szent-Györgyi Health Center and Albert Szent-Györgyi Medical School, University of Szeged, Semmelweis u. 6, H-6725 Szeged, Hungary

## Abstract

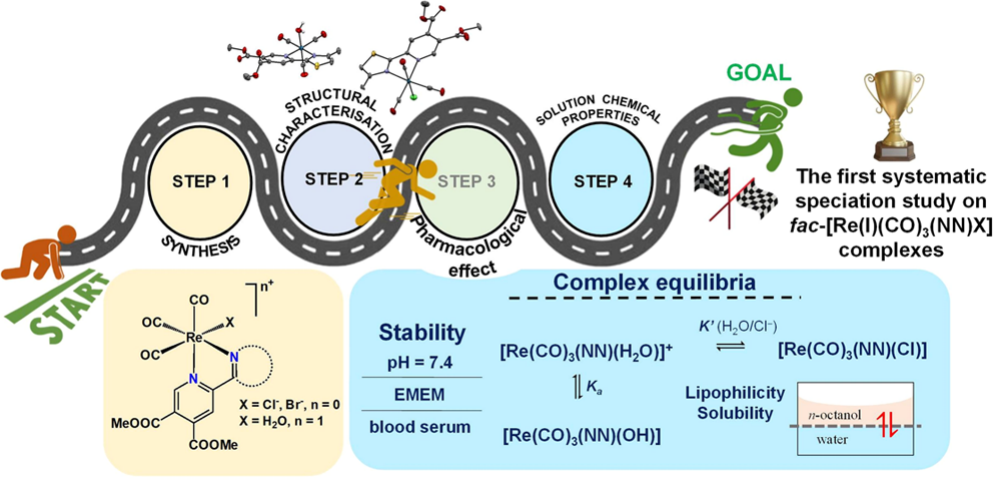

In this study, we report the synthesis and characterization of 12 novel rhenium(I) complexes with the general formula *fac*-[Re(CO)_3_(NN)X]^n+^ where (NN) is a 2,2′-bipyridine analogue ligand, X = Cl^–^, Br^–^, or H_2_O, and *n* = 0 or 1, focusing on their speciation in an aqueous solution. The prepared organorhenium complexes are stable in a wide pH range in an aqueous solution, and no release of the bidentate ligands or the carbonyl ligands was observed. The stability of the complexes in various biologically relevant matrices (cell culture medium and real blood serum) was also demonstrated. However, the simultaneous substitution of the halido ligand by water and slow hydrolysis of the ester bonds in the ligands were observed, affecting both the solubility and the lipophilicity of the compounds. The aqua complexes became more lipophilic in the presence of chloride ions, while the hydrophilicity increased significantly with time due to the hydrolysis of the ester bonds, which probably contributed to their weak pharmacological activity. The results also showed kinetically hindered aqueous solvation of the halido complexes and low chloride ion affinity of the aqua complexes. The deprotonation of the coordinated aqua ligand in the complexes occurs in the pH = 7–10 range, leading to significant formation (18–30%) of hydroxido species at pH = 7.4. The halido complexes showed somewhat higher cytotoxicity (IC_50_ = 60–99 μM) on human colon adenocarcinoma cancer cells (Colo205 and Colo320) than the corresponding aqua complexes (IC_50_ > 100 μM). In all cases, no antibacterial effect was observed (MIC > 100 μM), but some of the complexes showed moderate antiviral activity (IC_50_ ∼ 50 μM) on Herpes simplex virus 2.

## Introduction

Organorhenium(I) tricarbonyl complexes have gained considerable attention in the last decades due to their wide range of biological activity, including antitumor,^1−7^ antiviral,^8,9^ and antibacterial^10,11^ properties. These complexes have been reported to exert their pharmacological action via enzyme inhibition, phototoxicity, DNA damage, mitochondrial effects, or regulation of oxidative stress. Particular *fac*-Re(CO)_3_ complexes were reported to exhibit strong activity against SARS-CoV-2, and inhibition of the main protease of the virus appears to be a key factor in their mechanism of action.^8,9,12^ A series of Re(CO)_3_ complexes with the general formula *fac*-[Re(CO)_3_(NN)(azole)]^+^, where (NN) is a bidentate oligopyridine ligand and azole is a monodentate bioactive ligand (clotrimazole or ketoconazole), showed excellent activity against epimastigotes and trypomastigotes of *Trypanosoma cruzi*, the parasitic agent responsible for Chagas disease.^13^ In recent years, we developed metal complexes of different pyridine-4,5-dicarboxylate ester ligands with (NN) bidentate coordination mode and screened their biological properties. The various biological tests (antimicrobial activity, enzyme inhibition, interactions with various biological molecules) performed for organoruthenium(II), silver(I), copper(II), and zinc(II) complexes of these ligands revealed some promising results.^14−18^

**Chart 1:**
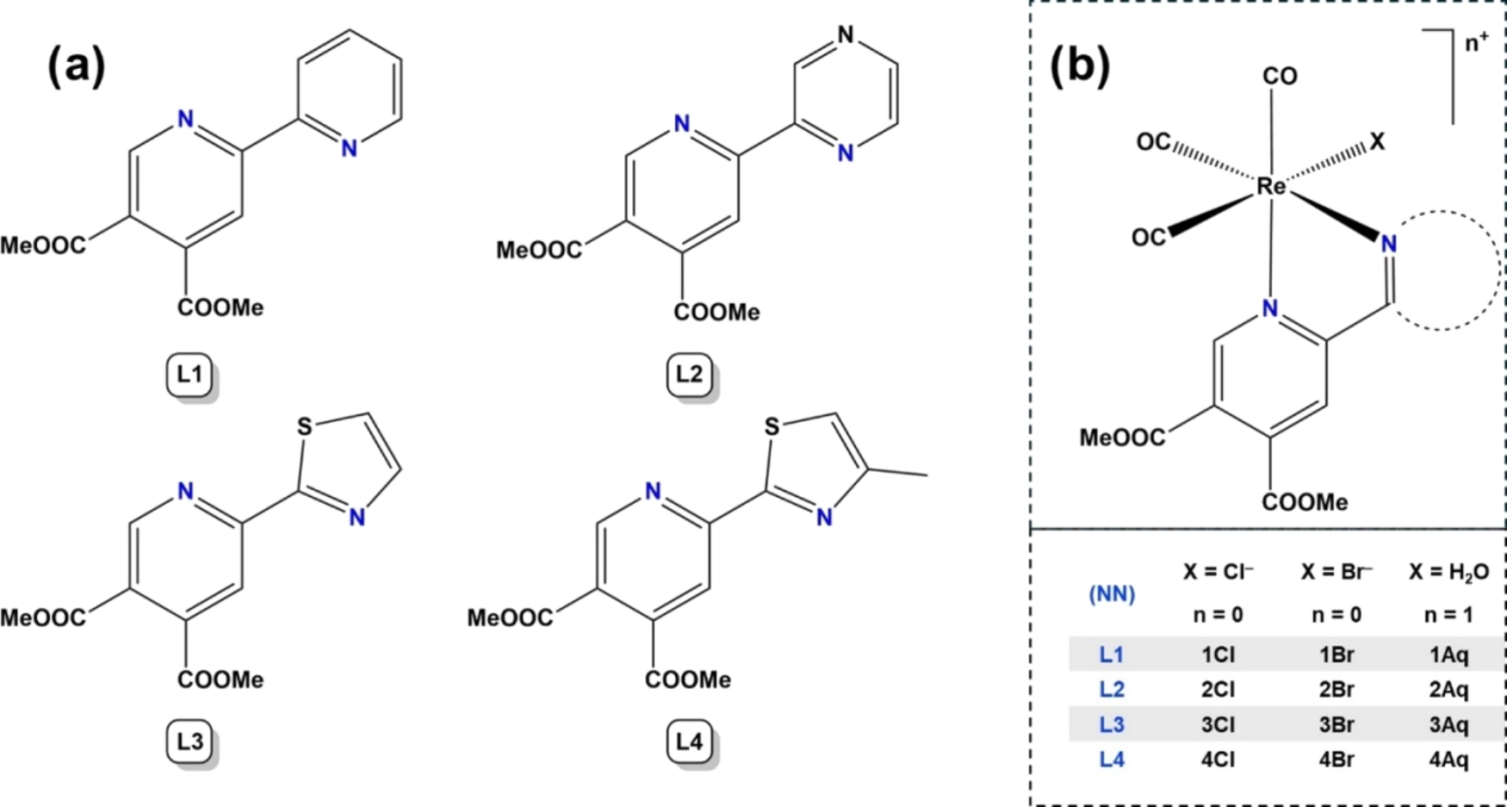
(a) Chemical Structures of the Pyridine-4,5-dicarboxylate Ligands **L1**–**L4** Used for the Synthesis of the (b) *fac*-[Re(CO)_3_] Complexes

Herein, we report the synthesis, structural characterization, and solution speciation studies of 12 new organorhenium(I) tricarbonyl complexes with 2,2′-bipyridine analogue pyridine-4,5-dicarboxylate ligands (Chart 1). Although numerous *fac*-[Re(CO)_3_] complexes have been developed in recent years, their behavior in aqueous solutions has been relatively unexplored. The novel compounds were tested on the Colo205 human colon adenocarcinoma cell line and its drug-resistant counterpart (Colo320). As multidrug resistance (MDR) is a serious drawback not only in chemotherapy but also in bacterial infections, the antibacterial activity of the compounds was monitored against various bacterial strains, including methicillin-resistant *Staphylococcus aureus* (MRSA), known to be responsible for difficult-to-treat hospital-acquired infections.^19^

In addition to drug resistance, the lack of selectivity toward cancer cells over healthy cells is a serious problem, leading to a significant weakening of the immune system during chemotherapy. Viral infections are also recognized as a potential threat to patients. Examples of compounds with dual or multiple therapeutic effects were also reported, demonstrating synergy between anticancer, antibacterial, and antiviral properties.^20,21^ Consequently, developing compounds with simultaneous pharmacological properties would obviously be beneficial.^22^ Considering these factors, the title compounds were evaluated not only for their anticancer and antibacterial activities but also for their antiviral effects.

## Results and Discussion

### Synthesis and Characterization of Compounds

**Scheme 1:**
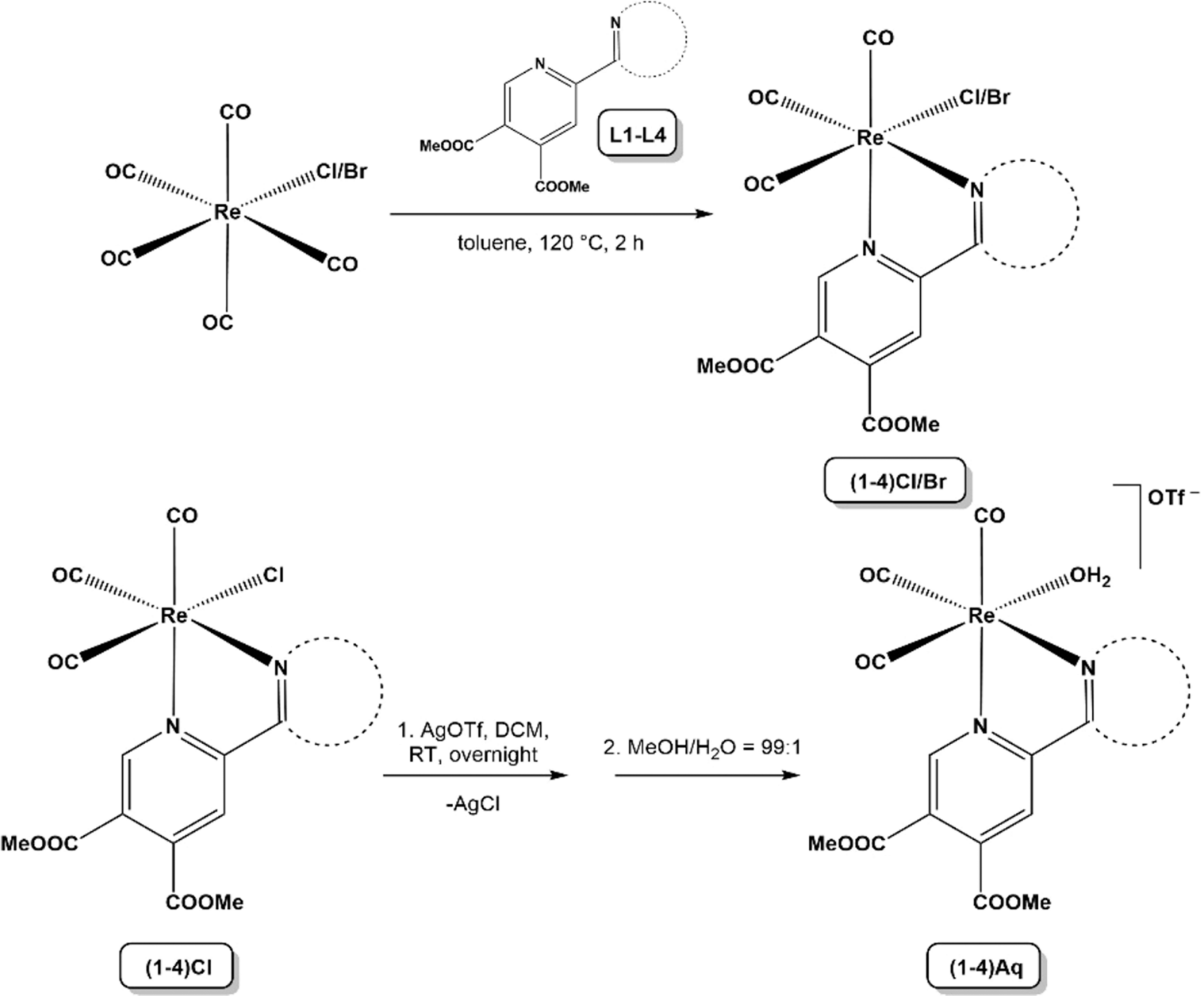
Preparation of The Halido and Aqua Complexes

Ligands **L1**–**4** (Chart 1a and S1) were synthesized according to the reported procedure.^14,23^ Organorhenium(I) *fac*-tricarbonyl chlorido, bromido, and aqua complexes **1**–**4Cl**, **1**–**4Br**, and **1**–**4Aq** (Chart 1b and S1) were newly prepared through one-step synthesis for chlorido and bromido or two-step procedure for aqua complexes (Scheme 1). Neutral halido complexes **1**–**4Cl** and **1**–**4Br** were prepared according to a previously reported procedure^24,25^ with some modifications. The reaction mixture was stirred in toluene for 2 h at 120 °C in a high-pressure tube. The precipitate formed was collected, and the red-to-orange solids were found to be light, air, and moisture stable.

The positively charged aqua complexes **1**–**4Aq** were prepared according to the literature procedure starting from **1**–**4Cl** and silver triflate in CH_2_Cl_2_.^26^ The reaction was carried out in the dark overnight at room temperature. Formed AgCl was removed by vacuum filtration over Celite. The solvent was evaporated, and the residue was dissolved in a mixture of methanol and water in a ratio of 99:1 and left to slowly evaporate. The product was obtained after 5 days when all the solvent evaporated.

All the prepared compounds were analyzed using ^1^H NMR spectroscopy (Figures S1–S12), mass spectrometry (MS), infrared (IR) and UV–visible (UV–vis) spectroscopy, and elemental analysis (CHN), and where we were able to obtain suitable crystals, X-ray structure analysis was also performed. The formation of the complexes such as *fac*-[Re(CO)_3_(L)Cl] (**1**–**4Cl**), *fac*-[Re(CO)_3_(L)Br] (**1**–**4Br**), and *fac*-[Re(CO)_3_(L)H_2_O]CF_3_SO_3_ (**1**–**4Aq**) (Chart 1b) is fully supported by ESI-MS and the spectroscopic experiments.

### Crystallization and X-Ray Structure Analysis

**Figure 1:**
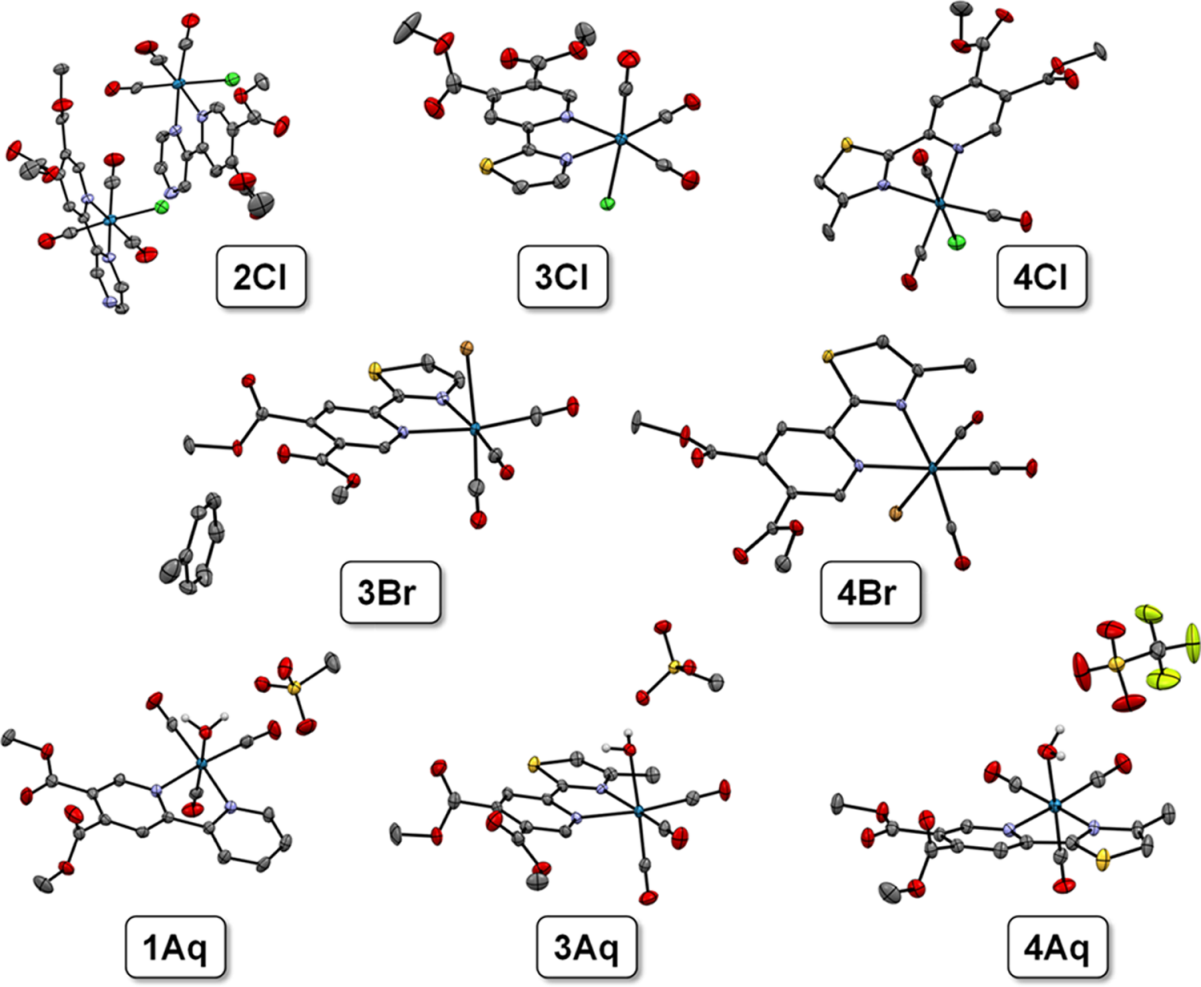
Crystal structures of rhenium(I) tricarbonyl complexes in the top row: **2Cl**, **3Cl**, and **4Cl**, in the middle row: **3Br** and **4Br**, and in the bottom row: **1Aq**, **3Aq** (both as methanesulfonate salts), and **4Aq** (as the triflate salt). Thermal ellipsoids are drawn at the 50% probability level. All hydrogens except H_2_O hydrogen atoms in aqua complexes are omitted.

Single crystals of **2Cl**, **3Br**, **4Br**, and **4Aq** (Figure 1) were prepared with different methods of crystallization. For the structures of **3Br** and **4Br** crystals were obtained from the mother liquor of toluene by slow evaporation. Similarly, single crystals of **4Aq** and **2Cl** were grown by slowly evaporating solvents from a solution of methanol and water and from a solution of acetone and water, respectively. Compounds **3Cl** and **4Cl** were successfully crystallized from the solution of chloroform with vapor diffusion of *n*-heptane utilizing the CrystalBreeder multireactor crystallization platform.^27^ Compounds **1Aq** and **3Aq** have been crystallized as methanesulfonate salts from a solution of methanol and water. Methanesulfonate complexes were prepared with the same procedure as triflate aqua complexes substituting silver triflate with silver methanesulfonate as a reagent and were used as crude products only for the crystallization experiments. A different silver salt was used to stabilize the structure with hydrogen bonding. The images of the analyzed single crystals are tabulated in Table S1, and the crystal structures of the eight organometallic compounds were determined by X-ray crystallography (Figure 1, Table S2–S4).

**Scheme 2:**
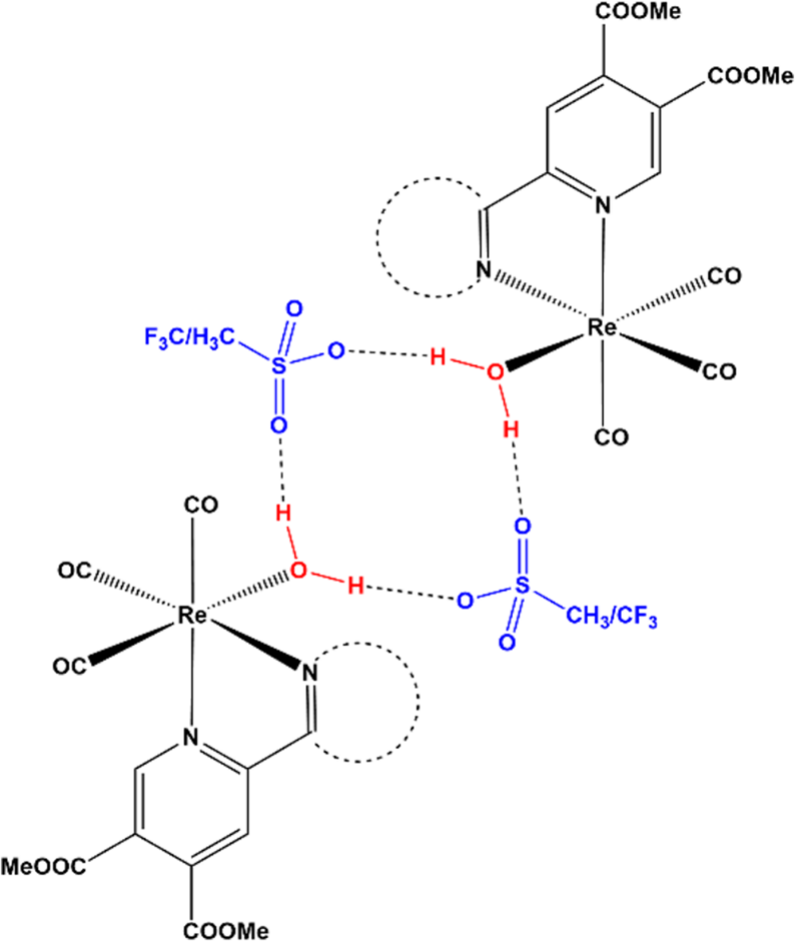
Supramolecular Tetrameric Hydrogen Bond Network Present in Rhenium Aqua Complexes **1Aq**, **3Aq**, and **4Aq**

All compounds present a slightly distorted octahedral geometry mainly attributed to the constrained geometry and relatively low bite angle (ca. 75°) of the chelating ligand coordinated via the (NN) donor set. The carbonyl ligands are expectedly bound in facial geometry. Bond lengths and angles are all within the expected range. In all cases (except **3Aq** where bond lengths are equal), the Re–N bond lengths show a slightly longer bond (0.01–0.03 Å) between the pyridine bearing two-electron acceptor ester groups in comparison to the second (unsubstituted) heterocycle (Table S5). The only other observable trend in bond length changes where in the reported structures we can observe a gradual increase in the Re–C bond length and a decrease in the respective C–O carbonyl bond length of the carbonyl ligand opposite the halido or aqua ligand X (Table S5). This can be attributed to the increasing *trans* effect of the ligand X (Br^–^ > Cl^–^ > OH_2_). In the case of all aqua complexes, we can observe the formation of a hydrogen bond network between the aqua ligand and the oxygen atoms of the respective counterion (methanesulfonate or triflate) with the O–O distance being ca. 0.2 Å shorter for the more basic methanesulfonate ion. Each aqua ligand acts as a hydrogen bond donor to two separate sulfonate groups, which in turn act as bridging hydrogen bond acceptors to the neighboring rhenium species. The tetrameric supramolecular arrangement is illustrated in Scheme 2.

### Solution Chemical Properties of the Halido Complexes

We first chose the halido derivatives to study their solution chemical properties in detail, since their behavior in aqueous solutions might be more complex, due to the presence of coordinated chloride coligand, compared to aqua species. When the halido complexes are dissolved in water, several transformation processes can take place. The coordinated halido coligand may be replaced by water, then the aqua ligand may deprotonate, the (NN) bidentate ligand may dissociate, and the ester bonds in the ligand scaffold may be hydrolyzed. These processes are obviously dependent on the conditions (e.g., solvent, pH, temperature) and do not necessarily occur instantaneously. The stability of a series of antiparasitic Re(I) tricarbonyl complexes of chelators with (NN) donor set such as bpy, 1,10-phenantroline, and their derivatives was investigated in different media (e.g., DMSO, various DMSO/blood serum mixtures).^13^ These compounds showed high stability in the biologically relevant matrices tested without releasing the bidentate ligand. However, there is much less known about the possible coligand exchange processes in such types of complexes studied in this work, in particular the exchange of the chlorido/bromido (or other monodentate) coligand with water and its subsequent deprotonation.

As a first step, the solubility of the halido complexes (**1**–**4Cl**, **3Br**) was studied in an aqueous solution. Based on previous solubility tests, the aqua analogues were completely soluble in 20% (v/v) DMSO/H_2_O medium at a concentration of 5 mM, whereas the halido complexes were practically insoluble and were soluble only in DMSO at the same concentration. This finding suggests that replacing the chlorido coligand with water increases the solubility of the complexes, since halido complexes are neutral compounds, while aqua derivatives are positively charged with a chloride counterion. However, for aqua complexes, the use of triflate as a counteranion is also beneficial for the water solubility. It was observed that the dissolution/solvation of the halido complexes in water at pH 7.4 and at 0.1 M KCl ionic strength is rather slow (>24 h needed to reach equilibrium); thus, the solubility was determined after a waiting time of 24 h (Figure S13), revealing the low aqueous solubility of halido complexes (<160 μM). The solubility was significantly decreased due to the additional methyl group on the ligand scaffold (**3Cl** vs **4Cl**). Complexes **3Cl** and **3Br** showed the highest solubility and were therefore selected for further investigation, while **3Aq** and **1Cl** were also included for comparison in certain cases.

**Figure 2:**
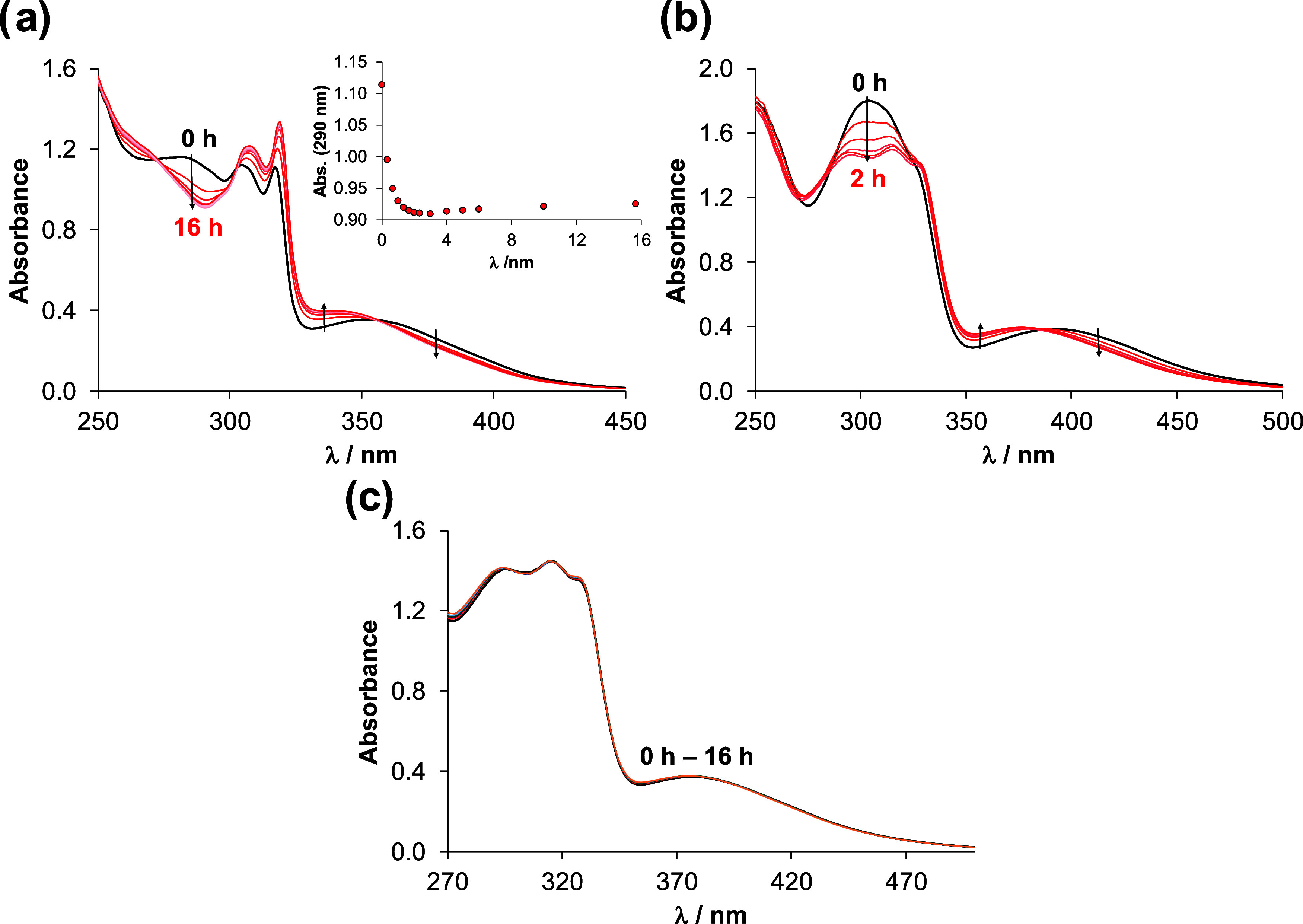
UV–vis spectra of (a) *fac*-[Re(CO)_3_(bpy)(Cl)] chlorido complex, (b) chlorido complex **1Cl** and (c) aqua complex **1Aq** in 5% (v/v) DMSO/H_2_O followed over time. Inserted figure in (a) shows absorbance values at 290 nm (●) plotted against time. {*c*_complex_ = 50 μM; pH ∼ 6.2;  = 1 cm; *T* = 25.0 °C}.

Initially, the changes in the UV–vis spectrum of complexes **3Cl** and **3Br** dissolved in water (at pH = 6.1) at low concentration (55 μM) were followed over time, and no significant spectral changes were observed over ca. 58.5 h (Figure S14). Interestingly, the normalized spectra of both complexes are identical to that of **3Aq** (Figure S15), suggesting that the compounds have the same composition under the conditions used; they are most likely in the aqua complex form. This suggestion was further confirmed by ^1^H NMR spectroscopy, and the same peaks were found for all three complexes (Figure S16). However, minor peaks appeared after a waiting time of 24 h, most probably due to the partial hydrolysis of the ester bond in the bidentate ligand (vide infra), which did not affect the charge transfer bands in the UV–vis spectra. In order to follow the time dependence of the exchange of the coordinated chlorido coligand to H_2_O, complexes **1Cl** and **1Aq** were dissolved in DMSO. In this solvent, their spectra significantly differ due to the coordination of the different coligands and remained intact up to 16 h (Figure S17). Nevertheless, partial coordination of DMSO to the metal ion is possible, since ^1^H NMR spectra of **3Cl** recorded in DMSO-*d*_6_ and acetonitrile-*d*_3_ significantly differ (shifts in peaks, not shown), and in this case, the solvent effect must be taken into consideration, which can be significant. The DMSO stock solutions were then diluted with water up to 5% (v/v) DMSO/H_2_O. The changes in the charge transfer bands were followed over time, and for comparison, *fac*-[Re(CO)_3_(bpy)(Cl)] was also studied (Figure 2). The spectrum of the chlorido complexes was changed over 2 h in 5% (v/v) DMSO/H_2_O, while **1Aq** remained intact over the monitored time (16 h). Moreover, the spectrum of **1Cl** after 2 h is very similar to that of **1Aq**, and both findings confirm our suggestion on the exchange process of the chlorido coligand to H_2_O (Figures 2 and S17). Based on the ^1^H NMR spectra recorded for **1Aq** and **1Cl** (Figure S18), the two sets of peaks appeared for **1Cl** after 5 min, indicating the copresence of aqua and chlorido complexes, and as it was expected the peaks belonging to the chlorido complex disappeared with time. This exchange process is not instantaneous. Therefore, the solvation of the halido complexes is kinetically hindered. To obtain information on the behavior of the chlorido complexes in the cell culture medium, the stock solution of **3Cl** (in DMSO-*d*_6_) was diluted with Eagle’s Minimum Essential Medium (EMEM) to 10% (v/v) DMSO-*d*_6_ and the ^1^H NMR spectra were recorded over time (Figure S19). For comparison, the spectra for **3Aq** were also recorded in the same way. Although the hydrolysis of ester bonds (vide infra) and the coordination of EMEM component(s) are clearly seen in parallel in both cases (and the replacement of Cl^–^ with H_2_O in the case of **3Cl** after 25 min), the ^1^H NMR spectra of **3Cl** and **3Aq** are almost identical in EMEM after 72 h.

### Solution Chemical Properties of The Aqua Complexes

**Figure 3:**
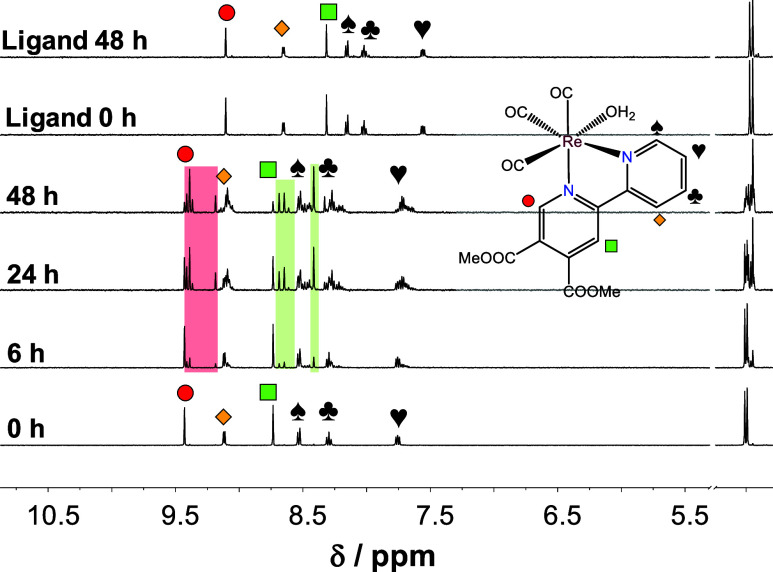
^1^H NMR spectrum of **1Aq** in the low-field region in phosphate buffer (20 mM, pH = 7.4) followed over time and that of the corresponding ligand is also shown. Rectangles with colored background show the appearance of a new set of peaks. {*c*_complex_ = 500 μM; 10% (v/v) D_2_O/H_2_O; *T* = 25.0 °C}.

As the aqua complexes are readily soluble in water, further stability measurements were carried out on them. The UV–vis spectrum of aqua complexes **1**–**4Aq** in water (pH ∼ 6.2) was followed over time. No significant spectral changes were observed during 24 h in the case of **1Aq** and **2Aq**, and after 36 h the spectra slightly changed (Figure S20), while for **3Aq** and **4Aq**, the spectra remain intact for 48 h (Figure S21). Additionally, ^1^H NMR spectra were recorded for **1Aq** (Figure S22), revealing the appearance of a new set of peaks after 24 h. The ratio of this new product is about 4% after 48 h, calculated from the integration of the corresponding peaks of the substituted pyridine ring protons. These changes indicate the hydrolysis of the ester bond of the coordinated ligand under the conditions used rather than the dissociation of the complex. The UV–vis spectra of **1**–**4Aq** were also recorded at pH 7.4 (in 20 mM phosphate buffer) showing significant changes over time. The reaction was completed within ∼36 h (Figure S23). The ^1^H NMR spectra also showed the decreasing signals belonging to the aqua complexes and the increasing signals of new peaks (Figure 3), which is suggested to be due to the coordination of the phosphate ions as coligand. Parallel to the coordination of the phosphate ion, slow hydrolysis of the ester bonds also occurred. Notably, for the ligand alone negligible (∼5%) hydrolysis could be observed after 48 h (Figure 3).

The effect of the HEPES buffer was also studied at pH 7.4. Both the UV–vis and ^1^H NMR spectra indicated changes during the monitored 48 h (Figure S24) although less new peaks appeared in the ^1^H NMR spectra compared to the presence of the phosphate ions. Analysis of the direct comparison of the effect of the two kinds of mediums (Figure S25) led to the conclusion that the two ester bonds in the coordinated ligand are hydrolyzed differently. The behavior of the aqua complexes **1**–**4Aq** was also studied in EMEM. The UV–vis spectra show a rather fast reaction (within 1 h), most probably, one of the components of the cell culture medium coordinates to the metal ion (Figure S26). It was also confirmed by ^1^H NMR spectroscopy, namely, a new set of peaks appeared in the spectrum (Figure S27). Interestingly, signals of hydrolyzed products were not identified most probably due to the slightly acidic conditions (pH ∼ 6.5). It should also be noted that precipitation occurred in the case of complexes **1Aq** and **4Aq** after 48 h, while complexes **2Aq** and **3Aq** remained dissolved (Figure S26). The spectral changes of the aqua complexes in blood serum were also monitored. The changes were found to be almost negligible for **1Aq** and **2Aq**, while they were more significant for **3Aq** and **4Aq**, but the charge transfer bands (∼360–500 nm) did not change much over time (Figure S28). ^1^H NMR spectrum of **2Aq** (Figure S29) in serum shows the appearance of new sets(s) of peaks; however, the Δδ between the original and the new chemical shift is small (∼0.06 ppm), which suggests the coordination of a serum component to the metal ion. In the case of complex **3Aq** (Figure S29b), a new peak set appeared further from the original peaks (Δδ = 0.24–0.36 ppm), which is related to the hydrolysis of the ester bond.

In summary, in the biological media used, there was no evidence for the release of the original bidentate and carbonyl ligands, while the labile aqua coligand underwent ligand exchange processes. On the other hand, the ester bonds of the ligand were hydrolyzed in a time-dependent manner.

The effect of the pH on the stability and ligand exchange processes of the complexes was studied using UV–vis spectrophotometry. No spectral changes were observed between pH 2 and 6 indicating that the complexes remained intact. However, the spectra showed a batochromic shift from pH ∼ 6 up to ∼9.5, and isosbestic points were also observed. (For a representative spectrum series for **1Aq** see Figure S30). Most probably this pH-dependent process is the deprotonation of the aqua ligand, and proton dissociation constants (p*K*_a_) could be calculated based on the spectral changes (Table 1).

**Table 1:** Proton Dissociation Constants of The Coordinated Water Co-Ligand (p*K*_a_ (H_2_O)) and Water/Chloride Ion Exchange Constants (log* K*′ (H_2_O/Cl^–^)) for the Aqua Complexes Determined by UV–vis and ^1^H NMR Spectroscopy^a^

		p*K*_a_ (H_2_O)		
method	complex	0.1 M KNO_3_	0.1 M KCl^b^	log* K*′ (H_2_O/Cl^–^)^a^
UV–vis	**1Aq**	8.07 ± 0.04	8.02 ± 0.08	0.80 ± 0.04
	**2Aq**	7.78 ± 0.04	7.78 ± 0.09	1.16 ± 0.11
	**3Aq**	7.99 ± 0.03	8.04 ± 0.04	0.74 ± 0.11
	**4Aq**	8.03 ± 0.02	n.d.	0.74 ± 0.05
^1^H NMR	**3Aq**	8.08 ± 0.02	n.d.	n.d.

a {*I* = 0.1 M KNO_3_ or KCl; *T* = 25.0 °C}.

b Equilibrium time: 1 h.

**Figure 4:**
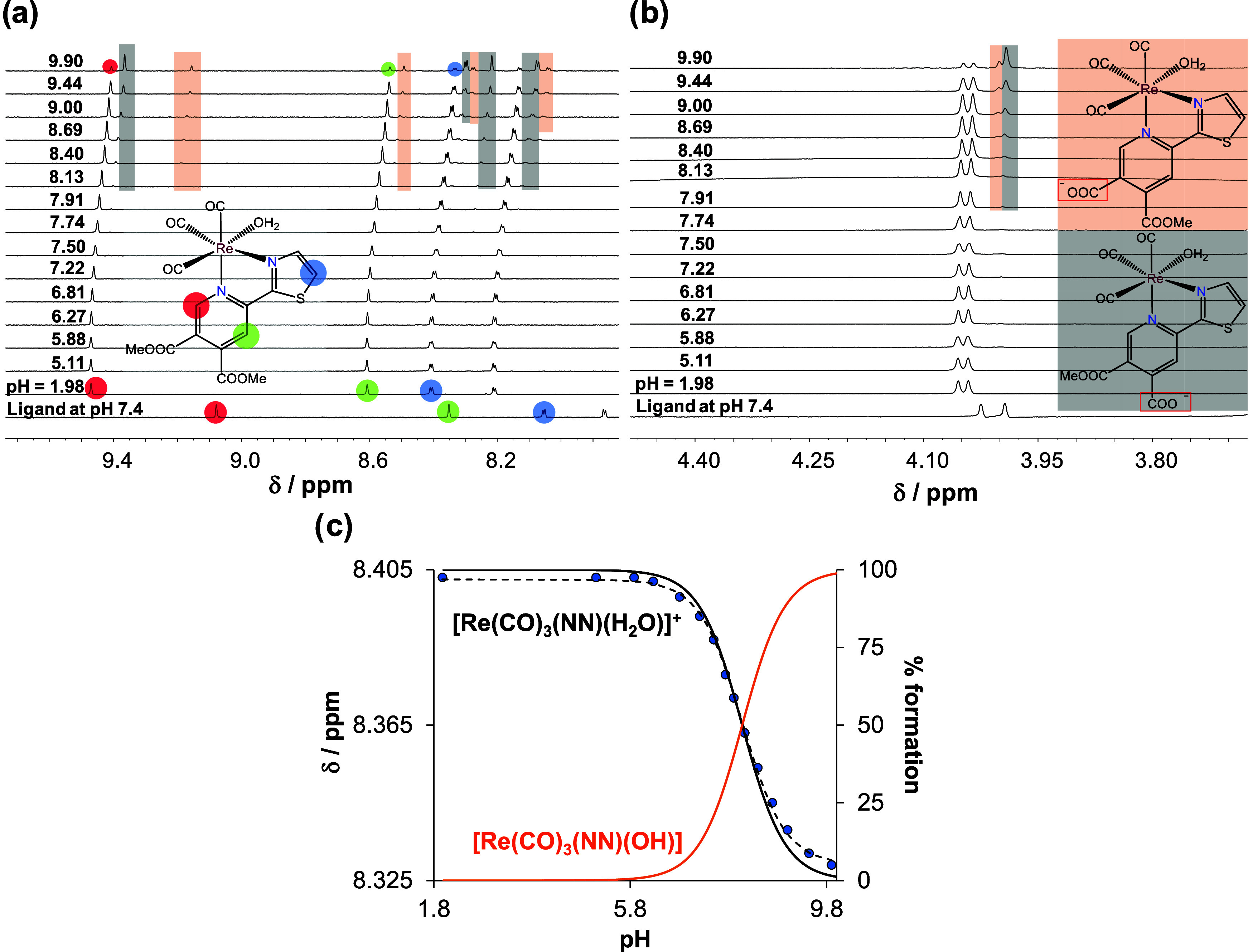
^1^H NMR spectra in the (a) low-field and (b) high-field region of **3Aq** complex at different pH values (1.98 → 9.90). The spectrum of the free ligand is also shown at pH = 7.4. Rectangles with colored backgrounds indicate the appearance of new sets of peaks related to hydrolyzed products. (c) Chemical shift values of C(5′)H proton (●) along with the fitted line and concentration distribution curves (black and orange solid lines) as a function of pH. It is noteworthy that traces of methanol were present in the solvent, but its peak intensity was increased in the basic pH range (not shown). {c_complex_ = 660 μM, *I* = 0.1 M KNO_3_, 10% (v/v) D_2_O/H_2_O, *T* = 25.0 °C}.

The p*K*_a_ values of the complexes are similar, and only for **2Aq** a somewhat lower value was obtained due to the negative inductive effect caused by the additional nitrogen in the heterocyclic ring. Notably, p*K*_a_ = 8.3 was reported for the analogous aqua complex of a 1,10-phenanthroline derivative.^28^^1^H NMR titration was also carried out for the complex **3Aq** (Figure 4), and the calculated p*K*_a_ (Table 1) is in good agreement with that obtained by the UV–vis titrations. The hydrolysis products (only two kinds) also appeared with increasing pH, and based on the integration of the corresponding peaks at pH 9.9, ∼80% of the **3Aq** complex is hydrolyzed and the ratio of the two hydrolysis products is 1:3. Based on the spectra recorded, the fully hydrolyzed form (when both functional groups are in their carboxylate form) could not be detected.

The p*K*_a_ values of the complexes (**1**–**3Aq**) were also determined at 0.1 M KCl ionic strength (Table 1) and similar values were obtained as in the absence of chloride ions, indicating a weak chloride ion affinity of the complexes. Moreover, UV–vis titrations were also performed for **3Cl** and **3Br**, and the determined p*K*_a_ values (7.98 ± 0.03 and 8.06 ± 0.03 for **3Cl** and **3Br**, respectively, in 0.1 M KNO_3_) are very similar to that of **3Aq**. These findings also confirm the formation of aqua complexes from halido complexes in water.

Based on the determined p*K*_a_ values, concentration distribution curves were calculated (for **1Aq** see Figure 4c) and the fractions of the aqua and mixed-hydroxido complexes are shown in Table S6 at pH 7.4. According to the calculations, the fraction of the mixed hydroxido complex is significant in all cases (18–30%).

It is noteworthy that deprotonation of the aqua ligand modifies the charge of the complex. However, ester hydrolysis also affects the overall charge. For **1Aq**, at pH 7.4 after 48 h (Figure S24b), only ∼12% of the complex remained in a nonhydrolyzed form (with COOMe substituents, +1 charge) and the rest (∼88%) are hydrolyzed products (with COOMe and COO^–^ substituents). Based on further calculations involving the p*K*_a_ (H_2_O) value, the amount of neutral complex (with COOMe and COO^–^ substituents, and H_2_O as coligand) is ∼72% (82% of the 88%), and the rest amount (16%; 18% of the 88%) of the complex is negatively charged (with COOMe and COO^–^ substituents, and OH^–^ as coligand).

**Figure 5:**
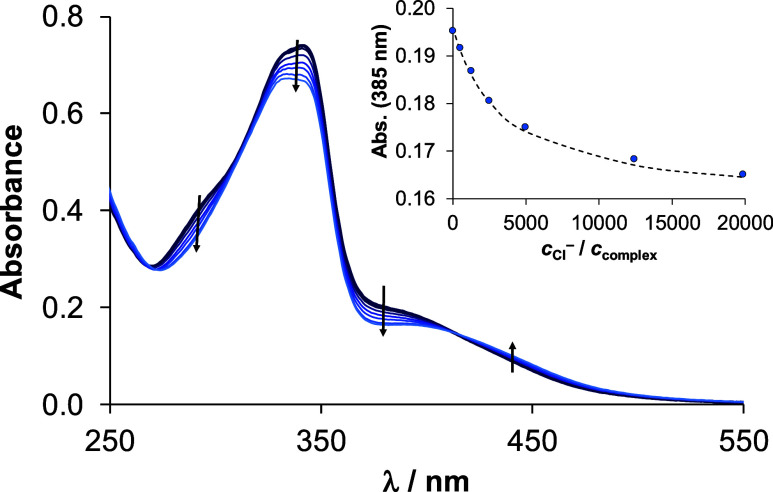
UV–vis spectra of aqua complex **3Aq** in the HEPES buffer (pH = 6.0) with increasing chloride ion concentration. Inserted figure shows absorbance values at 385 nm (●) plotted against *c*_Cl_^–^/*c*_complex_ along with the fitted (dashed) line. {*c*_complex_ = 40 μM *c*_Cl_^–^ = 0–800 mM;  = 1 cm; *T* = 25.0 °C}.

The exchange of the coordinated H_2_O with chlorido coligand was investigated by UV–vis and ^1^H NMR spectroscopy at pH 6.0. (This condition was chosen since, at this pH, based on the p*K*_a_ values (Table 1), the complexes are in their aqua form). Minor but tendentious changes in the UV–vis spectra were followed over time, and the equilibrium reached within 1 h (Figure S31). The ^1^H NMR spectra showed the appearance of a new set of peaks related to the formation of the chlorido complex (Figure S32a). Based on the integration of the corresponding peaks belonging to the aqua and chlorido forms, their molar ratio was plotted against time (Figure S32b), revealing a similar reaction time as seen in the case of the UV–vis studies (∼1 h). It is noteworthy that after 2 h precipitation occurred since the applied concentration (500 μM) is much higher compared to the UV–vis experiment (25 μM). Therefore, in order to characterize the chloride ion affinity of the aqua complexes, UV–vis spectra of individual samples containing the aqua complex at different chloride ion concentrations were recorded after a 1 h waiting time. A representative UV–vis spectrum series for complex **3Aq** is shown in Figure 5, and from the spectral changes relatively low exchange constants (log* K*′ (H_2_O/Cl^–^) ∼ 1) were computed (Table 1), indicating the weak chloride ion affinity of the aqua complexes.

### Lipophilicity of The Organorhenium Complexes

The lipophilicity of the complexes was characterized at pH 7.4. The traditional shake-flask method was applied in HEPES buffer (pH 7.4) to determine the distribution coefficient (*D*), via *n*-octanol/buffered aqueous solution partitioning. It is noteworthy, when the chloride ion is coordinated to Re(I), the actual charge changes from +1 to neutral, which probably has an impact on the lipophilicity; therefore, various chloride ion concentrations were applied, namely, *c*_Cl_^–^ = 4, 24, and 100 mM, which are relevant to different biofluids, such as nucleus, cytosol, and serum, respectively (Figure S33). The measurements were also conducted in the absence of chloride ions.

On the one hand, these complexes possess a lipophilic character (log *D*_7.4_ = +0.28 – +1.92); moreover, the additional methyl group present in complex **4Aq** increased the lipophilicity compared to the analogous **3Aq**. On the other hand, it is clearly seen that the higher the chloride ion concentration, the higher the lipophilicity of the complexes; however, the halido complexes were found to be more lipophilic compared to the corresponding aqua complexes even at *c*_Cl_^–^ = 100 mM, which is in line with the relatively low chloride ion affinity of the aqua complexes (Table 1). Notably, due to the low aqueous solubility of halido complexes, they were preliminarily dissolved in the *n*-octanol phase (Figure S33).

**Figure 6:**
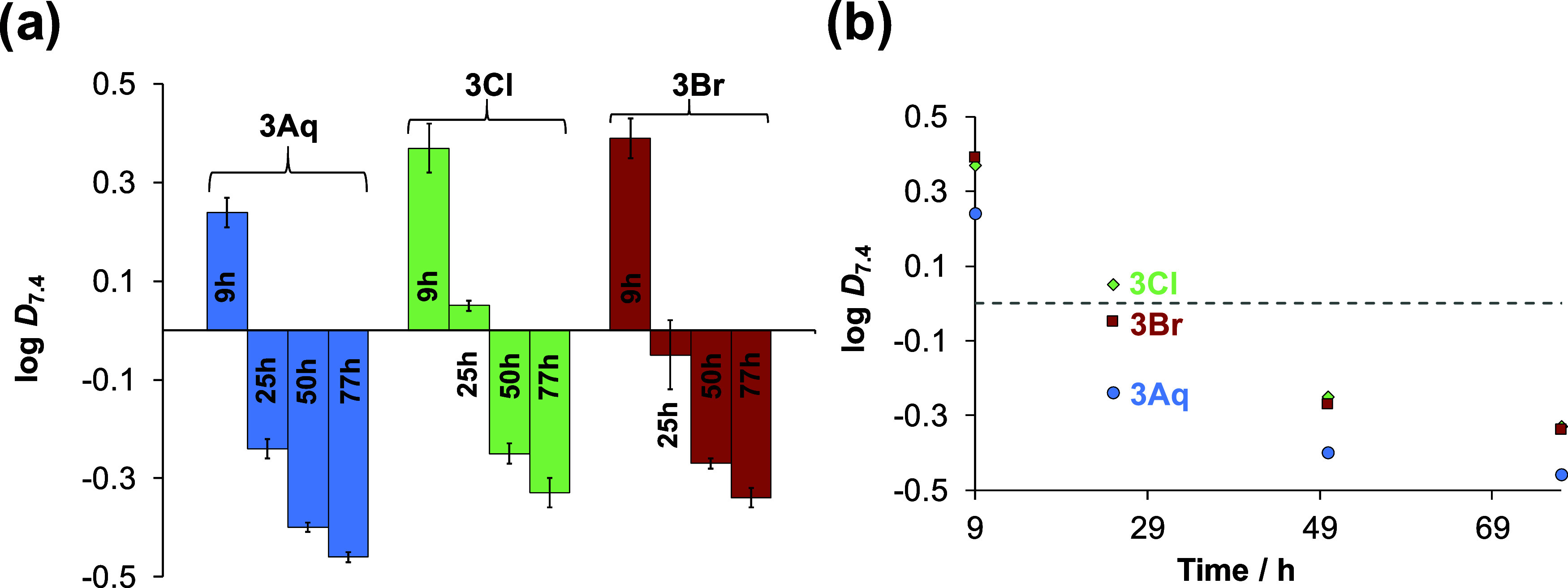
Lipophilicity (a) of selected complexes (**3Aq**, **3Cl**, and **3Br**) expressed as log* D*_7.4_ measured via *n*-octanol/buffered aqueous solution partitioning at pH = 7.4 (HEPES buffer) in the absence of chloride ions, investigated over time (9, 25, 50, and 77 h). For better visualization, all the log *D*_7.4_ values are plotted against time in (b). The complexes were previously dissolved in the water phase (buffered). {*c*_complex_ = 50 μM; *T* = 25.0 °C}.

Since **3Br** and **3Cl** have the highest solubility in the water among the halido complexes (Figure S13), the measurements for these compounds were also performed when they were dissolved in water. As a result, the determined log *D*_7.4_ values are very similar to those obtained in the case of the **3Aq** aqua complex. The measurements were conducted after 4 h waiting time, and within this time frame, the hydrolysis of the ester bonds in the complexes at pH = 7.4 is negligible. However, using a longer waiting time, it becomes more significant; therefore, the lipophilicity of **3Aq**, **3Br**, and **3Cl** was investigated also at various time points, namely, after 9, 25, 50, and 77 h in the absence of chloride ions, and the results are shown in Figure 6.

Over the 77 h time period, the lipophilicity of the three complexes significantly changes and becomes more hydrophilic (at 77 h, log *D*_7.4_ = −0.34 – −0.46). It is noteworthy that **3Aq** is the most hydrophilic compound at each time point compared to **3Cl** and **3Br**.

### Evaluation of The Pharmacological Activity of The Organorhenium Complexes

#### In Vitro Anticancer Activity of The Complexes on Human Colon Adenocarcinoma Cancer Cells

The cytotoxic activity of selected halido (**1**–**4Cl**, and **3Br** with chlorido and bromido coligand, respectively) and aqua (**1**–**4Aq**) complexes was evaluated in the drug-sensitive Colo205 and its resistant counterpart Colo320 human colonic adenocarcinoma cell lines using the colorimetric 3-(4,5-dimethylthiazol-2-yl)-2,5-diphenyl-tetrazolium bromide (MTT) assay. For comparison, *fac*-[Re(CO)_3_(bpy)(Cl)] (in which bpy denotes 2,2′-bipyridine) was also tested, and doxorubicin was used as a positive control. IC_50_ values for the halido complexes using 72 h incubation time along with their resistance factors (RF = IC_50_ (Colo320)/IC_50_ (Colo205)) are shown in Table 2. Based on these data, it can be concluded that the halido complexes (**1**–**4Cl** and **3Br**) showed weak cytotoxic activity (IC_50_ = 60–99 μM) against both drug-sensitive and -resistant cancer cells. The resistance factors of these complexes fall in the range of 1.03–1.25, which are significantly lower than that of doxorubicin. The aqua complexes (**1**–**4Aq**) were characterized by even higher IC_50_ values (>100 μM). The somewhat higher cytotoxic activity of the halido complexes might be the result of their kinetic inertness compared to aqua derivatives, based on the previous solution chemical studies.

**Table 2:** In Vitro Cytotoxicity of The Halido Complexes (**1–4Cl** and **3Br**), and *fac*-[Re(CO)_3_(bpy)(Cl)] as a Reference Compound, Tested in The Drug-Sensitive Colo205 and Its Resistant Counterpart Colo320 Human Colon Adenocarcinoma Cell Lines, In Addition to The Resistance Factors (RF = IC_50_(Colo320)/ IC_50_(Colo205))^a^

IC_50_ (μM)	Colo205	Colo320	RF
**1Cl**	87.24 ± 0.92	97.1 ± 1.6	1.11
**2Cl**	96.48 ± 0.10	99.16 ± 0.25	1.03
**3Cl**	66.0 ± 1.7	83.03 ± 0.50	1.26
**3Br**	62.6 ± 3.7	72.66 ± 0.40	1.16
**4Cl**	60.2 ± 1.3	76.3 ± 2.2	1.27
*fac*-[Re(CO)_3_(bpy)(Cl)]	25.2 ± 1.1	49.1 ± 1.7	1.95
doxorubicin	0.58 ± 0.06	3.13 ± 0.29	5.17

a IC_50_ values of **1–4Aq** > 100 μM in both cell lines. {incubation time: 72 h}.

On the one hand, the exchange of the pyridine (**1Cl**) heterocyclic ring to pyrazine (**2Cl**) has no considerable effect on the anticancer activity, while the presence of thiazole (**3Cl**, **4Cl**) increased the cytotoxicity, as IC_50_ values were decreased by 24 ± 3% in the case of the Colo205 cell line. On the other hand, the exchange of the chlorido for the bromido coligand (**3Cl** vs **3Br**) as well as the presence of an additional methyl group on the thiazole ligand scaffold (**3Cl** vs **4Cl**) had only a minor effect on the cytotoxicity. The IC_50_ values of the reference complex *fac*-[Re(CO)_3_(bpy)(Cl)] and its **1Cl** analogue show some differences, and despite the somewhat higher IC_50_ values of **1Cl**, it shows a lower resistance factor as well as the other 2,2′-bipyridine analogues.

#### Antibacterial Activity of The Complexes

The antibacterial activity of the selected complexes was tested in gram-negative *Escherichia coli* and gram-positive *S. aureus* as well as its methicillin-resistant counterpart (MRSA). The compounds showed no activity against the tested bacterial strains, as the minimum inhibitory concentration (MIC) was above 100 μM in each case. It is worth mentioning that silver complexes with the ligands used in this study showed remarkable activity against the standard panel of microorganisms (bacteria, fungi) and a selection of clinical isolates from the milk of the cow diagnosed with mastitis.^16^ Antifungal and especially antibacterial activities of corresponding copper(II) and zinc(II) complexes were much lower.^17,18^ Obviously, the type of metal ion bonded to these ligands is very important for biological activity.

#### Antiviral Activity of The Complexes

Selected compounds (**1**–**4Aq**, **1**–**4Cl**, and **3Br**) and the reference complex *fac*-[Re(CO)_3_(bpy)(Cl)] were tested for their antiviral activity against Herpes simplex virus 2 (HSV-2), which is a double-stranded DNA virus that infects mammals, including humans.^29^ The antiviral activity of complexes against HSV-2 was investigated by using Vero cells (originally isolated from kidney epithelial cells of an African green monkey) to host the growing viruses. First, the cytotoxicity of the complexes against Vero cells was assessed by an MTT assay (Figure S34), and it was found that the complexes display no significant activity at the applied concentrations. The maximum nontoxic concentration was found to be 50 μM for complex **1Aq**, 100 μM for the others, and 25 μM for *fac*-[Re(CO)_3_(bpy)(Cl)].

**Figure 7:**
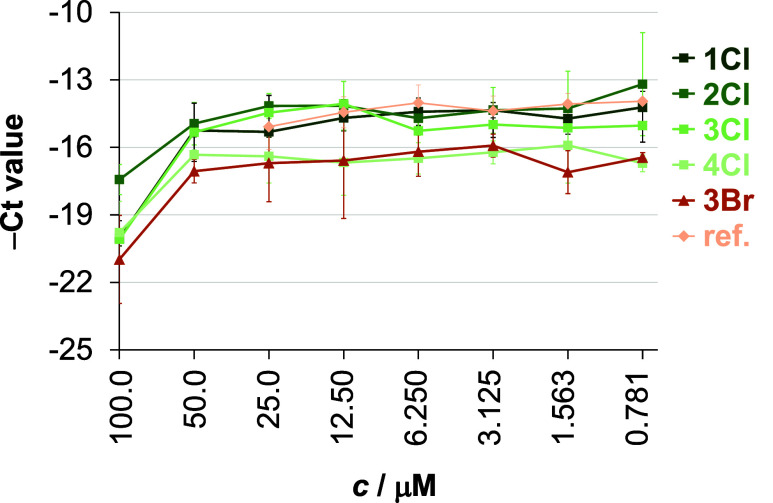
Antiviral effect of complexes **1–4Cl** and **3Br** against HSV-2 at different concentrations. As a comparison, the reference complex *fac*-[Re(CO)_3_(bpy)(Cl)] (ref) was also involved. The HSV-2 DNA concentration was measured by direct qPCR. Data represent the average −*C*_t_ values ± standard deviations. {Treatment of the Vero cells: 24 h; *n* = 3}.

In the next step, Vero cells were subjected to HSV-2 infection (at a multiplicity of infection (MOI) of 0.1), followed by treatment with the complexes at varying concentrations for 24 h. Then the cells were lysed, and the antiviral efficacy of the compounds was evaluated by comparing the viral yield reduction against untreated Vero cells. The HSV-2 growth was monitored by direct quantitative polymerase chain reaction (qPCR) analysis. The higher cycle threshold (*C*_t_) values mean that more PCR cycles were required to detect the target nucleic acid, indicating stronger antiviral activity. The complexes capable of inhibiting HSV-2 growth were **1**–**4Cl** and **3Br**, while the aqua complexes were much less effective (see the average *C*_t_ values obtained for **1**–**4Cl** and **3Br** at the different complex concentrations in Figure 7 and for **1**–**4Aq** in Figure S35).

In this case, the IC_50_ value means the complex concentration at which the DNA content decreased by 50%, interpreted as the concentration at which the qPCR Ct value increased by ca. one cycle. The complex concentration that inhibited the HSV-2 growth by 90% (IC_90_) increased the *C*_t_ value by ca. 3.32 cycles. The maximum HSV-2 growth corresponded to a DNA concentration of *C*_t_ ca. 15.5 as detected by direct qPCR. Inhibition curves showed that the most potent complexes were **4Cl** and **3Br** (Figure 7), exceeding the activity of the reference complex *fac*-[Re(CO)_3_(bpy)(Cl)]. In the case of both complexes, the IC_50_ were *c.a.* 50 μM and IC_90_ was between 50 and 100 μM.

The direct impact of complexes on DNA polymerase of qPCR was also examined to ensure the reliability of our antiviral studies (Figure S36), since metal ions and metal complexes can affect the qPCR reaction by interacting with the polymerase enzyme.^30^ Based on the similar *C*_t_ (and relatively low d*C*_t_) values obtained for the treated and untreated samples, there is no stimulation or inhibition during the qPCR.

## Conclusions

Twelve new tricarbonylrhenium(I) complexes with the general formula *fac*-[Re(CO)_3_(NN)X]^n+^ (X = Cl^–^, Br^–^ or H_2_O and *n* = 0 or 1) were synthesized and characterized. X-ray crystallographic analysis of the complexes revealed the expected chelating binding mode of the bidentate nitrogen ligands, and the coordination sphere was completed by the binding of a chlorido, bromido, or aqua ligand.

The behavior of the Re(CO)_3_ complexes was studied in water and in different biological matrices such as cell culture medium and blood serum to get deeper insights into their stability and the different chemical forms present in solution. The compounds were found to be stable in different biological matrices and no dissociation of the bidentate ligands and/or carbonyl ligands was observed. This finding suggests rather high stability, but the coordinated ligands were not intact, as hydrolysis of the ester bonds was detected over time at pH = 7.4, which decreased the lipophilicity and thus possibly the cytotoxic activity when **1Cl** was compared to the *fac*-[Re(CO)_3_(bpy)(Cl)]. The complexes were stable over a wide pH range, and when the pH was lowered to 2 there was no evidence of release of the bidentate ligand, even at the relatively low concentrations, which hindered the determination of their formation constants. However, the coordinated water coligand deprotonates at pH > 6 (p*K*_a_ = 7.8–8.1), and based on the determined p*K*_a_ values (7.8–8.1), the formation of mixed hydroxido complexes is significant (18–30%) at pH 7.4. The halido complexes transform into the corresponding aqua complexes in an aqueous solution, which is consistent with the relatively low chloride ion affinity of the aqua complexes.

The organorhenium complexes with the halido ligands (**1**–**4Cl**, **3Br**) showed weak cytotoxic activity on the Colo205 (IC_50_ = 60–96 μM) and the resistant Colo320 (IC_50_ = 73–99 μM) cancer cell lines and exhibited moderate antiviral activity against *Herpes simplex* virus type 2. It was found that the presence of a thiazole moiety in the complexes can increase the cytotoxicity. However, the complexes with the aqua coligand (**1**–**4Aq**) displayed even weaker cytotoxicity (IC_50_ > 100 μM) and antiviral activity. However, on the basis of the solution studies, the dissolution of the halido complexes also results in aqua complexes, but this process is not spontaneous. Therefore, the somewhat stronger bioactivity of the halido complexes might be the result of their kinetic inertness compared to that of the aqua species, which may hinder their immediate interaction with bioligands in the cell culture medium.

This study represents the first systematic investigation of the solution speciation of tricarbonyl organorhenium(I) complexes bearing bipyridine analogues. This type of information is crucial for the interpretation of the pharmacological effects of these compounds, and it helps elucidate the relationships between the solution speciation and biological properties and also helps the design and development of a next-generation of compounds.

## Materials and Methods

### Chemical and General Information

All starting materials for the synthesis were purchased from commercial sources (Merck, Fluorochem, and Strem Chemicals) and were used as received. Solvents for the reactions were dried over sodium sulfate and molecular sieves, while solvents for isolation of the compounds were used without further purification or drying. All other solvents were of analytical grade and used without further purification. KOH, HEPES, D_2_O, EMEM media, doxorubicin, and human serum (from human male AB plasma) were Merck (St. Louis, MO, USA) products and were used without further purification. DMSO, KCl, KNO_3_, HCl, HNO_3_, NaH_2_PO_4_, Na_2_HPO_4_, KH_2_PO_4_, and *n*-octanol were Molar Chemicals (Halásztelek, Hungary) products. DMSO-*d*_*6*_ was purchased from VWR Chemicals (Radnor, Pennsylvania, USA).

NMR spectroscopy was performed on a Bruker Avance III 500 spectrometer or Bruker Avance Neo 600 spectrometer at room temperature. ^1^H NMR spectra were recorded at 500 and 600 MHz. Chemical shifts are referenced to residual peaks of the deuterated solvent CDCl_3_, CD_3_CN at 7.26 and 1.94 ppm, 1.94 ppm respectively. Chemical shifts (δ) and coupling constants (*J*) are given in ppm and Hz, respectively. All NMR data were processed using MestRe-Nova version 11.0.4 or 14.3.0. Infrared spectra were recorded on a PerkinElmer Spectrum Two with ATR. High-resolution mass spectra (HRMS) were recorded on an Agilent 6224 Accurate Mass TOF LC/MS instrument. Elemental analyses (CHN) were carried out on a PerkinElmer 2400 II instrument. UV/vis spectra were collected on a PerkinElmer Lambda 750 UV/vis/near-IR spectrophotometer. IR and UV–vis data were processed using Spectragryph.^31^

Single crystal X-ray diffraction data was collected at 150 K on a SuperNova diffractometer with Atlas detector using CrysAlis software with monochromated Mo Kα (0.71073 Å).^32^ The initial structural models were solved with direct methods implemented in SHELXT using the *Olex*2 graphical user interface.^33^ A full-matrix least-squares refinement on *F*^2^ magnitudes with anisotropic displacement parameters for all non-hydrogen atoms using *Olex*2 or *SHELXL*-2018/3 was performed.^33,34^ All non-hydrogen atoms were refined anisotropically, while hydrogen atoms were placed at calculated positions and treated as riding on their parent atoms. Details on the crystal data, data acquisition, and refinement are presented in Table S2–S4. Mercury^35^ was used for the preparation of the figures. CCDC 2366664–2366671 contains the supplementary crystallographic data for this paper.

### General Procedure for The Synthesis of Ligands

Chelating pyridine dicarboxylic acid methyl ester ligands (pdce) were synthesized according to the literature.^14,23^ The starting 2-acetylheterocycle was mixed with *N*,*N*-dimethylformamide dimethyl acetal (DMF-DMA, 1.2 mol equiv) in 20 mL of dry toluene, and the reaction mixture was refluxed for 24 h. The respective enaminone was isolated by removing the toluene under reduced pressure and filtering the solid product with vacuum filtration. The product was washed with *n*-hexane and dried for 1 h at 45 °C. The enaminone was then refluxed with dimethyl acetylenedicarboxylate (DMAD, 2 molar equiv) in 20 mL of acetonitrile for 3 h. After the reaction was finished the reaction mixture was concentrated under reduced pressure and purified with column chromatography on silica gel. The product was eluted with the mobile phase ethyl acetate/petrol ether in the ratio of 2:1. After combining of the appropriate fractions, the mobile phase was evaporated under reduced pressure. To the product 15 mL of methanol was added together with ammonium acetate (10 mol equiv) and the reaction was stirred at room temperature overnight. The formed precipitate was filtered with vacuum filtration and washed with distilled water and *n*-hexane. The obtained product was dried overnight at 45 °C.

### General Procedure for the Synthesis of the *fac*-Tricarbonylrhenium(I) Halido Complexes with General Formula *fac*-[Re(CO)_3_(NN)X] X = Cl or Br

Rhenium complexes were synthesized according to the literature procedure.^24,25^ A reaction mixture containing the appropriate ligand **L1**–**L4** (0.2 mmol), a metal precursor [Re(CO)_5_X] (X = Cl or Br; 0.2 mmol), and 10 mL of dry toluene was heated to 120 °C in 20 mL high-pressure tubes for 2 h. The reaction mixtures were then cooled in an ice bath, and the precipitates formed were collected by vacuum filtration and washed with *n*-hexane. The products were left to dry overnight at 45 °C.

#### *fac*-[Re(CO)_3_**(L1)Cl**]

**1Cl** Yield 73.9% (282 mg), red-orange solid. ^1^H NMR (500 MHz, acetonitrile-*d*_3_) δ = 9.32 (d, *J* = 0.7 Hz, 1H), 9.06 (ddd, *J* = 5.4, 1.6, 0.8 Hz, 1H), 8.62 (d, *J* = 0.7 Hz, 1H), 8.51 (dt, *J* = 8.2, 1.1 Hz, 1H), 8.25 (td, *J* = 7.9, 1.5 Hz, 1H), 7.71 (ddd, *J* = 7.7, 5.4, 1.2 Hz, 1H), 3.98 (s, 3H), 3.97 (s, 3H) ppm. IR selected bands (ATR): ú = 2018, 1930, 1892, 1871, 1739, 1437, 1392, 1270, 1145, 1096, 959, 833, 784, 642, 628, 482 cm^–1^. UV–vis (λ (nm) (ε (M^–1^cm^–1^)), *c* = 5 × 10^–5^ M, CH_2_Cl_2_): 307.35 (25 400), 429.27 (4000). ESI-HRMS (acetonitrile) for [M–Cl + CH_3_CN]^+^ C_19_H_15_N_3_O_7_Re^+^ found: 584.0454 (calculated: 584.0468). Elemental analysis for C_17_H_12_ClN_2_O_7_Re calculated (%): C: 35.33 H: 2.09 N; 4.85; found (%): C: 35.23; H, 1.93; N: 4.71.

#### *fac*-[Re(CO)_3_**(L2)Cl**]

**2Cl** Yield 77.6% (89.9 mg), red solid. ^1^H NMR (500 MHz, acetonitrile-*d*_3_) δ = 9.72 (d, *J* = 1.3 Hz, 1H), 9.35 (d, *J* = 0.8 Hz, 1H), 9.03 (dd, *J* = 3.0, 1.3 Hz, 1H), 8.89 (d, *J* = 3.0 Hz, 1H), 8.77 (d, *J* = 0.7 Hz, 1H), 4.00 (s, 3H), 3.98 (s, 3H) ppm. IR selected bands (ATR): ú = 2021, 1939, 1926, 1887, 1727, 1438, 1281, 1242, 1144, 788, 480 cm^–1^. UV–vis (λ (nm) (ε (M^–1^cm^–1^)), *c* = 2.5 × 10^–5^ M, CH_2_Cl_2_): 313.62 (30 000), 455.87 (4800). ESI-HRMS (acetonitrile) for [M–Cl + CH_3_CN]^+^ C_18_H_14_N_4_O_7_Re^+^ found: 585.0410 (calculated: 585.0420). Elemental analysis for C_16_H_11_ClN_3_O_7_Re calculated (%): C: 33.19; H, 1.92; N, 7.29; found (%): C: 33.31; H: 1.79; N: 7.21. Single crystals were obtained by slow evaporation from a mixture of acetone and water.

#### *fac*-[Re(CO)_3_**(L3)Cl**]

**3Cl** Yield 89.3% (104.3 mg), red solid. ^1^H NMR (500 MHz, acetonitrile-*d*_3_) δ = 9.30 (d, *J* = 0.7 Hz, 1H), 8.41 (d, *J* = 0.7 Hz, 1H), 8.24 (d, *J* = 3.3 Hz, 1H), 8.05 (d, *J* = 3.3 Hz, 1H), 3.97 (s, 3H), 3.97 (s, 3H) ppm. IR selected bands (ATR): ú = 2024, 1889, 1734, 1615, 1545, 1438, 1300, 1142, 1092, 781, 737, 524, 481 cm^–1^. UV–vis (λ (nm) (ε (M^–1^cm^–1^)), *c* = 4 × 10^–5^ M, CH_2_Cl_2_): 326.80 (24500), 444.66 (4750). ESI-HRMS (acetonitrile) for [M-Cl + CH_3_CN]^+^ C_17_H_13_N_3_O_7_ReS^+^ found: 590.0019 (calculated: 590.0032). Elemental analysis for C_15_H_10_ClN_2_O_7_ReS calculated (%): C: 30.85; H, 1.73; N, 4.80; found (%): C: 30.80; H: 1.69; N: 4.94. Single crystals were obtained from the solution of chloroform with vapor diffusion of *n*-heptane utilizing the CrystalBreeder multireactor crystallization platform.^27^

#### *fac*-[Re(CO)_3_**(L4)Cl**]

**4Cl** Yield 59.0% (70.6 mg), red solid. ^1^H NMR (500 MHz, CDCl_3_) δ = 9.39 (d, *J* = 0.7 Hz, 1H), 8.09 (s, 1H), 7.43 (d, *J* = 1.1 Hz, 1H), 4.04 (s, 3H), 4.03 (s, 3H), 2.82 (d, *J* = 0.9 Hz, 3H) ppm. IR selected bands (ATR): ú = 2023, 1898, 1867, 1730, 1434, 1280, 1261, 1091, 777, 645, 527, 474 cm^–1^. UV–vis (λ (nm) (ε (M^–1^cm^–1^)), *c* = 5 × 10^–5^ M, CH_2_Cl_2_): 341.14 (18 600), 438.08 (3800). ESI-HRMS (acetonitrile) for [M-Cl + CH_3_CN]^+^ C_18_H_15_N_3_O_7_ReS^+^ found: 604.016 (calculated: 604.0188). Elemental analysis for C_16_H_12_ClN_2_O_7_ReS calculated (%): C: 32.14; H, 2.02; N, 4.68; found (%): C: 32.55; H: 1.85; N: 4.63. Single crystals were obtained from the solution of chloroform with vapor diffusion of *n*-heptane utilizing the CrystalBreeder multireactor crystallization platform.^27^

#### *fac*-[Re(CO)_3_**(L1)Br**]

**1Br** Yield 36.8% (45.8 mg), orange solid. ^1^H NMR (500 MHz, CDCl_3_) δ = 9.43 (d, *J* = 0.6 Hz, 1H), 9.13 (ddd, *J* = 5.5, 1.6, 0.8 Hz, 1H), 8.35 (d, *J* = 0.7 Hz, 1H), 8.30 (dt, *J* = 8.3, 1.0 Hz, 1H), 8.13 (td, *J* = 7.9, 1.6 Hz, 1H), 7.64 (ddd, *J* = 7.7, 5.5, 1.3 Hz, 1H), 4.04 (d, *J* = 6.3 Hz, 6H) ppm. IR selected bands (ATR): ú = 2019, 1874, 1739, 1601, 1550, 1436, 1271, 1145, 1096, 783, 640, 635, 533, 481 cm^–1^. UV–vis (λ (nm) (ε (M^–1^cm^–1^)), *c* = 5 × 10^–5^ M, CH_2_Cl_2_): 308 (21 600), 437 (3200). ESI-HRMS (acetonitrile) for [M-Br + CH_3_CN]^+^ C_19_H_15_N_3_O_7_Re^+^ found: 584.0460 (calculated: 584.0468). Elemental analysis for C_17_H_12_BrN_2_O_7_Re calculated (%): C: 32.81 H: 1.49 N; 4.50; found (%): C: 32.88; H: 1.58; N: 4.55.

#### *fac*-[Re(CO)_3_**(L2)Br**]

**2Br** Yield 75.9% (94.6 mg), orange solid. ^1^H NMR (500 MHz, CDCl_3_) δ = 9.60 (d, *J* = 1.3 Hz, 1H), 9.44 (d, *J* = 0.6 Hz, 1H), 9.07 (dd, *J* = 3.0, 1.3 Hz, 1H), 8.87 (d, *J* = 3.0 Hz, 1H), 8.50 (s, 1H), 4.06 (d, *J* = 1.6 Hz, 6H) ppm. IR selected bands (ATR): ú = 2022, 1925, 1888, 1727, 1438, 1278, 1107, 854, 638, 459 cm^–1^. UV–vis (λ (nm) (ε (M^–1^cm^–1^)), *c* = 5 × 10^–5^ M, CH_2_Cl_2_): 314 (22 200), 462 (3400). ESI-HRMS (acetonitrile) for [M-Br + CH_3_CN]^+^ C_18_H_14_N_4_O_7_Re^+^ found: 584.0407 (calculated: 585.0420). Elemental analysis for C_16_H_11_BrN_3_O_7_Re calculated (%): C: 30.83 H: 1.78 N; 6.74; found (%): C: 30.97; H: 1.65; N: 6.65.

#### *fac*-[Re(CO)_3_**(L3)Br**]

**3Br** Yield 83.6% (105.5 mg), orange solid. ^1^H NMR (500 MHz, CDCl_3_) δ = 9.41 (d, *J* = 0.7 Hz, 1H), 8.26 (d, *J* = 3.3 Hz, 1H), 8.13 (d, *J* = 0.8 Hz, 1H), 7.85 (d, *J* = 3.3 Hz, 1H), 4.04 (d, *J* = 5.1 Hz, 6H) ppm. IR selected bands (ATR): ú = 2019, 1906, 1737, 1615, 1435, 1302, 1264, 1142, 1093, 768, 645, 531, 488 cm^–1^. UV–vis (λ (nm) (ε (M^–1^cm^–1^)), *c* = 5 × 10^–5^ M, dichloromethane): 327 (19 000), 452 (3600). ESI-HRMS (acetonitrile) for [M-Br + CH_3_CN]^+^ C_17_H_13_N_3_O_7_ReS^+^ found: 590.0018 (calculated: 590.0032). Elemental analysis for C_15_H_10_BrN_2_O_7_ReS calculated (%): C: 28.67 H: 1.60 N; 4.46; found (%): C: 28.76; H: 1.29; N: 4.46. Single crystals were obtained by the slow evaporation from toluene.

#### *fac*-[Re(CO)_3_**(L4)Br**]

**4Br** Yield: 69.1% (90.5 mg); orange-brown solid. ^1^H NMR (500 MHz, CDCl_3_) δ = 9.41 (d, *J* = 0.7 Hz, 1H), 8.10 (d, *J* = 0.8 Hz, 1H), 7.44 (d, *J* = 0.9 Hz, 1H), 4.03 (d, *J* = 6.4 Hz, 7H), 2.82 (d, *J* = 0.9 Hz, 3H) ppm. IR selected bands (ATR): ú = 2022, 1801, 1930, 1733, 1614, 1435, 1291, 1141, 1093, 951, 794, 644, 486 cm^–1^. UV–vis (λ (nm) (ε (M^–1^cm^–1^)), *c* = 5 × 10^–5^ M, CH_2_Cl_2_): 342 (17200), 444 (3200). ESI-HRMS (acetonitrile) for [M-Br + CH_3_CN]^+^ C_18_H_15_N_3_O_7_ReS^+^ found: 604.0178 (calculated: 604.0188). Elemental analysis for C_16_H_12_BrN_2_O_7_ReS calculated (%): C: 29.91 H: 1.88 N; 4.36; found (%): C: 29.65; H: 1.66; N: 4.32. Single crystals were obtained by slow evaporation from toluene.

### General Procedure for The Synthesis of *fac*-Tricarbonylrhenium(I) Aqua Complexes with General Formula *fac*-[Re(CO)_3_(NN)(H_2_O)]CF_3_SO_3_

Complexes were synthesized according to the literature procedure.^26^ A reaction mixture containing appropriate chlorido complex **1Cl–4Cl** (0.1 mmol), silver triflate (0.12 mmol), and 10 mL of dichloromethane was stirred at room temperature overnight. The precipitate of AgCl was removed by filtration over Celite, and dichloromethane was removed under reduced pressure. The product was dissolved in a mixture of methanol and water in a ratio of 99:1 and left to slowly evaporate.

#### *fac*-[Re(CO)_3_(**L1**)(H_2_O)]CF_3_SO_3_

**1Aq** Yield 28.9% (16.0 mg), red-orange solid. ^1^H NMR (500 MHz, acetonitrile-*d*_3_) δ = 9.34 (d, *J* = 0.7 Hz, 1H), 9.12–9.01 (m, 1H), 8.67 (d, *J* = 0.7 Hz, 1H), 8.56 (dt, *J* = 8.1, 1.0 Hz, 1H), 8.34 (td, *J* = 7.9, 1.5 Hz, 1H), 7.80 (ddd, *J* = 7.8, 5.5, 1.2 Hz, 1H), 4.01 (s, 3H), 4.00 (s, 3H) ppm. IR selected bands (ATR): ú = 3121, 2033, 1906, 1732, 1436, 1275, 1232, 1144, 1096, 1025, 781, 634 cm^–1^. UV–vis (λ (nm) (ε (M^–1^cm^–1^)), *c* = 5 × 10^–5^ M, methanol): 312.19 (15 200), 379.02 (3200). ESI-HRMS (acetonitrile) for [M-Cl + CH_3_CN]^+^ C_19_H_15_N_3_O_7_Re^+^ found: 584.0450 (calculated: 584.0468). Elemental analysis for C_18_H_14_F_3_N_2_O_11_ReS calculated (%): C: 30.47; H: 1.99; N: 3.95; found (%): C: 31.31; H: 1.83; N: 4.10.

#### *fac*-[Re(CO)_3_(**L2**)(H_2_O)]CF_3_SO_3_

**2Aq** Yield 26.9% (11.3 mg), red-orange solid. ^1^H NMR (500 MHz, acetonitrile-*d*_3_) δ = 9.77 (d, *J* = 1.4 Hz, 1H), 9.36 (s, 1H), 9.03 (dd, *J* = 3.1, 1.3 Hz, 1H), 9.00 (d, *J* = 3.1 Hz, 1H), 8.82 (s, 1H), 4.02 (s, 3H), 4.00 (s, 3H) ppm. IR selected bands (ATR): ú = 2925, 2036, 1910, 1732, 1437, 1276, 1235, 1144, 1027, 636, 477 cm^–1^. UV–vis (λ (nm) (ε (M^–1^cm^–1^)), *c* = 5 × 10^–5^ M, methanol): 316.08 (15 000), 397.02 (5200). ESI-HRMS (acetonitrile) for [M-Cl + CH_3_CN]^+^ C_18_H_14_N_4_O_7_Re^+^ found: 584.0405 (calculated: 585.0420). Elemental analysis for C_17_H_13_F_3_N_3_O_11_ReS·0.03 C_7_H_8_ calculated (%): C: 30.62; H, 2.04; N, 5.74; found (%): C: 30.13; H: 1.85; N: 5.78.

#### *fac*-[Re(CO)_3_(**L3**)(H_2_O)]CF_3_SO_3_

**3Aq** Yield 23.1% (14.7 mg), red-orange solid. ^1^H NMR (500 MHz, acetonitrile-*d*_3_) δ = 9.32 (d, *J* = 0.7 Hz, 1H), 8.48 (d, *J* = 0.7 Hz, 1H), 8.25 (d, *J* = 3.3 Hz, 1H), 8.16 (d, *J* = 3.3 Hz, 1H), 3.99 (s, 6H) ppm. IR selected bands (ATR): ú = 3120, 2034, 1906, 1732, 1439, 1285, 1224, 1141, 1024, 634, 526, 514 cm^–1^. UV–vis (λ (nm) (ε (M^–1^cm^–1^)), *c* = 5 × 10^–5^ M, methanol): 332.74 (16 800), 340.75 (17 000). ESI-HRMS (acetonitrile) for [M-Cl + CH_3_CN]^+^ C_17_H_13_N_3_O_7_ReS^+^ found: 590.0015 (calculated: 590.0032). Elemental analysis for C_16_H_12_F_3_N_2_O_11_ReS_2_ calculated (%): C: 26.86; H: 1.69; N: 3.92; found (%): C: 27.43; H: 1.30; N: 3.91.

#### *fac*-[Re(CO)_3_(**L4**)(H_2_O)]CF_3_SO_3_

**4Aq** Yield 45.9% (35.2 mg), red-orange solid. ^1^H NMR (500 MHz, acetonitrile-*d*_3_) δ = 9.31 (d, *J* = 0.7 Hz, 1H), 8.43 (d, *J* = 0.7 Hz, 1H), 7.80 (d, *J* = 1.0 Hz, 1H), 3.99 (s, 3H), 3.99 (s, 3H), 2.75 (d, *J* = 0.9 Hz, 3H) ppm. IR selected bands (ATR): ú = 3133, 2035, 1945, 1914, 1733, 1449, 1434, 1285, 1224, 1140, 1093, 1021, 632, 521 cm^–1^. UV–vis (λ (nm) (ε (M^–1^cm^–1^)), *c* = 5 × 10^–5^ M, methanol): 354.38 (18200). ESI-HRMS (acetonitrile) for [M-Cl + CH_3_CN]^+^ C_18_H_15_N_3_O_7_ReS^+^ found: 604.0170 (calculated: 604.0188). Elemental analysis for C_17_H_14_F_3_N_2_O_11_ReS_2_ calculated (%): C: 27.99; H, 1.93; N, 3.84; found (%): C: 28.44; H: 1.43; N: 4.01. Single crystals were obtained by slow evaporation from a mixture of methanol and water.

### Stock Solutions and Sample Preparation

Milli-Q water or DMSO was used for the preparation of stock and sample solutions. Aqueous stock solutions of aqua complexes were obtained by dissolving an exact amount in water, and their concentrations were calculated based on a weight-in-volume basis. A 4-fold dilution of human serum was carried out with PBS’ buffer for the stability measurements.

### UV–visible Spectrophotometry

An Agilent Cary 3500 spectrophotometer was utilized to obtain UV–vis spectra in the wavelength range of 200–1100 nm. The path length () was 1 cm in most cases (the actual  is always indicated in the legends of the figures). The concentrations of the complexes were between 50 and 100 μM. Spectra were always background- and baseline corrected. The computer program HypSpec^36^ was used to obtain equilibrium constants.

### Determination of Distribution Coefficients

The traditional shake-flask method was used to obtain distribution coefficients of the complexes in *n*-octanol/buffered aqueous solution (20 mM HEPES, pH = 7.4) using UV–vis spectrophotometry (Agilent Cary 3500 spectrophotometer, Santa Clara, CA, USA) for the analysis. Aqua complexes were dissolved in buffered aqueous solutions previously saturated with *n*-octanol. In the case of halido complexes, compounds were dissolved in the presaturated *n*-octanol phase due to their high lipophilicity and low aqueous solubility. Then, the aqueous and *n*-octanol phases were gently mixed in different volume ratios (using 1:1, 1:10, 1:20, 1:40 for aqua and 1:40 *n*-octanol-to-buffered aqueous solution volume ratio for halido complexes) for 4 h, followed by phase separation. Three different concentrations of chloride ions (4, 24, or 100 mM) were applied in a similar manner as described in our former reports, taking into consideration the different chloride ion concentrations found in the biofluids,^37−39^ and measurements were also done without chloride ions. Then, the UV–vis spectra of the aqueous or *n*-octanol phase were recorded and compared to a reference spectrum. Distribution coefficients (*D*_pH_) were calculated by the following equations.

When the stock solution was made in an aqueous buffer
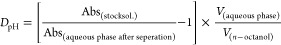


When the stock solution was made in the *n*-octanol
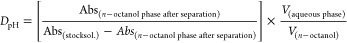


### Determination of Thermodynamic Solubility

Thermodynamic solubility (*S*_7.4_) of the halido complexes was assessed by measuring the saturation levels in water at pH = 7.4 (10 mM HEPES buffer and 0.1 M KCl) at 25.0 ± 0.1 °C. The concentration of the compounds was determined by UV–vis spectrophotometry. For the calibration, stock solutions of the compounds were used with known concentrations dissolved in 100% DMSO, 75 and 50% (v/v) DMSO/buffered aqueous solutions.

### Solution Studies Using ^1^H NMR Spectroscopy

A Bruker Avance III HD Ascend 500 Plus instrument (Billerica, MA, USA) was used for the NMR studies. Spectra were recorded with a WATERGATE water suppression pulse scheme in the presence of 10% (v/v) D_2_O in most cases. In some cases, 5–10% (v/v) DMSO-*d*_*6*_/H_2_O was also used. DSS internal standard was added to samples to obtain reference peaks. ^1^H NMR titration was carried out in the presence of 0.1 M KNO_3_. The computer program HypSpec^36^ was used to obtain equilibrium constants.

### In Vitro Cytotoxicity Assay

#### Cell Lines and Culture Conditions

Doxorubicin-sensitive Colo205 (ATCC-CCL-222) and ABCB1 (MDR1)-LRP-expressing resistant Colo320/MDR-LRP (ATCC-CCL-220.1) human colonic adenocarcinoma cell lines were purchased from LGC Promochem (Teddington, UK). The cells were cultured in RPMI 1640 medium supplemented with 10% heat-inactivated fetal bovine serum, 2 mM l-glutamine, 1 mM Na-pyruvate, and 10 mM 4-(2-hydroxyethyl)-1-piperazineethanesulfonic acid (HEPES, Sigma, Steinheim, Germany). Cell lines were incubated at 37 °C in a 5% CO_2_, 95% air atmosphere. The semiadherent human colon cancer cells were detached with Trypsin-Versene (EDTA, Sigma, Steinheim, Germany) solution for 5 min at 37 °C.

#### MTT Assay on Colo205 and Colo320 Cells

The tested compounds were dissolved in DMSO or 20% (v/v) DMSO/H_2_O mixture to prepare 5 mM stock solutions, which were diluted in complete culture medium, to study the effect of compounds on the cell growth of human colonic adenocarcinoma cell lines (doxorubicin-sensitive Colo205 and resistant Colo320 colonic adenocarcinoma cells). Doxorubicin (Merck, Darmstadt, Germany) was used as a positive control. The cells were treated with Trypsin-Versene (EDTA) solution. They were adjusted to a density of 1 × 10^4^ cells in 100 μL of the appropriate culture medium and were added to each well, with the exception of the medium control wells. Then stock solutions were diluted in the appropriate culture medium, and 2-fold serial dilutions of compounds were prepared in 100 μL of the medium, horizontally. The final volume of the wells containing compounds and cells was 200 μL. The plates containing the cancer cells were incubated at 37 °C for 72 h; at the end of the incubation period, 20 μL of MTT solution (from a stock solution of 5 mg/mL) were added to each well. After incubation at 37 °C for 4 h, 100 μL of SDS solution (10% in 0.01 M HCI) were added to each well, and the plates were further incubated at 37 °C overnight. Cell growth was determined by measuring the optical density (OD) at 540/630 nm with a Multiscan EX ELISA reader (Thermo Labsystems, Cheshire, WA, USA). Inhibition of the cell growth (expressed as IC_50_: inhibitory concentration that reduces by 50% the growth of the cells exposed to the tested compounds) was determined from the sigmoid curve where 100 – ((OD_sample_ – OD_medium control_)/(OD_cell control_ – OD_medium control_)) × 100 values were plotted against the logarithm of compound concentrations. Curves were fitted by GraphPad Prism software (2021, GraphPad Prism Software, San Diego, CA, USA)^40^ using the sigmoidal dose–response model (comparing variable and fixed slopes). The IC_50_ values were obtained from at least 3 independent experiments.

### Antibacterial Activity Assay

The following bacterial strains were used in our experiments: gram-positive *S. aureus* American Type Culture Collection (ATCC) 25923 as the methicillin-susceptible reference bacterial strain; the methicillin-resistant *S. aureus* ATCC 43300 (MRSA) strain; *E. coli* ATCC 25922 as a gram-negative bacterial strain.

MIC values of the complexes were determined in 96-well plates based on the Clinical and Laboratory Standard Institute guidelines (CLSI guidelines).^41^ The stock solutions of the compounds (dissolved in DMSO or a 20% (v/v) DMSO/H_2_O mixture in 5 mM concentration) were diluted in 100 μL of Mueller Hinton Broth. Then 10^–4^ dilutions of an overnight bacterial culture in 100 μL of the medium was added to each well with the exception of the medium control wells. The plates were further incubated at 37 °C for 18 h; at the end of the incubation period, the MIC values of tested compounds were determined by visual inspection.

### Antiviral Activity Assay

#### Cultivation and Quantification of Herpes Simplex Virus Type 2

HSV-2 strain was (gift from Dr. Ilona Mucsi, University of Szeged, Szeged, Hungary) grown in Vero (ATCC) cells and the infectivity was determined by using the plaque titration method.^42^ The virus titer was expressed as plaque-forming units (PFU).^43^

#### Culture of Vero Cells and MTT Assay

Vero cells (ATCC) were placed into the 96-well plate (Sarstedt, Nümbrecht, Germany) at a density of 4 × 10^6^ cells/plate. The cells at a density of 4 × 10^4^ cells per well were in 100 μL of minimal essential medium (MEM) (Sigma; USA) with Earle salts supplemented with 25 μg/mL gentamicin, 10% heat-inactivated fetal bovine serum (FBS) (Gibco; Germany), 8 mM HEPES, 2 mM l-glutamine, 1× nonessential amino acids, and 1 μg/mL fungisone. The cells were incubated for 60 min at room temperature (RT) to avoid the edge effect and then for 24 h at 37 °C, 5% CO_2_ that secure a 90% confluent cell layer.^44^

MTT assay was used to determine the maximum non-toxic concentration of the complexes on Vero cells. The cells were grown in 96-well plate at density of 4 × 10^4^ cells per well were in 100 μL of MEM with Earle salts supplemented with 25 μg/mL gentamicin, 10% heat-inactivated FBS, 8 mM HEPES, 2 mM l-glutamine, 1× nonessential amino acids, and 1 μg/mL fungisone. The cells were incubated for 1 h at RT and then overnight at 37 °C, 5% CO_2_. When the cell layer reached around 90% confluency the medium was complemented with the serial 2-fold dilutions of complexes. Three parallels were applied for each concentration in the range of 100–0.048 μM for each complex. 10 μL of the MTT labeling reagent was added into wells at 0.5 mg/mL final concentration. The plate was incubated for 240 min at 37 °C, 5% CO_2_ and then 100 μL of the solubilization solution (10% SDS in 1 M HCl) was added to each well. Next day the absorbance of the wells was determined by a microtiter plate reader (Labsystems Multiskan Ex 355, Thermo Fisher Scientific, Waltham, MA USA). The absorbance of the formazan product was measured at 540 nm.^45^

#### Investigation of The Impact of Complexes on HSV-2 Growth in Vero Cells

The Vero cells were transferred into the wells of the 96-well plate at a density of 4 × 10^4^ cells/well in 100 μL of Dulbecco’s Modified Eagle’s Medium (DMEM) (Sigma; USA) containing 100 U/mL penicillin, 100 mg/mL streptomycin sulfate, 5% FBS, 0.25 g/mL amphotericin B and 0.14% NaHCO_3_. Prior to infection, the cells were washed with PBS and then were incubated with HSV-2 (MOI 0.1) for 60 min at 37 °C under a 5% CO_2_ atmosphere. Then the cells were washed with PBS again, and the culture medium was complemented with the serial 2-fold dilutions of the complexes was added to triplicate wells in the concentration range of 100–0.078 μM. The plates were incubated at 37 °C, 5% CO_2_ for 24 h. The plates were evaluated with RT-qPCR.

#### Cell Lysis and Direct Quantitative PCR

The supernatans of infected cells were removed and washed with PBS twice, and then 100 μL of high-quality ultrapure water was added to wells at the end of 24 h infection. The cells were subjected to two freeze–thaw cycles. Templates for qPCR reactions originated from mixed cell lysates. Process of PCR was described previously.^46^ Briefly, we used 5× HOT FIREPol EvaGreen qPCR Supermix (Solis Solis BioDyne, Tartu, Estonia) and HSV-2 gD2 gene specific primer in a Bio-Rad CFX96 real time PCR system for the qPCR reaction. Sequences of the gD2 gene specific primer pair were the following: gD2-F: 5′-TCAGCGAGGATAACCTGGGA-3′, and gD2-R 5′-GGGAGAGCGTACTTGCAGGA-3′. Annealing-extension temperature was 69 °C. Cycle where the amplification curve stepped over the baseline can correspond to the Ct value of a given sample.

## Supporting Information Available

Chemical structure of the ligands and the *fac*-[Re(CO)_3_] complexes. ^1^H NMR spectra of the prepared complexes; picture and crystallographic data for the complexes obtained as single crystal with selected bond lengths; aqueous solubility data, time-dependent UV–vis, and ^1^H NMR spectra of the halido and aqua complexes in different biologically relevant matrices; UV–vis spectra of **1Aq** in water at different pH values; ratio of the aqua and hydroxido complexes at pH = 7.4; UV–vis and ^1^H NMR spectra of the aqua complex **1Aq** in the presence of chloride ions; lipophilicity data for the studied complexes; cytotoxicity of the complexes against Vero cells and antiviral effect of complexes **1–4Aq** against HSV-2 and *C*_t_ and d*C*_t_ values of the halido complexes (PDF)
X-ray data (ZIP)
NMR data (ZIP)
