# Supplementary material for: Structural and Solution Speciation Studies on fac-Tricarbonylrhenium(I) Complexes of 2,2′-Bipyridine Analogues
Source: ACS Omega. 2024 Oct 26;9(44):44601–15. doi: 10.1021/acsomega.4c07117 (PMC11541514; doi:10.1021/acsomega.4c07117)
Supplement: Supplementary file 1 — ao4c07117_si_001.pdf [file ao4c07117_si_001.pdf]

Supporting Information

Structural and solution speciation studies on fac-tricarbonylrhenium(I) complexes of 2,2'-bipyridine analogues

Tamás Pivarcsik[1,2], *Jakob Kljun*[3], *Sergio Clemente Rodriguez*[3,4], *David Cortéz Alcaraz*[3,4], *Uroš Rapuš*[3], *Márta Nové*[1,5], *Egon F. Várkonyi*[1,2], *József Nyári*[5], *Anita Bogdanov*[5], *Gabriella Spengler*[1,5], *Iztok Turel*[3,*] *Éva A. Enyedy*[1,2,*]

[1] *MTA-SZTE Lendület Functional Metal Complexes Research Group, University of Szeged, Dóm tér 7-8., H-6720 Szeged, Hungary, * Email: enyedy@chem.u-szeged.hu*

[2] *Department of Molecular and Analytical Chemistry, Interdisciplinary Excellence Centre, University of Szeged, Dóm tér 7-8., H-6720 Szeged, Hungary*

[3] *Faculty of Chemistry and Chemical Technology, University of Ljubljana, Večna pot 113, SI-1000 Ljubljana, Slovenia, * Email: Iztok.Turel@fkkt.uni-lj.si*

[4] *Universidad de Alcalá, 28805 Alcalá de Henares, Madrid, Spain*

[5] *Department of Medical Microbiology, Albert Szent-Györgyi Health Center and Albert Szent-Györgyi Medical School, University of Szeged, Semmelweis u. 6, H-6725 Szeged, Hungary*

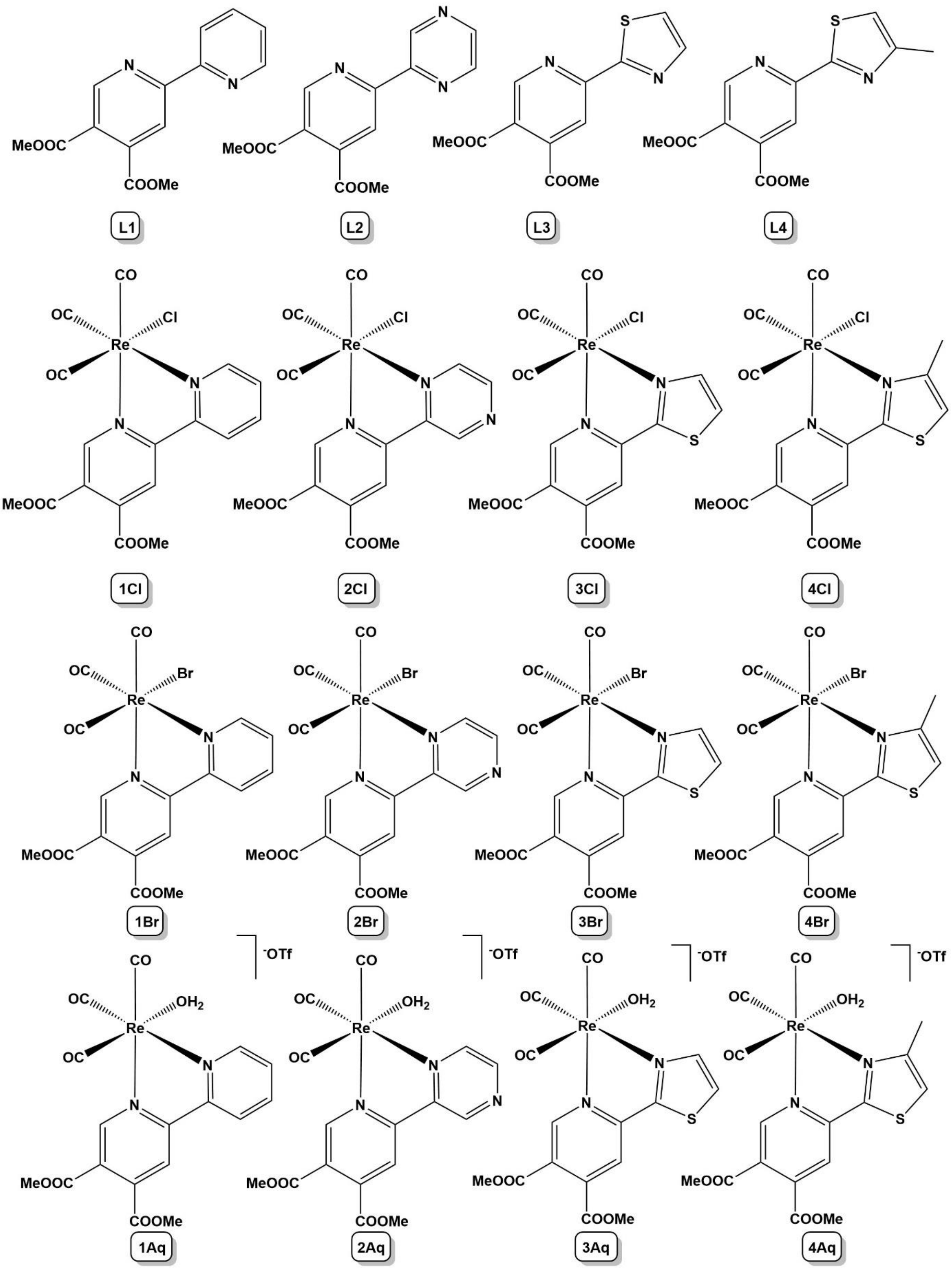

Chart S1. Chemical structure of the ligands **L1-4** and the *fac*-[Re(CO)$_3$] complexes prepared with labels.

Supporting Information

In the NMR spectra peaks of solvents can be observed besides the peaks for the compounds. Solvents present are mainly toluene (used as solvent for the reaction), acetone (used to clean NMR tubes) and silicon grease (from the NMR machine).

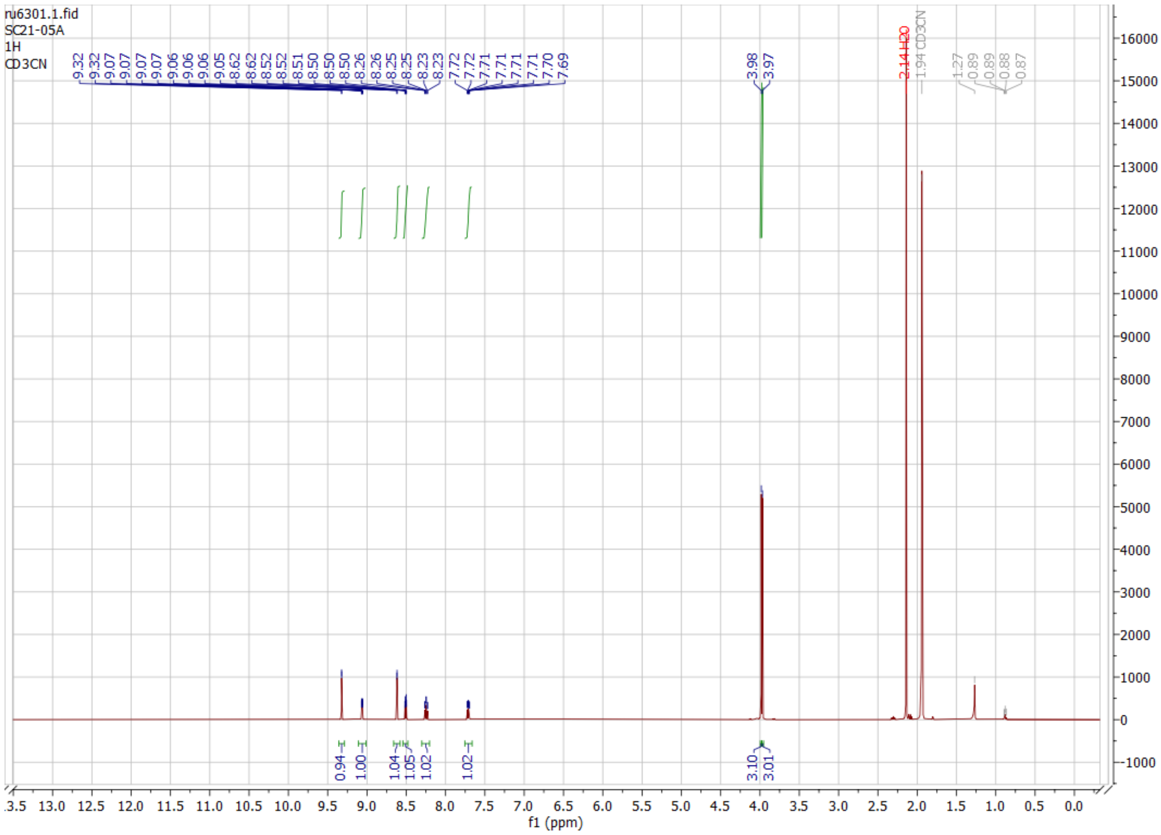

Figure S1. ^{1}H NMR of **1Cl**.

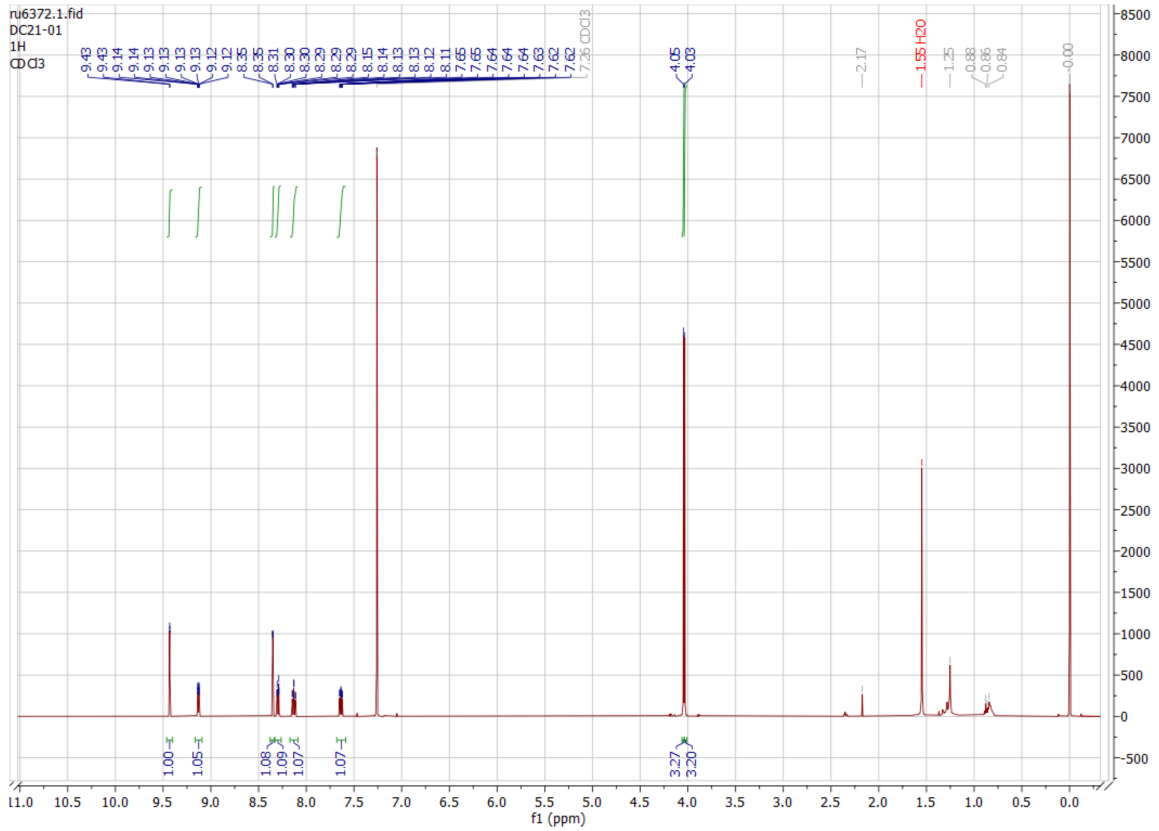

Figure S2. ^{1}H NMR of **1Br**.

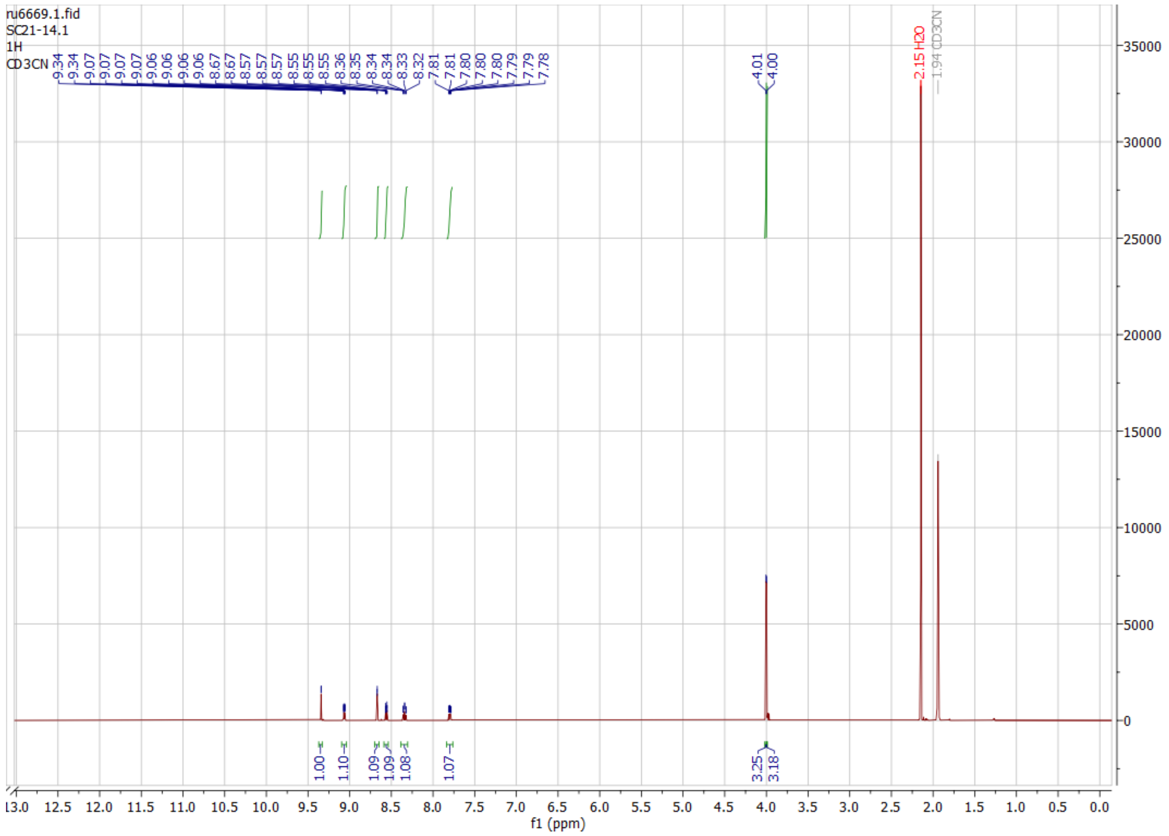

Figure S3. ^{1}H NMR of **1Aq**.

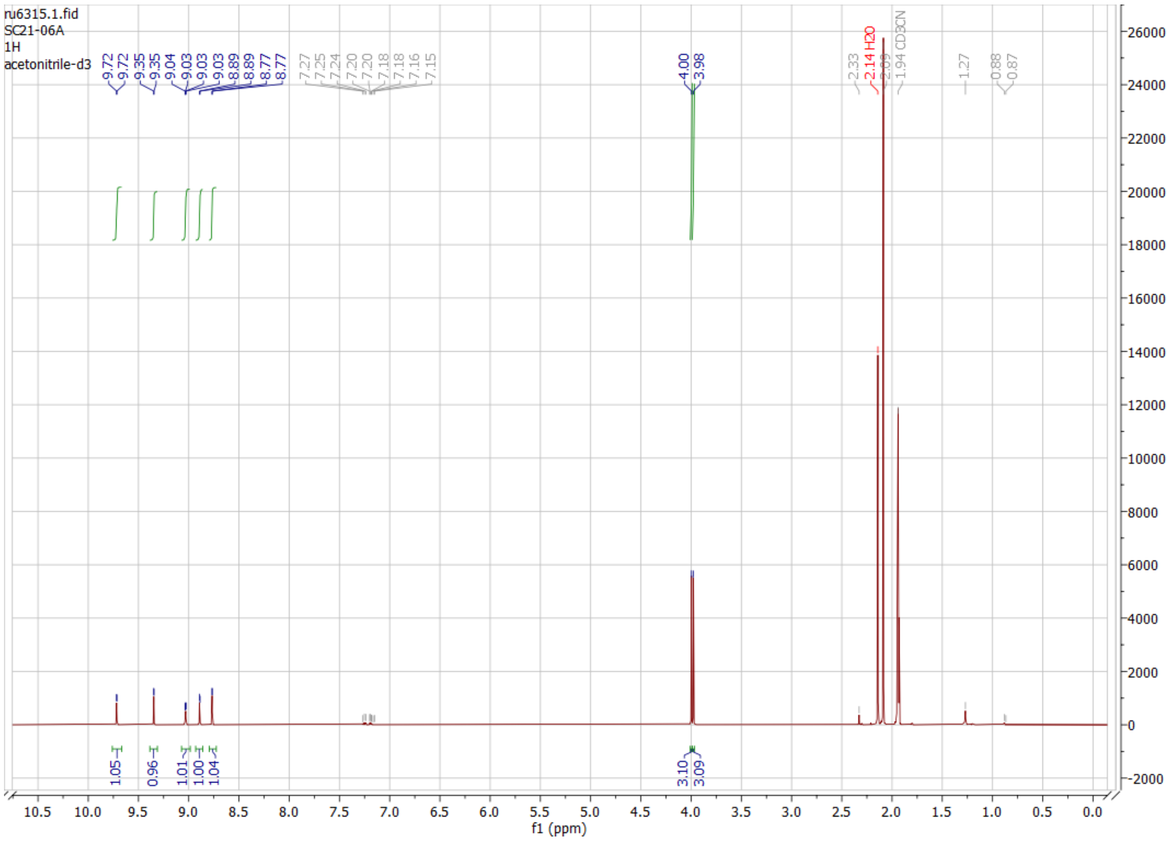

Figure S4. ^{1}H NMR of **2Cl**.

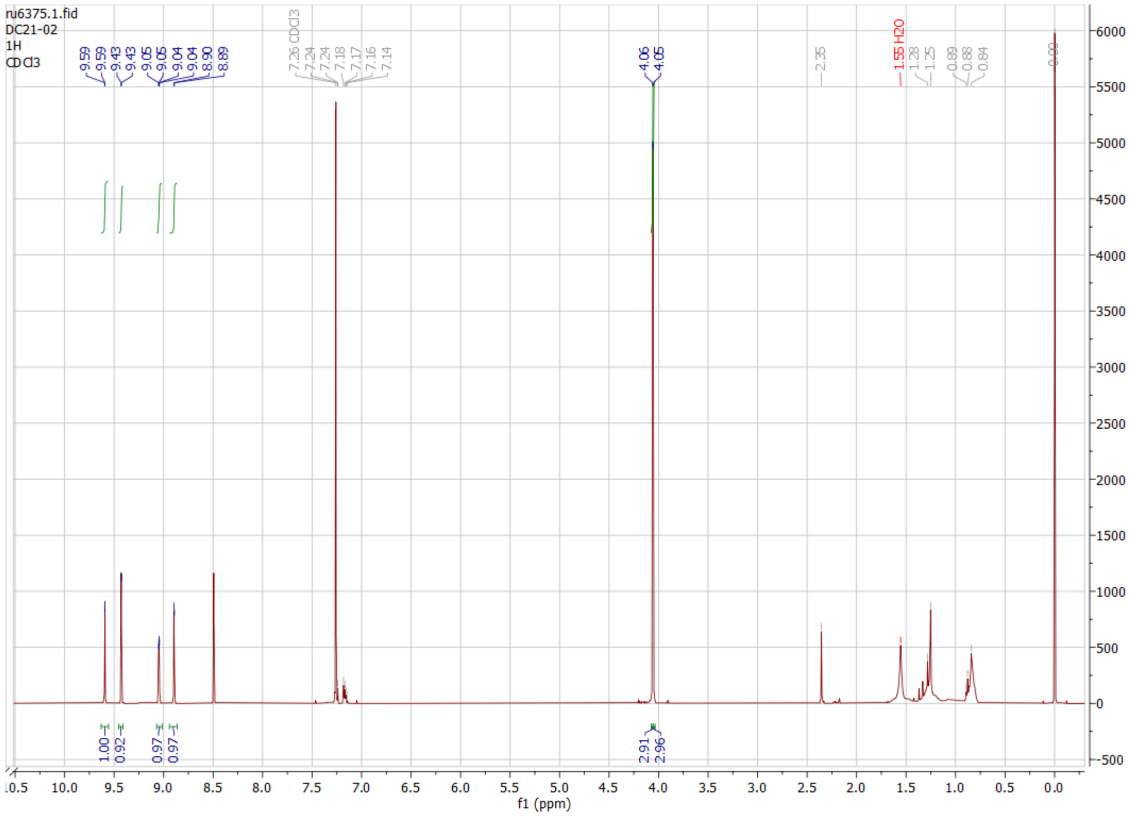

Figure S5. ^{1}H NMR of **2Br**.

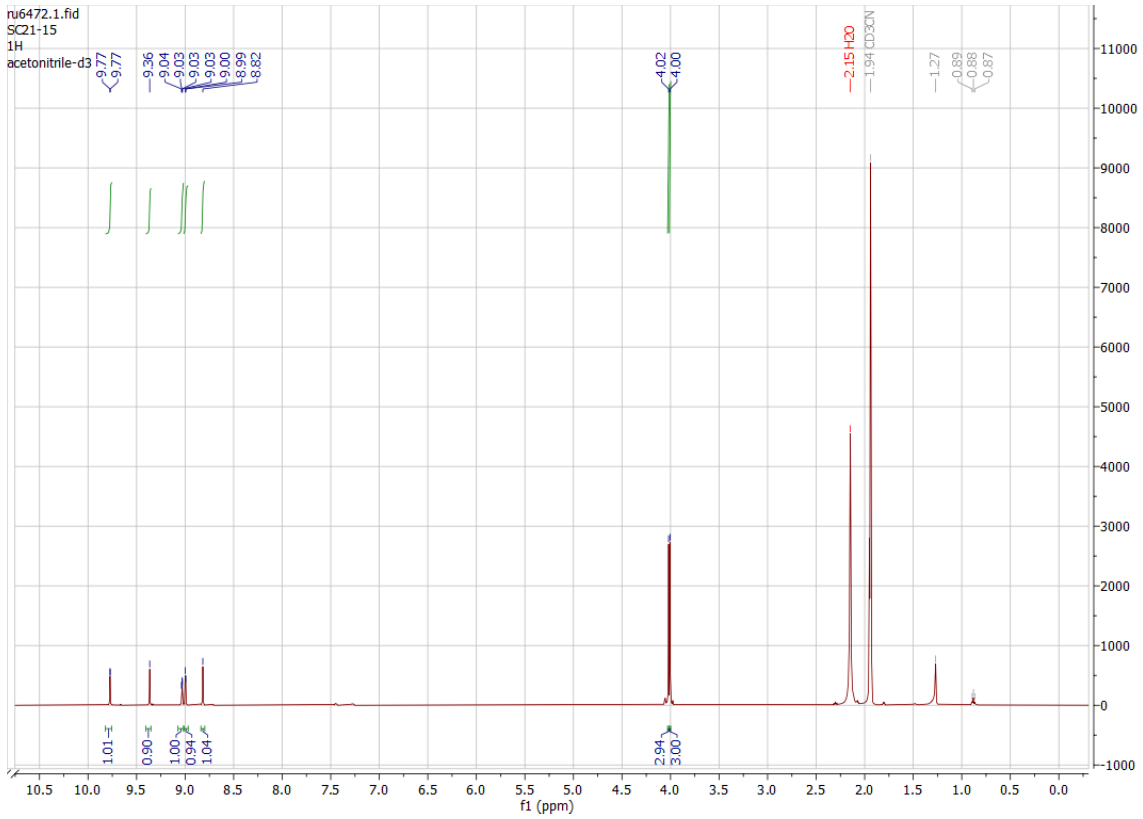

Figure S6. ^{1}H NMR of **2Aq**.

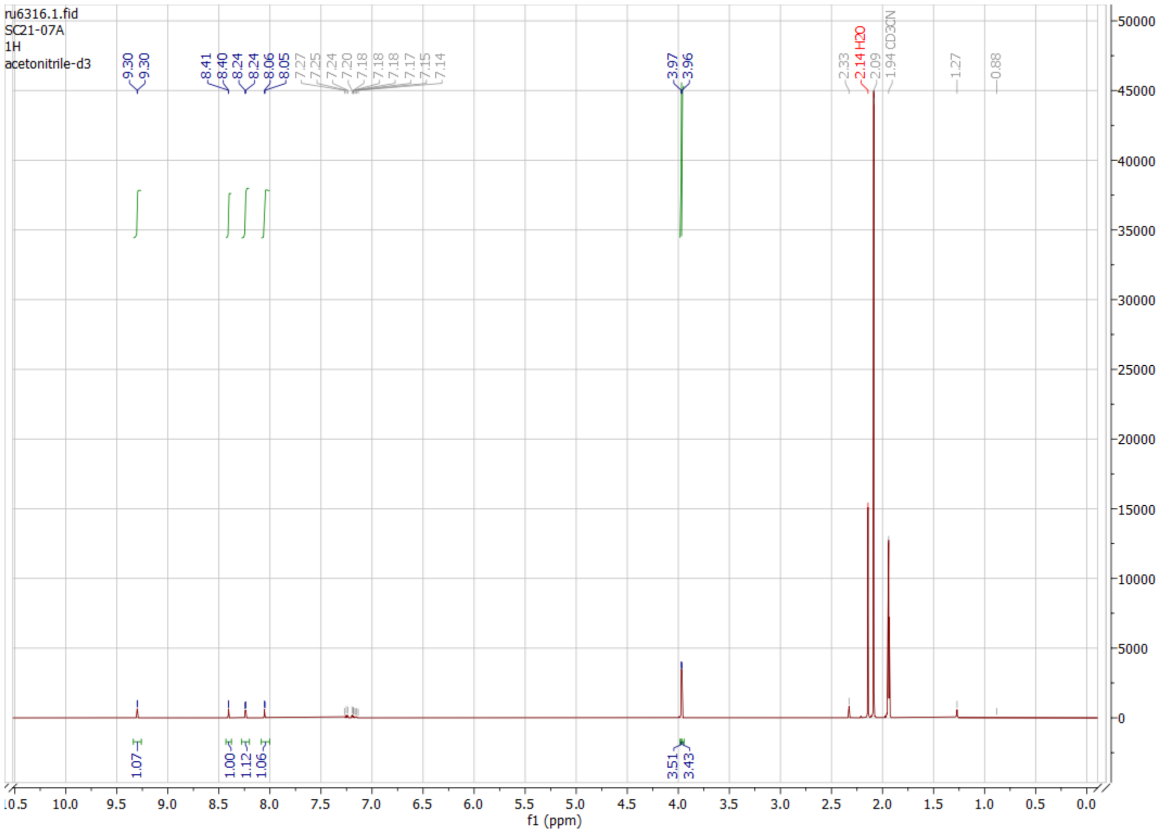

Figure S7. ^{1}H NMR of **3Cl**.

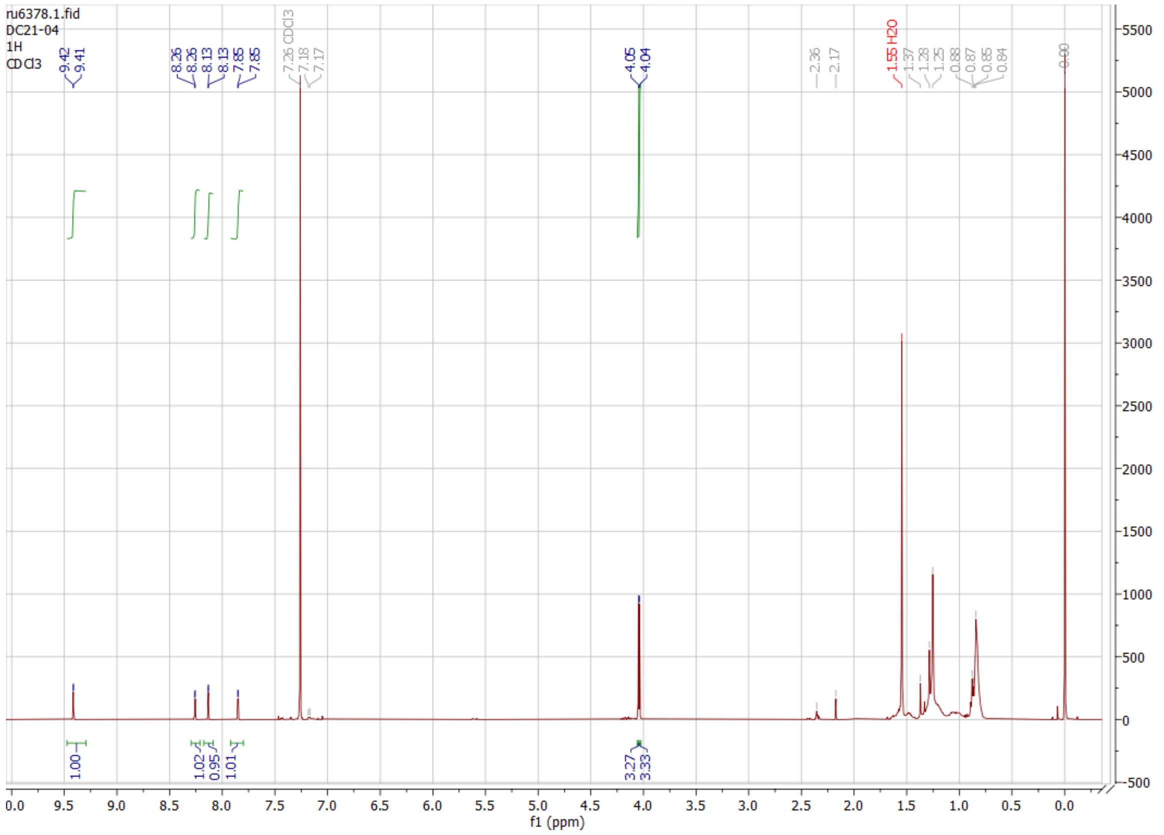

Figure S8. ^{1}H NMR of **3Br**.

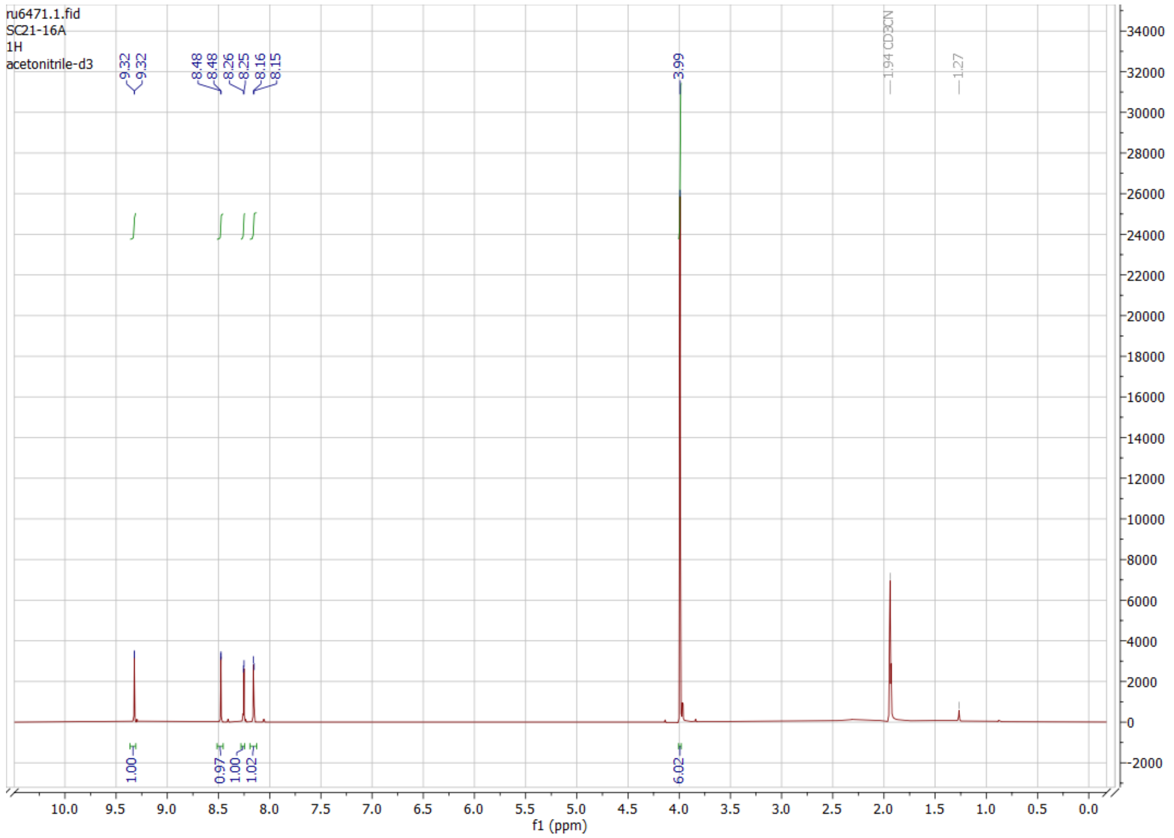

Figure S9. ^{1}H NMR of **3Aq**.

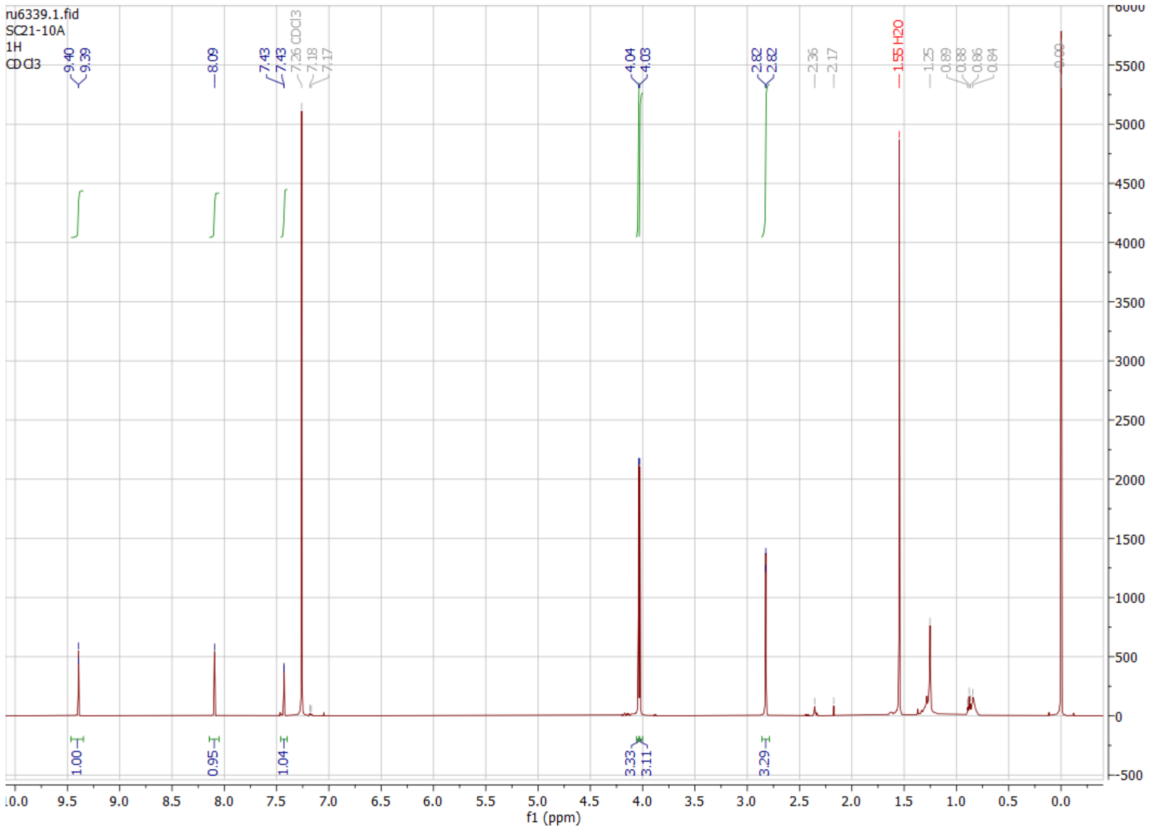

Figure S10. ^{1}H NMR of **4Cl**.

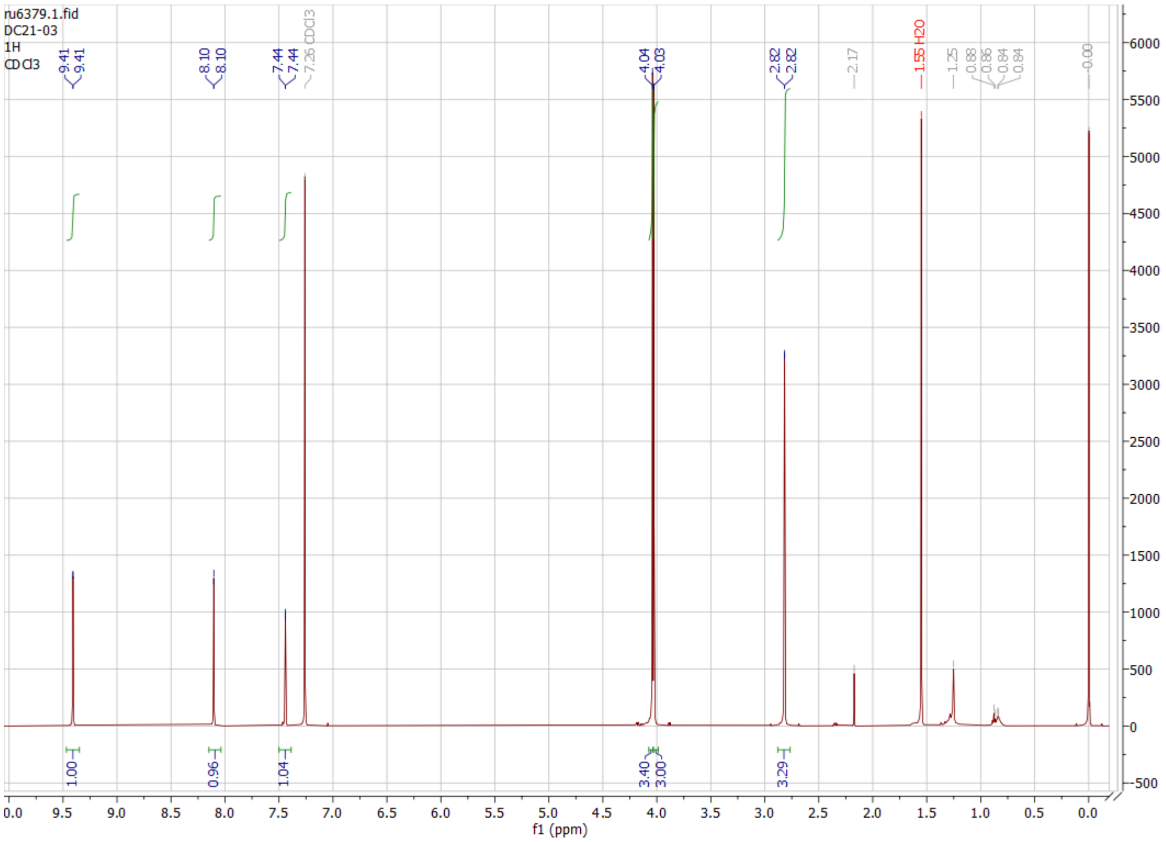

Figure S11. ^{1}H NMR of **4Br**.

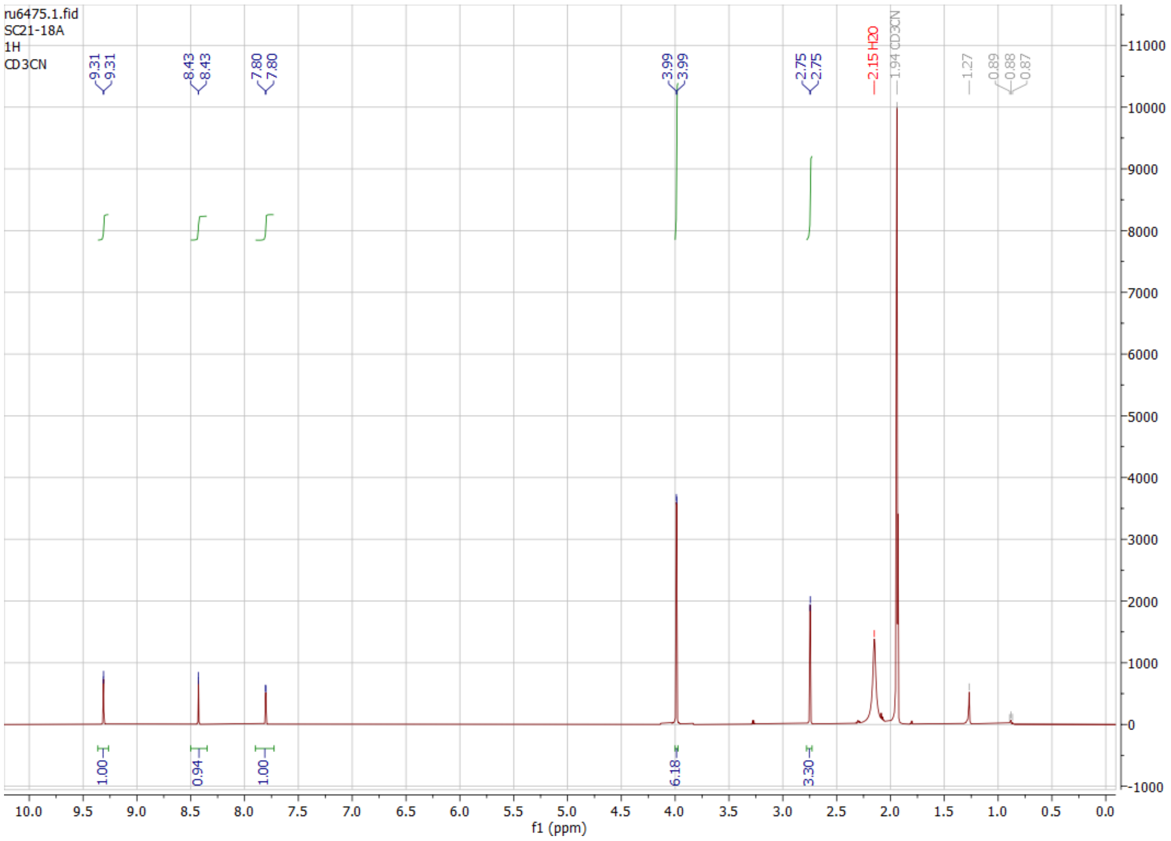

Figure S12. ^{1}H NMR of **4Aq**.

Table S1. Pictures of the analyzed single crystals.[a]

	**1**	**2**	**3**	**4**
**Cl**		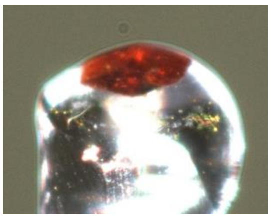	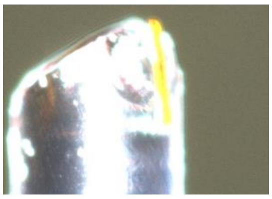	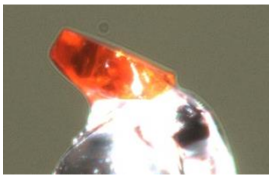
**Br**			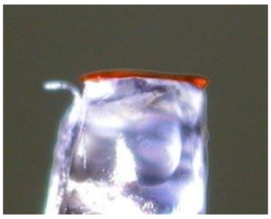	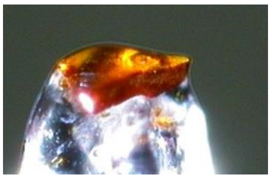
**Aq**	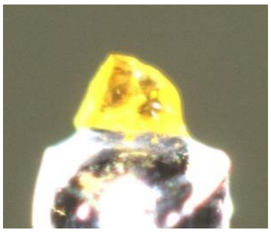		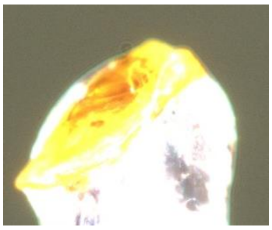	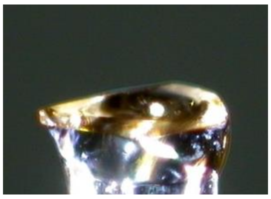

[a] The structure of **3Cl** contains a large solvent accessible void of 224 Å^3 with an integrated residual electron density of 24 e$^-$. Attempts to place a solvent molecule (acetone or multiple water molecules) were not successful.

Table S2. Crystallographic data for complexes **2Cl**, **3Cl**, and **4Cl**.

Compound	**2Cl**	**3Cl**	**4Cl**
CCDC No.	2366664	2366665	2366666
Sample code	mod25_auto	mod12_auto	mod28_auto
Empirical formula	$C_{32}H_{22}Cl_2N_6O_{14}Re_2$	$C_{15}H_{10}ClN_2O_7ReS$	$C_{16}H_{12}ClN_2O_7ReS$
Formula weight	1157.85	583.96	597.99
Temperature [K]	150.00(10)	150.00(10)	150.00(10)
Crystal system	triclinic	monoclinic	triclinic
Space group	P-1	$P2_1/n$	P-1
a [Å]	12.6956(3)	11.0635(3)	9.8309(4)
b [Å]	12.9333(3)	8.0622(2)	10.0620(3)
c [Å]	13.6408(4)	24.6138(6)	10.3204(4)
α [°]	64.949(3)	90	89.429(3)
β [°]	66.006(3)	101.704(2)	71.172(4)
γ [°]	89.370(2)	90	78.930(3)
Volume [$Å^3$]	1818.24(10)	2149.81(10)	946.79(6)
Z	2	4	2
ρ_{calc} [g cm^{-3}]	2.115	1.804	2.098
μ [mm^{-1}]	6.876	5.909	6.711
F(000)	1104.0	1112.0	572.0
Crystal size [mm^3]	0.1 × 0.1 × 0.05	0.2 × 0.025 × 0.025	0.25 × 0.25 × 0.1
Radiation	Mo Kα (λ = 0.71073)	Mo Kα (λ = 0.71073)	Mo Kα (λ = 0.71073)
2Θ range for data collection [°]	5.508 to 54.958	5.328 to 54.962	5.446 to 54.968
Index ranges	$-16 \le h \le 16$, $-16 \le k \le 16$, $-17 \le l \le 17$	$-14 \le h \le 14$, $-10 \le k \le 10$, $-31 \le l \le 31$	$-12 \le h \le 12$, $-12 \le k \le 13$, $-12 \le l \le 13$
Reflections collected	34347	47923	8794
Independent reflections	8342 [R_{int} = 0.0363, R_{sigma} = 0.0318]	4936 [R_{int} = 0.0815, R_{sigma} = 0.0442]	4341 [R_{int} = 0.0458, R_{sigma} = 0.0591]
Data/restraints/parameters	8342/0/509	4936/0/246	4341/0/250
Goodness-of-fit on F^2	1.052	1.107	1.025
Final R indexes [$I \ge 2\sigma$ (I)]	R_1 = 0.0299, wR_2 = 0.0671	R_1 = 0.0368, wR_2 = 0.0818	R_1 = 0.0319, wR_2 = 0.0650
Final R indexes [all data]	R_1 = 0.0400, wR_2 = 0.0725	R_1 = 0.0535, wR_2 = 0.0865	R_1 = 0.0384, wR_2 = 0.0686
Largest diff. peak / hole [e $Å^{-3}$]	3.48/-1.17	2.14/-0.87	2.50/-1.68

Table S3. Crystallographic data for complexes **3Br** and **4Br**.

Compound	**3Br·toluene**	**4Br**
CCDC No.	2366667	2366668
Sample code	moc177	moc179
Empirical formula	$C_{22}H_{18}BrN_2O_7ReS$	$C_{16}H_{12}BrN_2O_7ReS$
Formula weight	720.55	642.45
Temperature [K]	150.00(10)	150.00(10)
Crystal system	monoclinic	triclinic
Space group	$P2_1/n$	P-1
a [Å]	12.3403(4)	10.0127(3)
b [Å]	8.1416(2)	10.1780(3)
c [Å]	25.0932(7)	10.3325(3)
α [°]	90	87.887(2)
β [°]	102.897(3)	70.324(3)
γ [°]	90	77.293(2)
Volume [$Å^3$]	2457.51(12)	966.42(5)
Z	4	2
ρ_{calc} [g cm^{-3}]	1.948	2.208
μ [mm^{-1}]	6.700	8.504
F(000)	1384.0	608.0
Crystal size [mm^3]	0.2 × 0.05 × 0.05	0.3 × 0.25 × 0.1
Radiation	Mo Kα (λ = 0.71073)	Mo Kα (λ = 0.71073)
2Θ range for data collection [°]	5.274 to 54.966	4.966 to 54.97
Index ranges	$-15 \leq h \leq 16$, $-10 \leq k \leq 10$, $-32 \leq l \leq 32$	$-12 \leq h \leq 12$, $-13 \leq k \leq 12$, $-13 \leq l \leq 13$
Reflections collected	28107	29128
Independent reflections	5585 [R_{int} = 0.0634, R_{sigma} = 0.0552]	4424 [R_{int} = 0.0522, R_{sigma} = 0.0338]
Data/restraints/parameters	5585/0/292	4424/0/250
Goodness-of-fit on F^2	0.991	1.035
Final R indexes [$I \geq 2\sigma(I)$]	R_1 = 0.0334, wR_2 = 0.0566	R_1 = 0.0311, wR_2 = 0.0712
Final R indexes [all data]	R_1 = 0.0537, wR_2 = 0.0603	R_1 = 0.0366, wR_2 = 0.0748
Largest diff. peak / hole [e $Å^{-3}$]	1.32/-1.04	2.90/-1.98

Table S4. Crystallographic data for complexes **1Aq**, **3Aq**, and **4Aq**.

Compound	**1Aq (methanesulfonate)**	**3Aq (methanesulfonate)**	**4Aq (triflate)**
CCDC No.	2366669	2366670	2366671
Sample code	mod26_auto	mod27_auto	moc331
Empirical formula	$C_{18}H_{17}N_2O_{11}SRe$	$C_{17}H_{17}N_2O_{11}ReS_2$	$C_{17}H_{14}F_3N_2O_{11}ReS_2$
Formula weight	655.59	675.64	729.62
Temperature [K]	150.00(10)	149.5(8)	150.00(10)
Crystal system	triclinic	triclinic	triclinic
Space group	P-1	P-1	P-1
a [Å]	9.4962(2)	8.0160(2)	9.4366(3)
b [Å]	11.1763(4)	11.8334(3)	11.7454(4)
c [Å]	11.4027(4)	12.7420(3)	11.8245(4)
α [°]	110.491(3)	110.646(2)	65.301(3)
β [°]	92.619(2)	94.578(2)	83.502(3)
γ [°]	96.465(2)	94.117(2)	89.778(3)
Volume [$Å^3$]	1121.69(6)	1120.94(5)	1181.67(7)
Z	2	2	2
ρ_{calc} [g cm^{-3}]	1.941	2.002	2.051
μ [mm^{-1}]	5.571	5.668	5.402
F(000)	636.0	656.0	704.0
Crystal size [mm^3]	0.2 × 0.2 × 0.15	0.35 × 0.25 × 0.1	0.4 × 0.2 × 0.03
Radiation	Mo Kα (λ = 0.71073)	Mo Kα (λ = 0.71073)	Mo Kα (λ = 0.71073)
2Θ range for data collection [°]	5.436 to 54.966	5.128 to 54.964	5.426 to 54.968
Index ranges	$-12 \leq h \leq 12$, $-14 \leq k \leq 14$, $-14 \leq l \leq 14$	$-10 \leq h \leq 10$, $-15 \leq k \leq 15$, $-16 \leq l \leq 16$	$-12 \leq h \leq 12$, $-15 \leq k \leq 15$, $-15 \leq l \leq 15$
Reflections collected	17566	34564	25541
Independent reflections	5143 [R_{int} = 0.0419, R_{sigma} = 0.0442]	5144 [R_{int} = 0.0838, R_{sigma} = 0.0435]	5360 [R_{int} = 0.0746, R_{sigma} = 0.0507]
Data/restraints/parameters	5143/0/302	5144/0/303	5360/1/333
Goodness-of-fit on F^2	1.050	1.069	1.035
Final R indexes [I ≥ 2σ (I)]	R_1 = 0.0250, wR_2 = 0.0483	R_1 = 0.0283, wR_2 = 0.0672	R_1 = 0.0359, wR_2 = 0.0815
Final R indexes [all data]	R_1 = 0.0304, wR_2 = 0.0507	R_1 = 0.0331, wR_2 = 0.0709	R_1 = 0.0424, wR_2 = 0.0876
Largest diff. peak / hole [e $Å^{-3}$]	1.40/-1.11	2.04/-1.42	1.79/-1.43

Table S5. Selected bond lengths (Å) in *fac*-[Re(CO)$_3$] complexes and a diagram of atom notation.

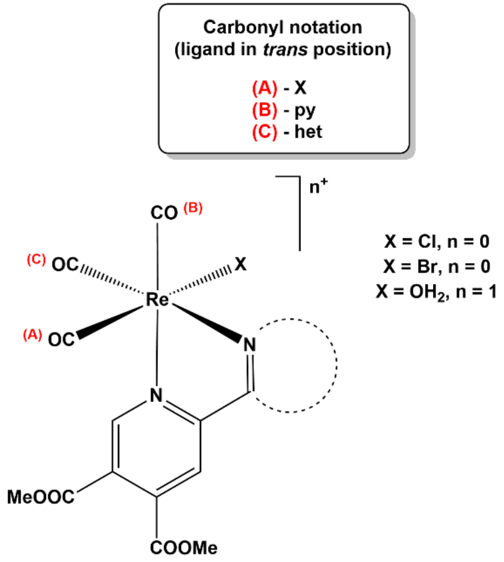

	2Cl	2Cl[a]	3Cl	4Cl	3Br	4Br	1Aq	3Aq	4Aq
Re1-C1A	1.916(6)	1.940(6)	1.906(7)	1.949(5)	1.949(6)	2.007(6)	1.896(4)	1.903(4)	1.905(6)
C1A-O2A	1.153(6)	1.120(6)	1.143(8)	1.087(5)	1.069(6)	1.008(7)	1.158(4)	1.151(5)	1.151(7)
Re1-C1B	1.915(5)	1.917(5)	1.906(6)	1.920(5)	1.911(5)	1.925(5)	1.918(4)	1.920(4)	1.909(6)
C1B-O2B	1.151(6)	1.147(6)	1.167(7)	1.150(5)	1.150(5)	1.142(6)	1.148(4)	1.143(5)	1.153(7)
Re1-C1C	1.921(5)	1.918(5)	1.920(6)	1.905(5)	1.908(5)	1.922(5)	1.916(3)	1.916(4)	1.914(6)
C1C-O2C	1.142(6)	1.146(6)	1.149(7)	1.155(5)	1.155(6)	1.136(6)	1.153(4)	1.151(5)	1.145(7)
Re1-N1$_{(py)}$	2.182(4)	2.180(4)	2.185(4)	2.194(3)	2.178(3)	2.196(4)	2.172(2)	2.176(3)	2.186(4)
Re1-N$_{het}$	2.156(4)	2.166(4)	2.154(5)	2.186(4)	2.161(4)	2.188(4)	2.164(3)	2.179(3)	2.166(4)
Re-X (X = Cl, Br, OH$_2$)	2.4758(13)	2.4637(14)	2.4773(15)	2.4621(13)	2.6109(5)	2.6024(6)	2.173(2)	2.170(3)	2.174(4)

[a] The second molecule in the asymmetric unit is marked by *.

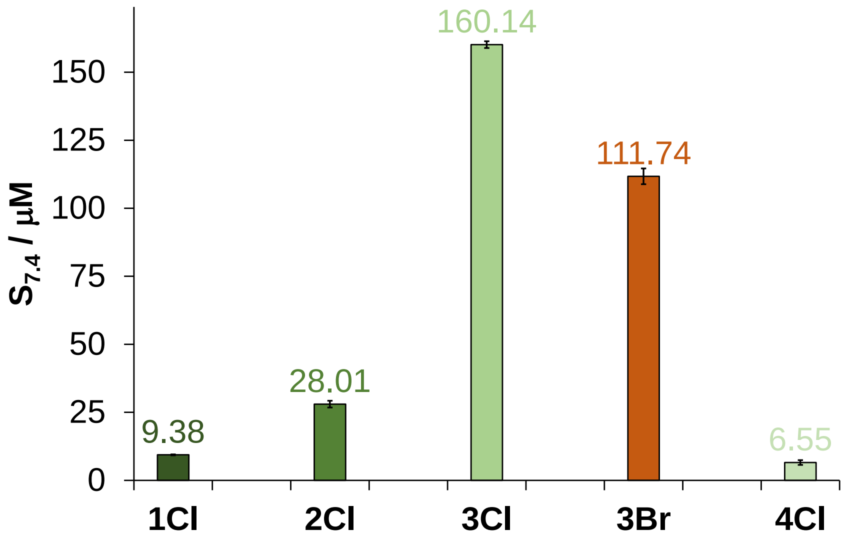

Figure S13. Aqueous solubility ($S_{7.4}$) of halido complexes at pH = 7.4 measured after a waiting time of 24 h. {20 mM HEPES buffer; 0.1 M KCl; T = 25.0 °C}

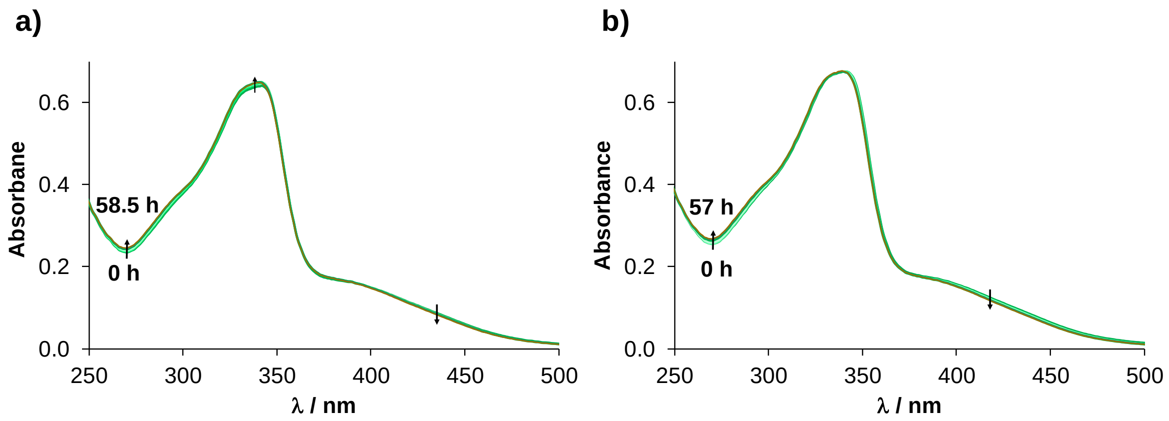

Figure S14. UV-vis spectra of (a) chlorido complex **3Cl** and (b) bromido complex **3Br** in aqueous solution over time. {$c_{complex}$= 55 µM; pH = 6.1; ℓ = 1 cm; T = 25.0 °C}

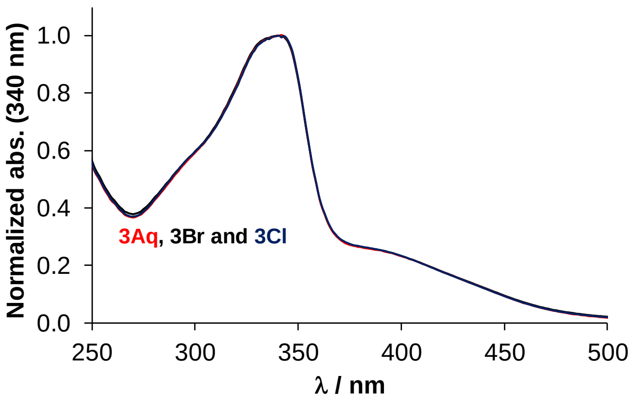

Figure S15. Normalized UV-vis spectra of **3Aq**, **3Br** and **3Cl** in aqueous solution. {pH = 6.1; ℓ = 1 cm; T = 25.0 °C}

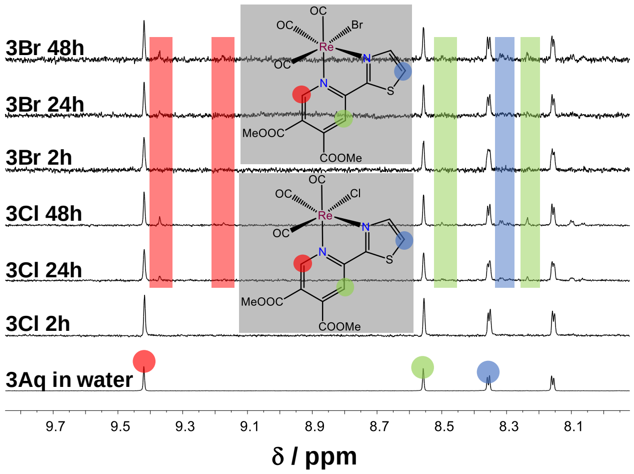

Figure S16. ^{1}H NMR spectra of **3Cl** and **3Br** in water followed in time. Spectrum of **3Aq** is also shown for comparison. The rectangles with coloured background denote to a new peak set, most likely due to the appearance of the species in which the ester bound is partly hydrolysed. {$c_{complex}$ = 70 μM (**3Cl**) or 40 μM (**3Br**); pH = 6.1; 10% (v/v) D_2O/H_2O; T = 25.0 °C}

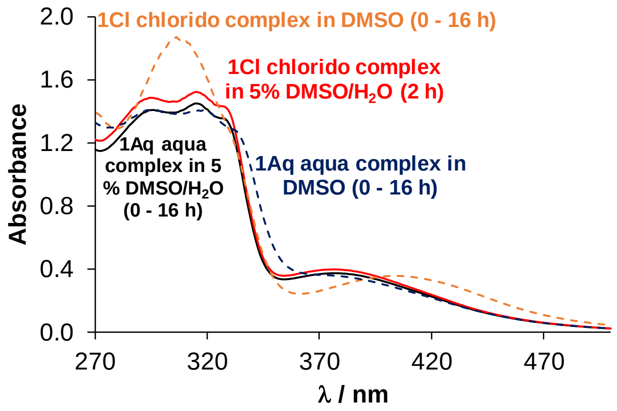

Figure S17. UV-vis spectra of aqua **1Aq** and chlorido **1Cl** complex in DMSO (dashed lines) and 5% (*v/v*) DMSO/H_2O (pH ~ 6.2) (solid lines). {$c_{complex}$= 50 μM; ℓ = 1 cm; T = 25.0 °C}

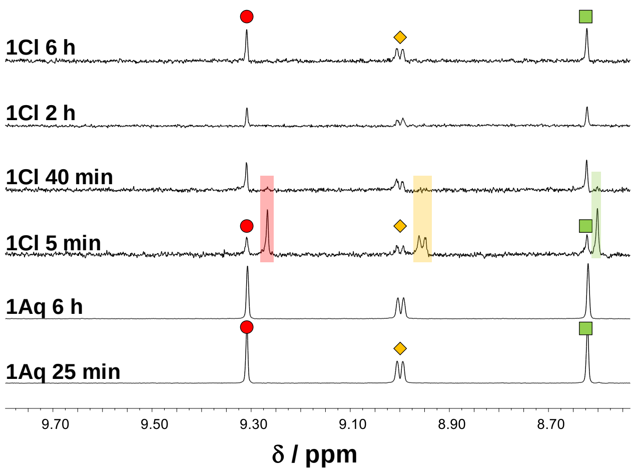

Figure S18. ^{1}H NMR spectra of **1Aq** and **1Cl** in 5% (*v/v*) DMSO-d_6/H_2O followed in time. Rectangles indicate the presence of chlorido complex. {$c_{complex}$ = 500 μM; T = 25.0 °C}

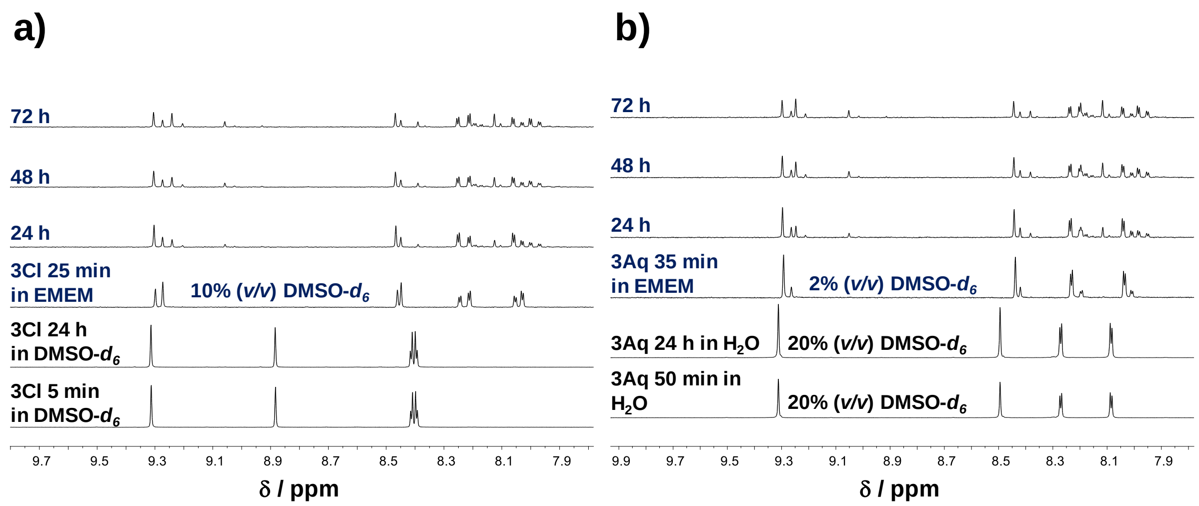

Figure S19. ^{1}H NMR spectra of (a) **3Cl** and (b) **3Aq** DMSO-d_6 and 20% (*v/v*) DMSO-d_6/H_2O medium, respectively, followed in time. Then the solutions were ten-fold-diluted with EMEM and ^{1}H NMR spectra were followed in time. {$c_{complex}$ = 500 μM; T = 25.0 °C}

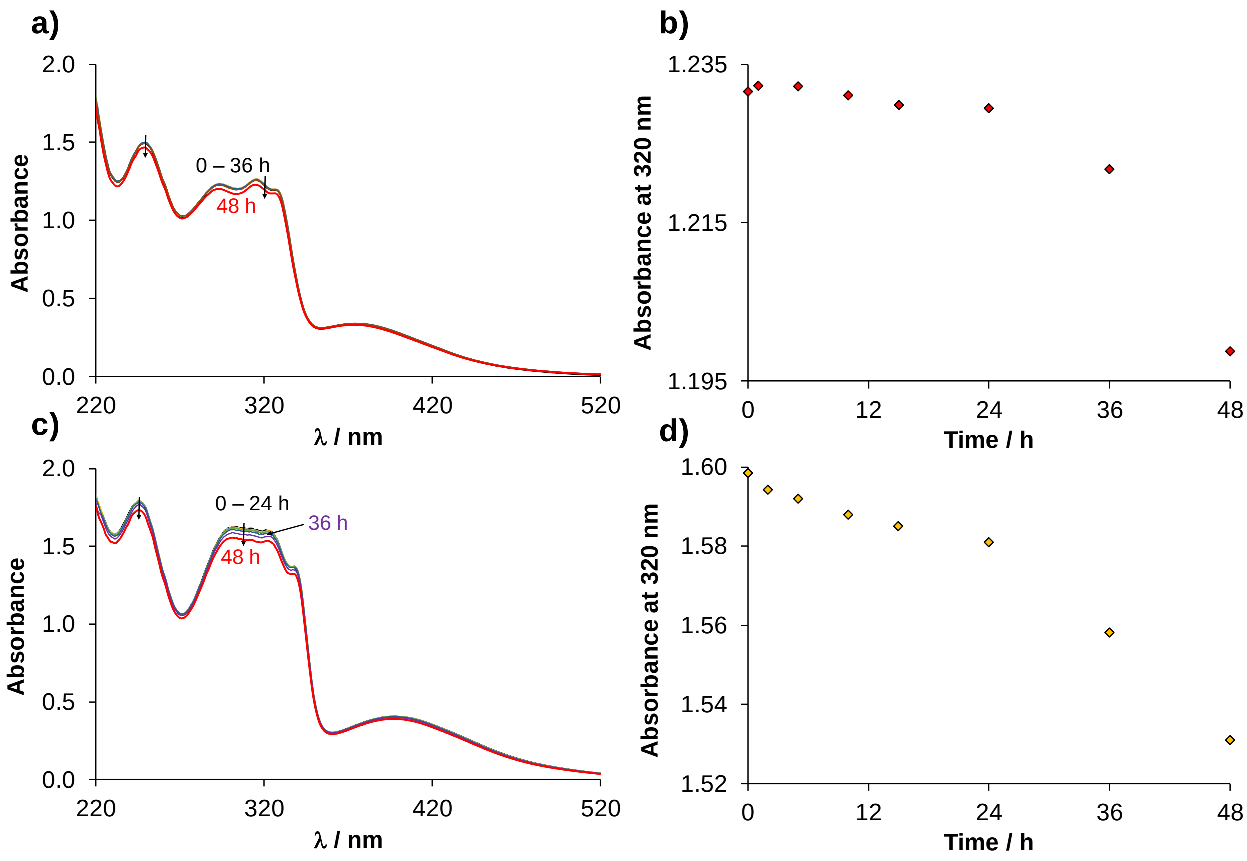

Figure S20. UV-vis spectra of (a) **1Aq** and (c) **2Aq** in water followed over time. Absorbance values at 320 nm (◊) plotted against time (b, d) for the same complexes, respectively. {$c_{complex}$= 100 μM; pH ~6.2; ℓ = 1 cm; T = 25.0 °C}

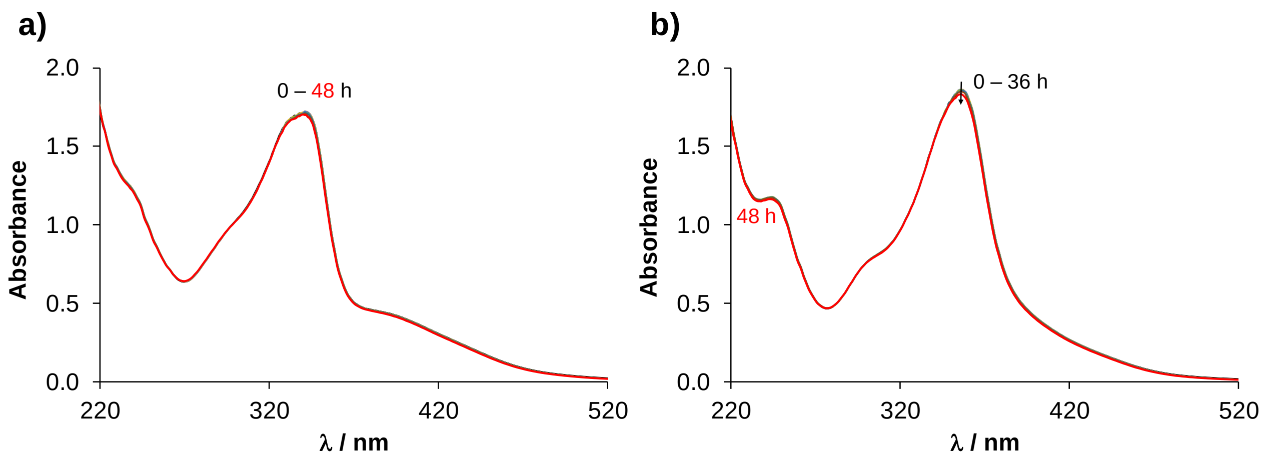

Figure S21. UV-vis spectra of (a) **3Aq** and (b) **4Aq** in water followed in time. {$c_{complex}$= 100 µM; pH ~6.2; ℓ = 1 cm; T = 25.0 °C}

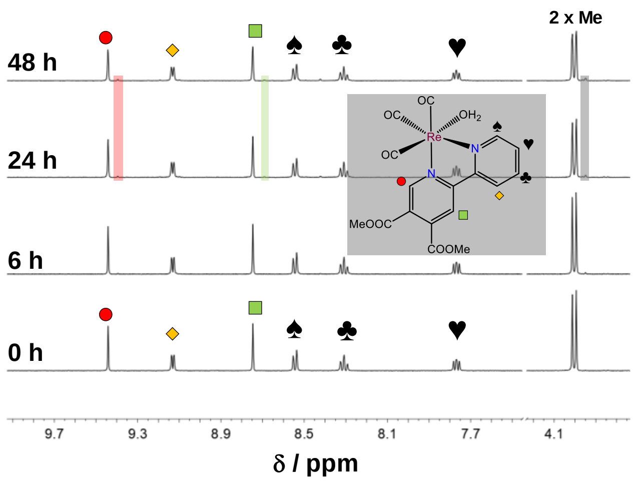

Figure S22. ^{1}H NMR spectra of **1Aq** in water followed over time. Rectangles with coloured background show the appearance of a new set of peaks. {$c_{complex}$= 500 µM; pH = 6.8; 10% (*v/v*) D_2O/H_2O; T = 25.0 °C}

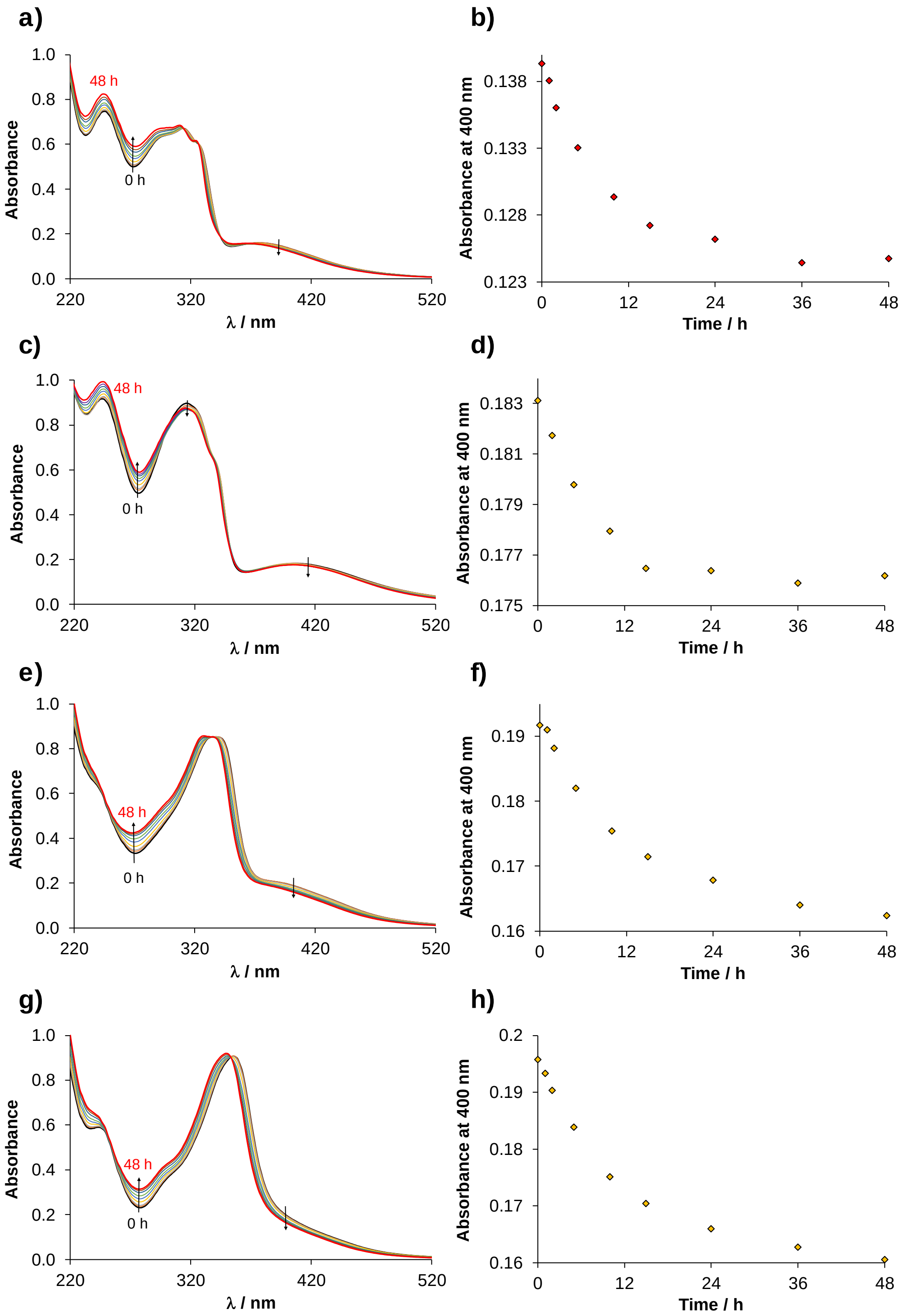

Figure S23. UV-vis spectra of (a) **1Aq**, (c) **2Aq**, (e) **3Aq** and (g) **4Aq** in phosphate buffer (20 mM, pH = 7.4) followed over time. Absorbance values at 400 nm (◊) plotted against time (b, d, f, h) for the same complexes, respectively. {$c_{complex}$ = 50 µM; pH = 7.4; ℓ = 1 cm; T = 25.0 °C}

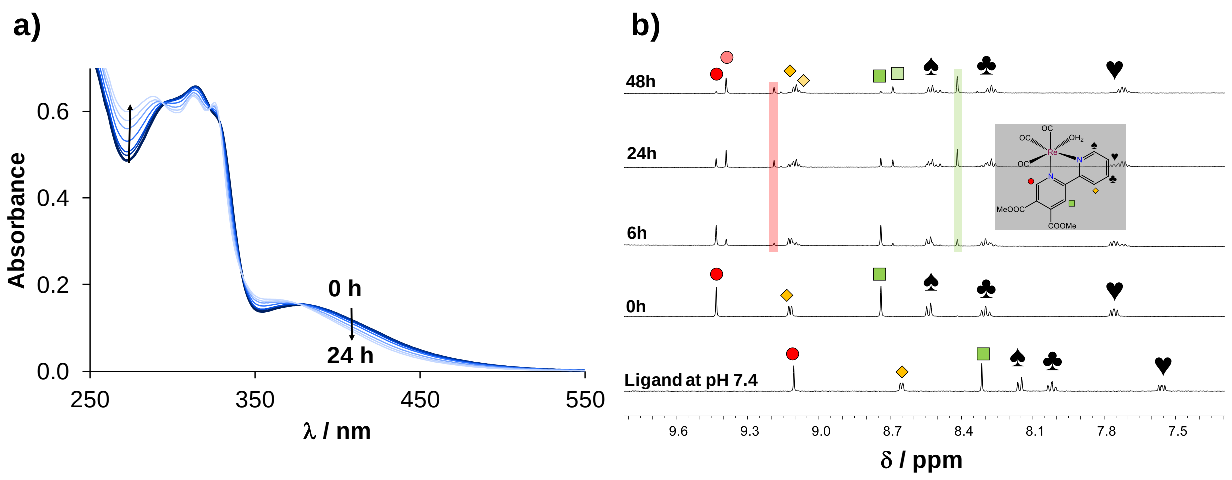

Figure S24. (a) UV-vis and (b) ^{1}H NMR spectra in the low-field region of **1Aq** in HEPES buffer followed in time. The ^{1}H NMR spectrum of the ligand at pH = 7.4 is also shown, and rectangles with coloured background denote to a new peak set. {$c_{complex}$= 50 µM (UV-vis) or 500 µM (^{1}H NMR); pH = 7.4; ℓ = 1 cm (UV-vis); 10% (*v*/*v*) D_2O/H_2O (^{1}H NMR); *T* = 25.0 °C}

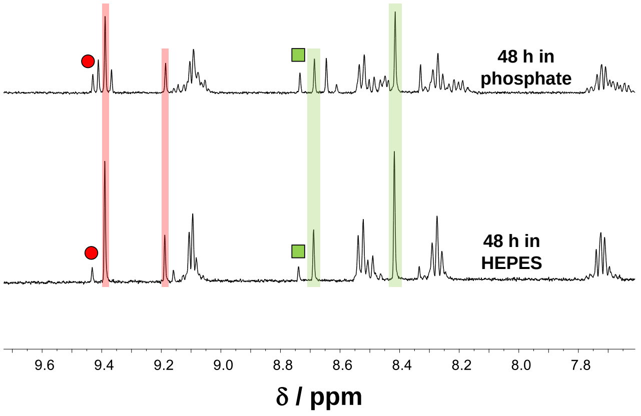

Figure S25. ^{1}H NMR spectra in the low-field region of **1Aq** at pH = 7.4 after 48 h, using either phosphate (20 mM) or HEPES (10 mM) buffer. Rectangles indicate the appearance of hydrolysed products. {$c_{complex}$= 500 µM; pH = 7.4; 10% (*v*/*v*) D_2O/H_2O; *T* = 25.0 °C}

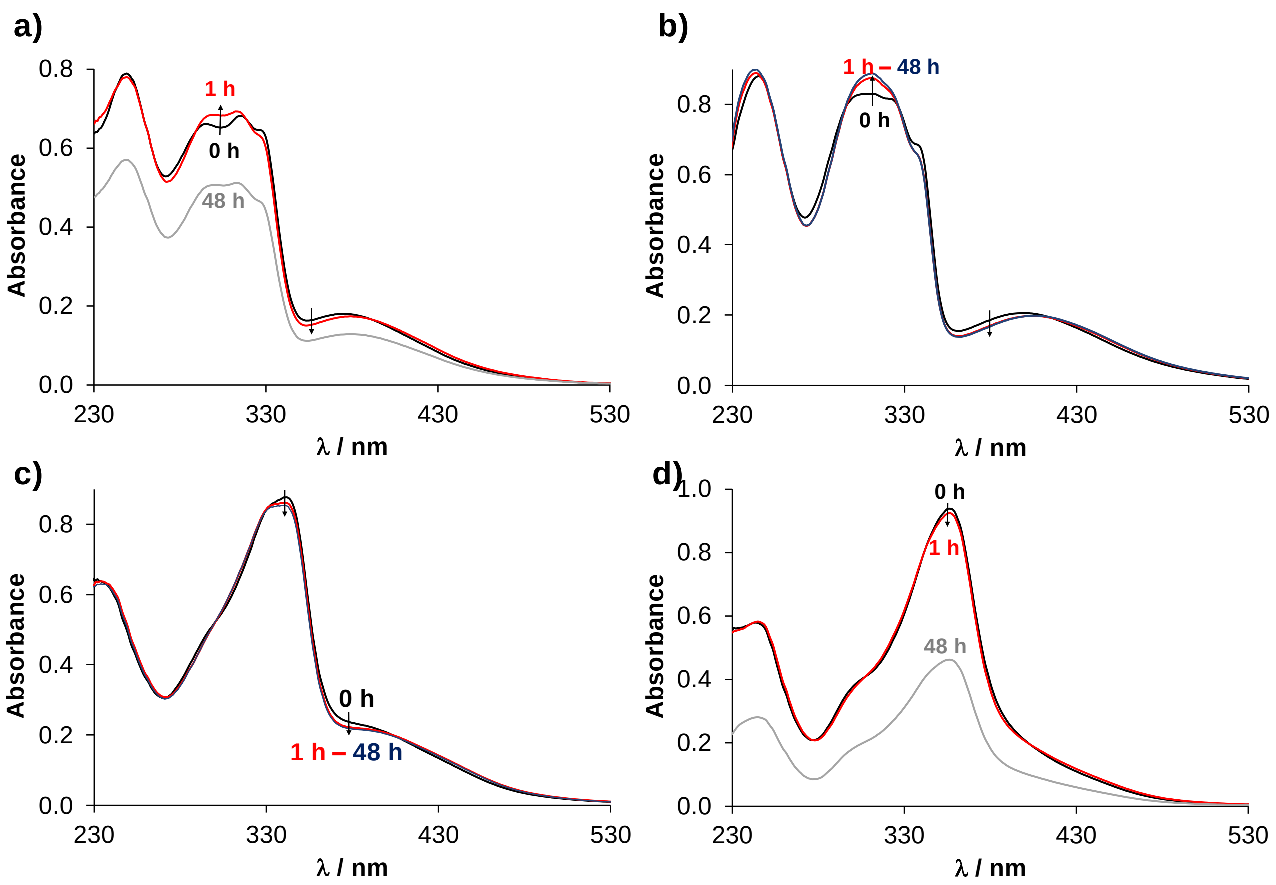

Figure S26. UV-vis spectra of (a) **1Aq**, (b) **2Aq**, (c) **3Aq** and (d) **4Aq** in EMEM at 0, 1 h and 48 h. {$c_{complex}$= 50 µM; ℓ = 1 cm; T = 25.0 °C}

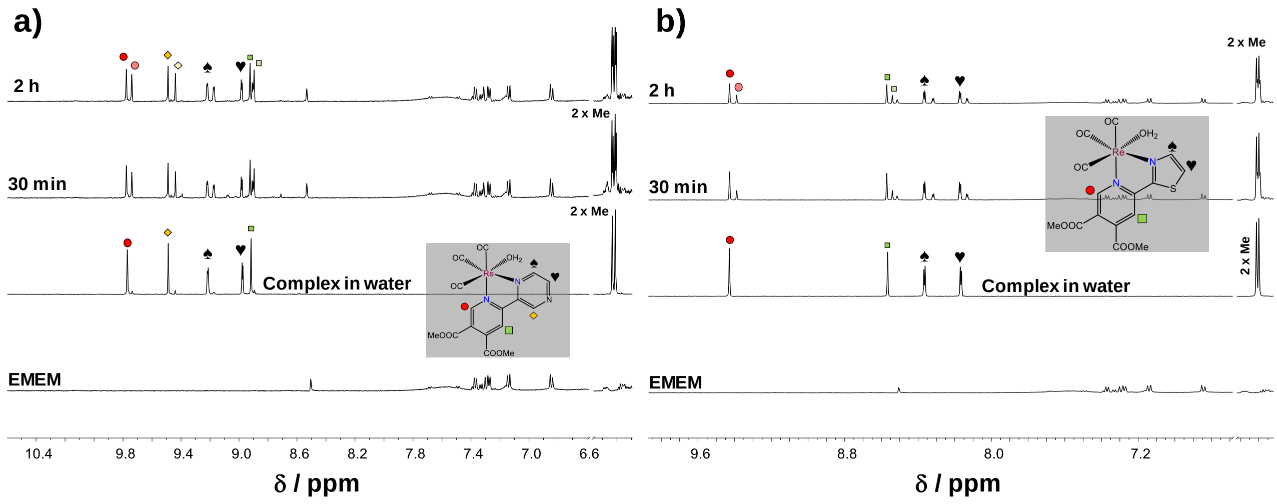

Figure S27. ^{1}H NMR spectra of (a) **2Aq** and (b) **3Aq** in EMEM after 30 min and 2 h. ^{1}H NMR spectra of EMEM and the original complex in water are also shown. {$c_{complex}$= 500 µM; pH = 7.4; 10% (v/v) D_2O/H_2O; T = 25.0 °C}

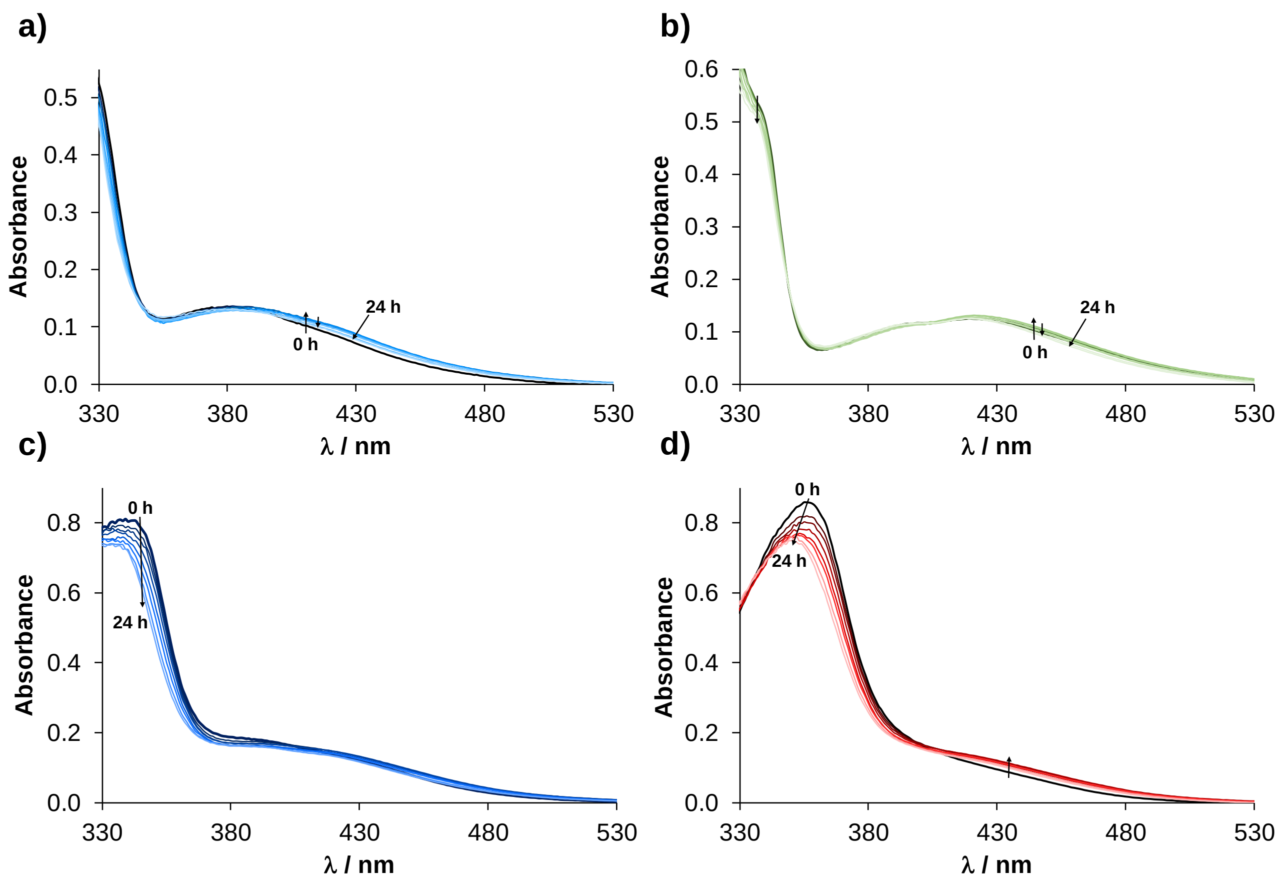

Figure S28. UV-vis spectra of (a) **1Aq**, (b) **2Aq**, (c) **3Aq** and (d) **4Aq** in blood serum followed over time. {$c_{complex}$ = 50 µM; 4-fold diluted blood serum with PBS' buffer (pH = 7.4); ℓ = 1 cm; T = 25.0 °C}

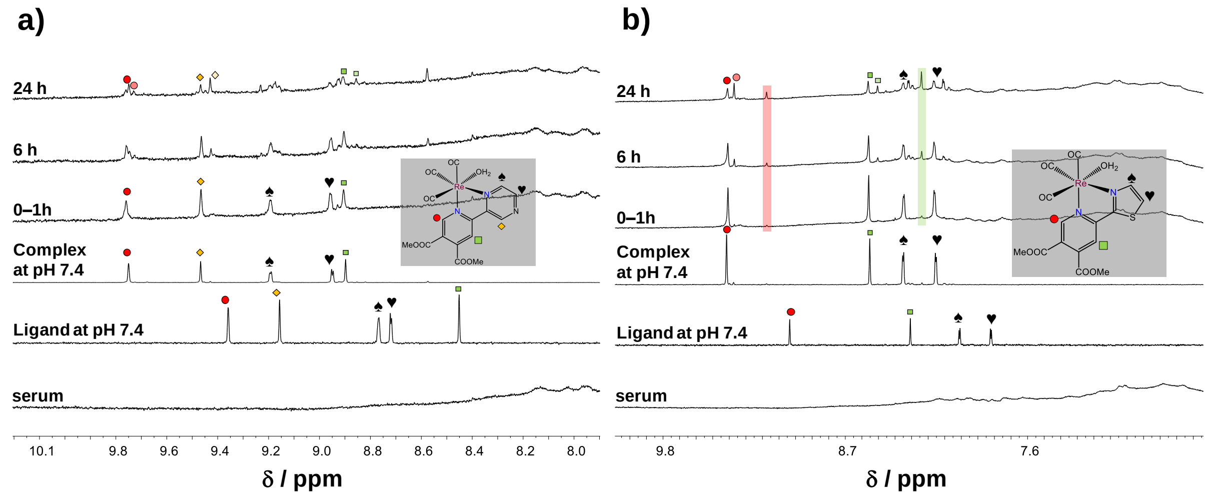

Figure S29. ^{1}H NMR spectra of (a) **2Aq** and (b) **3Aq** in blood serum followed over time. ^{1}H NMR spectra of serum, ligand and complex at pH 7.4 are also shown. Rectangles with coloured background show a new set of peaks. {$c_{complex}$ = 500 µM; 4-fold diluted blood serum with PBS' buffer (pH = 7.4); 10% (v/v) D_2O/H_2O; T = 25.0 °C}

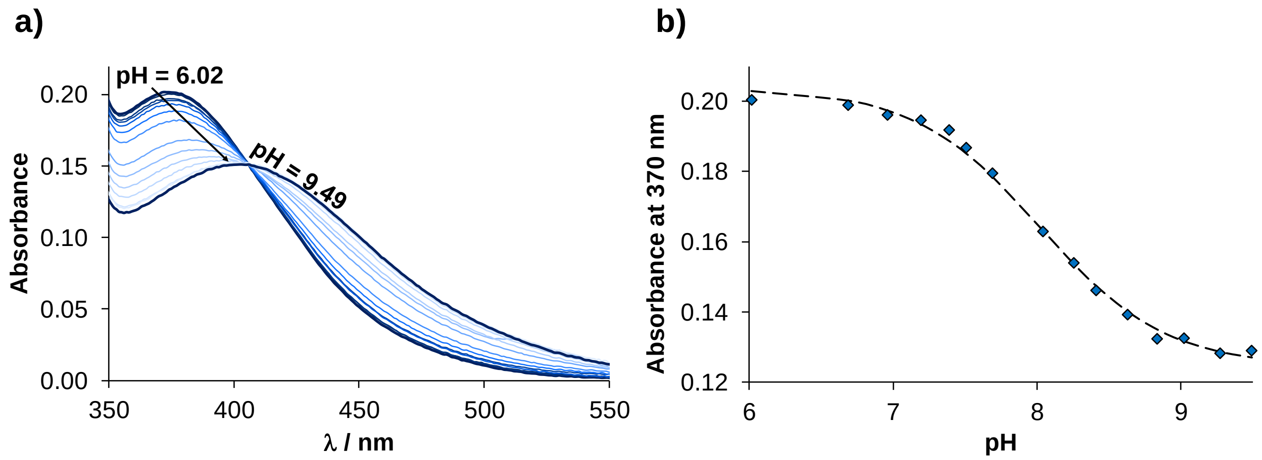

Figure S30. (a) UV-vis spectra of aqua complex **1Aq** in water at increasing pH values (6.02 → 9.49) and (b) absorbance values at 370 nm (♦) plotted against pH along with the fitted curve (dashed line). {$c_{complex}$= 55 µM; I = 0.1 M KNO_3; ℓ = 1 cm; T = 25.0 °C}

Table S6. Ratio (%) of the aqua and hydroxido (deprotonated form) complexes (**1–4Aq**) at pH = 7.4 based on the pK_a (H_2O) values of the complexes determined by UV-vis spectrophotometric titrations (Table 2). {I = 0.1 M KNO_3; T = 25.0 °C}

Complex	Aqua complex (%)	Hydroxido complex (%)
**1Aq**	82	18
**2Aq**	70	30
**3Aq**	81	19
**4Aq**	81	19

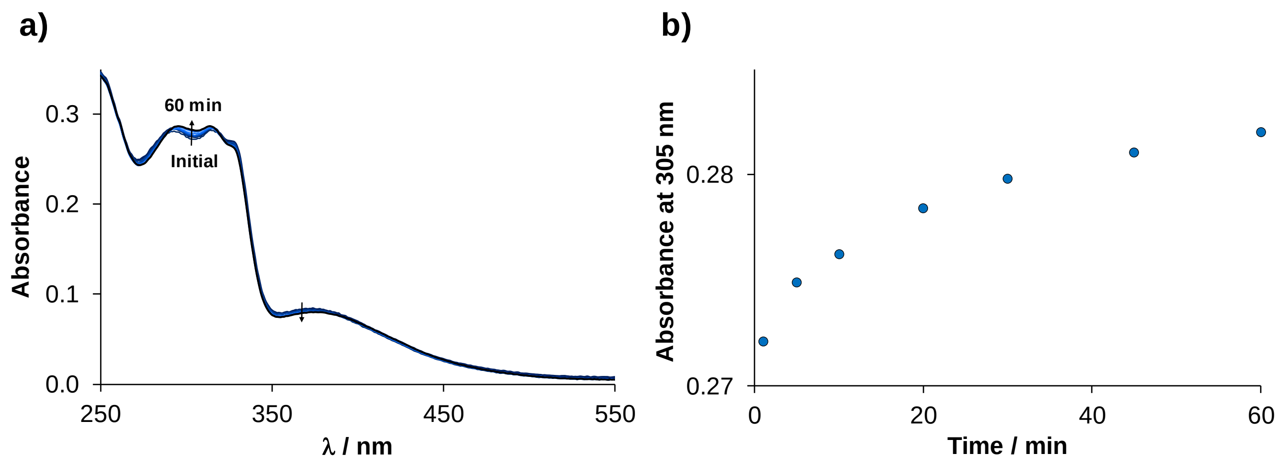

Figure S31. UV-vis spectra of (a) **1Aq** in the presence of chloride ions followed over time. Absorbance values at 305 nm (●) plotted against time (b) for the same complex. {c_{complex} = 25 μM; c_{Cl^-} = 50 mM; pH = 6.1; ℓ = 1 cm; T = 25.0 °C}

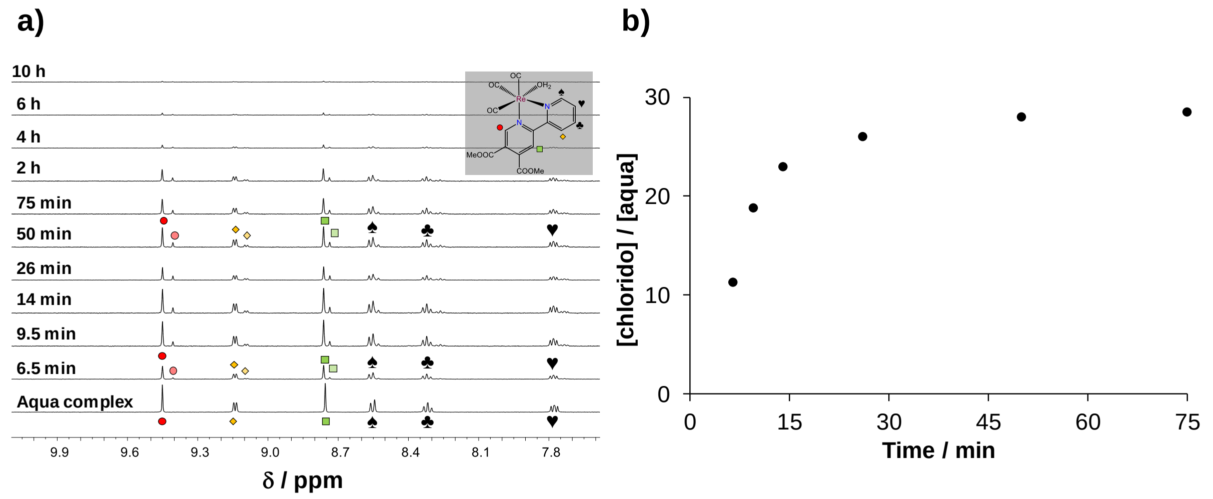

Figure S32. (a) ^{1}H NMR spectra of **1Aq** in the presence of chloride ions followed over time. (6.5 min – 10 h). (b) Ratio of the concentration of the chlorido and aqua complexes plotted against time. (The ratio was obtained on the basis of the integration of the corresponding peaks of the chlorido and aqua complexes). {c_{complex}= 500 μM; c_{Cl^-}= 100 mM; pH = 6.0; 10% (v/v) D_2O/H_2O; T = 25.0 °C}

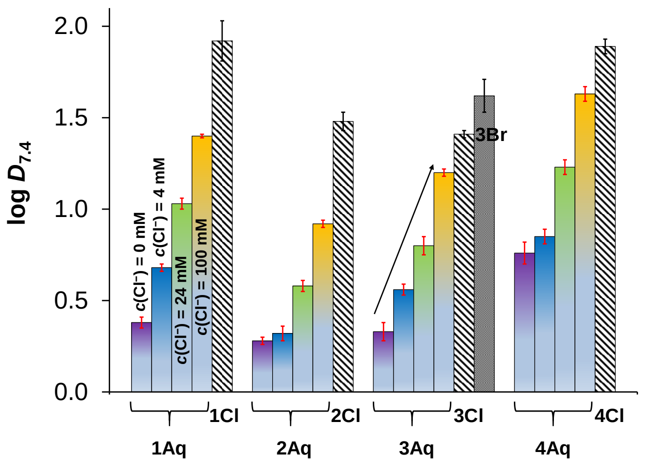

Figure S33. Lipophilicity of the studied complexes expressed as log$D_{7.4}$ measured via *n*-octanol/buffered aqueous solution partitioning at pH = 7.4 (HEPES buffer). For the aqua complexes measurements were done in the absence (0 mM) and in the presence of chloride ions (4, 24 and 100 mM, relevant to the different biofluids). Log$D_{7.4}$ values for halido complexes obtained at 0.1 M KCl and the compounds were previously dissolved in the saturated *n*-octanol phase. {$c_{complex}$ = 50 – 100 µM; incubation time: 4 h; T = 25.0 °C}

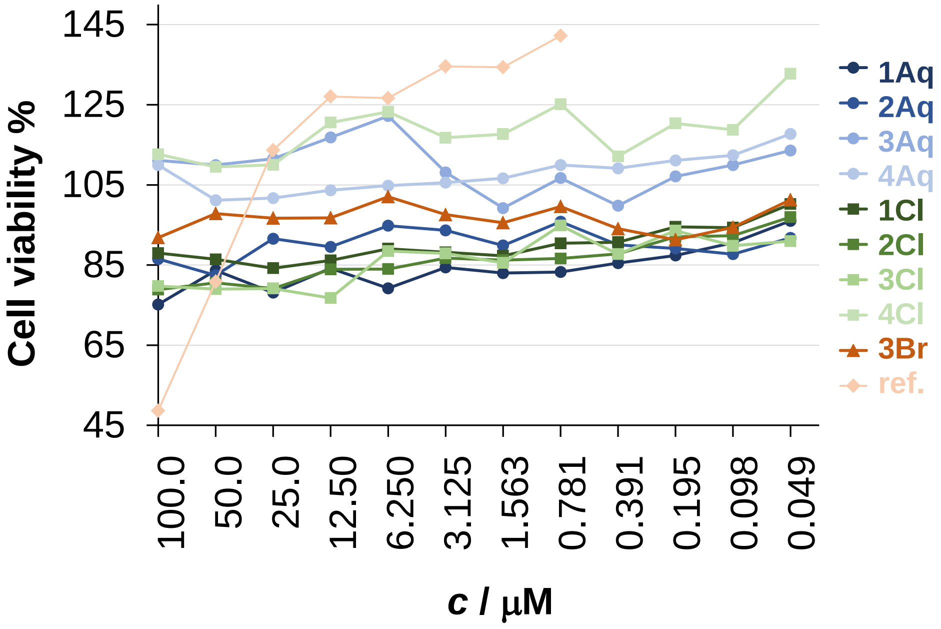

Figure S34. Cytotoxicity of the complexes against Vero cells: no significant toxicity at any of the applied concentrations. 50 µM of complex **1Aq** was found as maximum non-toxic concentration, whereas 100 µM for all other complexes. As a comparison, the reference complex *fac*-[Re(CO)$_3$(bpy)(Cl)] (ref.) was also involved. {Incubation time: 24 h.}

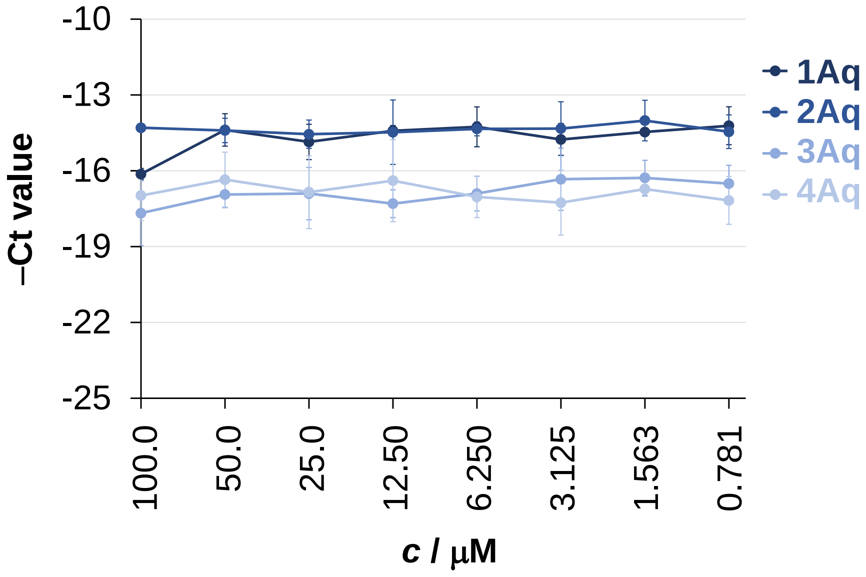

Figure S35. Antiviral effect of complexes **1–4Aq** against HSV-2 at different concentrations. The HSV-2 DNA concentration was measured by direct qPCR. Data represent the average −Ct values ± standard deviations. {Treatment of the Vero cells: 24 h; n = 3}

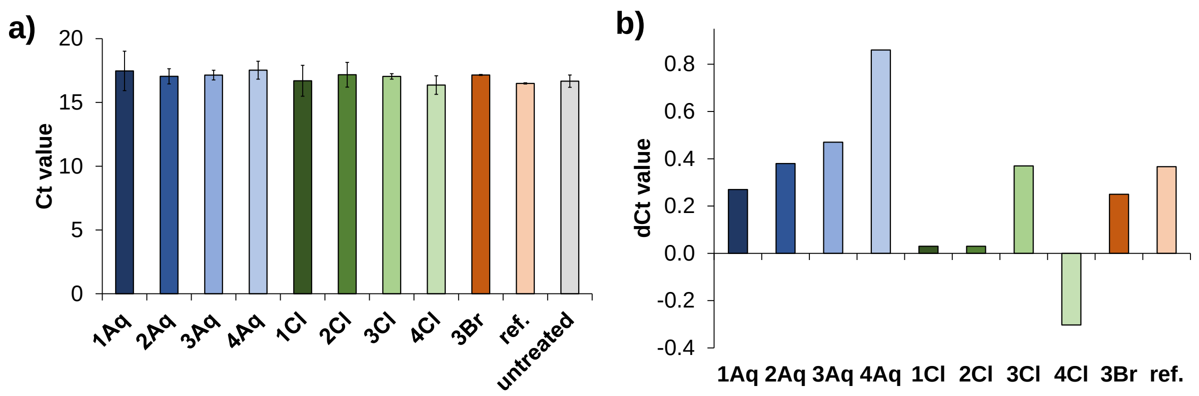

Figure S36. (a) Ct and (b) dCt values when the direct impact of the complexes on the DNA polymerase of qPCR was monitored. Lysates from cells that were treated with the complexes, both infected and uninfected, were mixed in a 1:1 ratio, and the resulting Ct levels were similar to those obtained when lysates from HSV-2 infected cells and uninfected cells were mixed in the same ratio, thus the Ct levels remained consistent regardless of the treatment. As a comparison, the reference complex *fac*-[Re(CO)$_3$(bpy)(Cl)] (ref.) was also involved.
