# Supplementary material for: Structural and Solution Speciation Studies on fac-Tricarbonylrhenium(I) Complexes of 2,2′-Bipyridine Analogues
Source: ACS Omega. 2024 Oct 26;9(44):44601–15. doi: 10.1021/acsomega.4c07117 (PMC11541514; doi:10.1021/acsomega.4c07117)

checkCIF/PLATON report

Structure factors have been supplied for datablock(s) moc177, moc179, moc331, mod12_auto, mod25_auto, mod26_auto, mod27_auto, mod28_auto

THIS REPORT IS FOR GUIDANCE ONLY. IF USED AS PART OF A REVIEW PROCEDURE FOR PUBLICATION, IT SHOULD NOT REPLACE THE EXPERTISE OF AN EXPERIENCED CRYSTALLOGRAPHIC REFEREE.

No syntax errors found.   CIF dictionary   Interpreting this report

Datablock: mod25_auto

```
Bond precision:   C-C = 0.0077 A          Wavelength=0.71073

Cell:           a=12.6956(3)       b=12.9333(3)      c=13.6408(4)
                alpha=64.949(3)    beta=66.006(3)    gamma=89.370(2)
Temperature:    150 K

                 Calculated                   Reported
Volume           1818.24(11)                  1818.24(10)
Space group      P -1                         P -1
Hall group       -P 1                         -P 1
Moiety formula   C16 H11 Cl N3 O7 Re          2(C16 H11 Cl N3 O7 Re)
Sum formula      C16 H11 Cl N3 O7 Re          C32 H22 Cl2 N6 O14 Re2
Mr               578.94                       1157.85
Dx,g cm-3        2.115                        2.115
Z                4                            2
Mu (mm-1)        6.876                        6.876
F000             1104.0                       1104.0
F000'            1101.18
h,k,lmax         16,16,17                     16,16,17
Nref             8348                         8342
Tmin,Tmax        0.508,0.709                  0.559,1.000
Tmin'            0.498

Correction method= # Reported T Limits: Tmin=0.559 Tmax=1.000
AbsCorr = MULTI-SCAN

Data completeness= 0.999          Theta(max)= 27.479

                                                     wR2(reflections)=
R(reflections)= 0.0299( 7000)                        0.0725( 8342)

S = 1.052                   Npar= 509
```

The following ALERTS were generated. Each ALERT has the format
test-name_ALERT_alert-type_alert-level.
Click on the hyperlinks for more details of the test.

Alert level B

```
PLAT971_ALERT_2_B Check Calcd Resid. Dens.  0.85Ang From Re1           3.47 eA-3
```

Alert level C

```
PLAT042_ALERT_1_C Calc. and Reported MoietyFormula Strings  Differ     Please Check
              Calc: C16 H11 Cl N3 O7 Re
              Rep.: 2(C16 H11 Cl N3 O7 Re)
PLAT094_ALERT_2_C Ratio of Maximum / Minimum Residual Density ....       2.98 Report
PLAT220_ALERT_2_C NonSolvent   Resd 2  C   Ueq(max)/Ueq(min) Range        3.7 Ratio
PLAT250_ALERT_2_C Large U3/U1 Ratio for <U(i,j)> Tensor(Resd    1)        2.1 Note
PLAT910_ALERT_3_C Missing # of FCF Reflection(s) Below Theta(Min).          7 Note
                1  0  0,  -1  1  0,   0  1  0,   0  0  1,   1  0  1,   0  1  1,
                1  1  1,
PLAT971_ALERT_2_C Check Calcd Resid. Dens.  0.90Ang From Re1*           1.87 eA-3
PLAT971_ALERT_2_C Check Calcd Resid. Dens.  0.85Ang From Re1*           1.58 eA-3
PLAT975_ALERT_2_C Check Calcd Resid. Dens.  1.03Ang From O15*    .      0.62 eA-3
PLAT975_ALERT_2_C Check Calcd Resid. Dens.  0.99Ang From O15*    .      0.57 eA-3
```

Alert level G

```
PLAT045_ALERT_1_G Calculated and Reported Z Differ by a Factor ...          2 Check
PLAT720_ALERT_4_G Number of Unusual/Non-Standard Labels .........          33 Note
              Re1*    Cl1*    N1*     N7*     O19*    O2*A    O2*B    O2*C
              C1*B    O14*    C1*A    N10*    C8*     C2*     O15*    O18*
              C9*     H9*     C5*     C6*     H6*     C3*     H3*     C1*C
              C11*    H11*    C4*     C12*    H12*    C17*    C13*    C20*
              C16*
PLAT941_ALERT_3_G Average HKL Measurement Multiplicity ..........         4.1 Low
PLAT969_ALERT_5_G The 'Henn et al.' R-Factor-gap value ..........       2.468 Note
              Predicted wR2: Based on SigI**2  2.94 or SHELX Weight  6.89
PLAT978_ALERT_2_G Number C-C Bonds with Positive Residual Density.          0 Info
```

0 **ALERT level A** = Most likely a serious problem - resolve or explain
1 **ALERT level B** = A potentially serious problem, consider carefully
9 **ALERT level C** = Check. Ensure it is not caused by an omission or oversight
5 **ALERT level G** = General information/check it is not something unexpected

2 ALERT type 1 CIF construction/syntax error, inconsistent or missing data
9 ALERT type 2 Indicator that the structure model may be wrong or deficient
2 ALERT type 3 Indicator that the structure quality may be low
1 ALERT type 4 Improvement, methodology, query or suggestion
1 ALERT type 5 Informative message, check

Datablock: mod12_auto

Bond precision: C-C = 0.0082 A Wavelength=0.71073

Cell:	a=11.0635(3)	b=8.0622(2)	c=24.6138(6)
	alpha=90	beta=101.704(2)	gamma=90
Temperature:	150 K		

	Calculated	Reported
Volume	2149.81(10)	2149.81(10)
Space group	P 21/n	P 1 21/n 1
Hall group	-P 2yn	-P 2yn
Moiety formula	C15 H10 Cl N2 O7 Re S	C15 H10 Cl N2 O7 Re S
Sum formula	C15 H10 Cl N2 O7 Re S	C15 H10 Cl N2 O7 Re S
Mr	583.97	583.96
Dx,g cm-3	1.804	1.804
Z	4	4
Mu (mm-1)	5.909	5.909
F000	1112.0	1112.0
F000’	1109.67	
h,k,lmax	14,10,31	14,10,31
Nref	4941	4936
Tmin,Tmax	0.838,0.863	0.479,1.000
Tmin’	0.307	

Correction method= # Reported T Limits: Tmin=0.479 Tmax=1.000
AbsCorr = MULTI-SCAN

Data completeness= 0.999 Theta(max)= 27.481

R(reflections)= 0.0368(4015) wR2(reflections)= 0.0865(4936)

S = 1.107 Npar= 246

The following ALERTS were generated. Each ALERT has the format
test-name_ALERT_alert-type_alert-level.
Click on the hyperlinks for more details of the test.

Alert level A

PLAT601_ALERT_2_A Unit Cell Contains Solvent Accessible VOIDS of . 223 Ang**3

Alert level B

PLAT971_ALERT_2_B Check Calcd Resid. Dens. 2.65Ang From C15 2.54 eA-3

Alert level C

PLAT094_ALERT_2_C Ratio of Maximum / Minimum Residual Density .... 2.46 Report

```
PLAT213_ALERT_2_C Atom C15            has ADP max/min Ratio .....        3.5 prolat
PLAT220_ALERT_2_C NonSolvent   Resd 1  C   Ueq(max)/Ueq(min) Range       4.8 Ratio
PLAT222_ALERT_3_C NonSolvent Resd 1  H   Uiso(max)/Uiso(min) Range       4.9 Ratio
PLAT342_ALERT_3_C Low Bond Precision on  C-C Bonds ..............    0.00825 Ang.
PLAT906_ALERT_3_C Large K Value in the Analysis of Variance ......      3.227 Check
PLAT911_ALERT_3_C Missing FCF Refl Between Thmin & STh/L=    0.600          2 Report
                3  2  1,   0  1  4,
PLAT971_ALERT_2_C Check Calcd Resid. Dens.  2.65Ang From C15            2.06 eA-3
PLAT971_ALERT_2_C Check Calcd Resid. Dens.  2.65Ang From C15            1.84 eA-3
PLAT971_ALERT_2_C Check Calcd Resid. Dens.  2.65Ang From C15            1.83 eA-3
```

Alert level G

```
PLAT083_ALERT_2_G SHELXL Second Parameter in WGHT  Unusually Large       7.79 Why ?
PLAT432_ALERT_2_G Short Inter X...Y Contact  O13      ..C16      .       2.94 Ang.
                                         1/2-x,1/2+y,1/2-z  =      2_555 Check
PLAT910_ALERT_3_G Missing # of FCF Reflection(s) Below Theta(Min).         3 Note
               -1  0  1,   1  0  1,   0  0  2,
PLAT933_ALERT_2_G Number of HKL-OMIT Records in Embedded .res File         2 Note
                0  1  4,   3  2  1,
PLAT969_ALERT_5_G The 'Henn et al.' R-Factor-gap value ..........      3.093 Note
              Predicted wR2: Based on SigI**2  2.80 or SHELX Weight  7.82
PLAT978_ALERT_2_G Number C-C Bonds with Positive Residual Density.         0 Info
```

---

1 **ALERT level A** = Most likely a serious problem - resolve or explain
1 **ALERT level B** = A potentially serious problem, consider carefully
10 **ALERT level C** = Check. Ensure it is not caused by an omission or oversight
6 **ALERT level G** = General information/check it is not something unexpected

0 ALERT type 1 CIF construction/syntax error, inconsistent or missing data
12 ALERT type 2 Indicator that the structure model may be wrong or deficient
5 ALERT type 3 Indicator that the structure quality may be low
0 ALERT type 4 Improvement, methodology, query or suggestion
1 ALERT type 5 Informative message, check

Datablock: mod28_auto

```
Bond precision:   C-C = 0.0066 A          Wavelength=0.71073

Cell:           a=9.8309(4)        b=10.0620(3)       c=10.3204(4)
                alpha=89.429(3)    beta=71.172(4)     gamma=78.930(3)
Temperature:    150 K
```

	Calculated	Reported
Volume	946.79(7)	946.79(6)
Space group	P -1	P -1
Hall group	-P 1	-P 1
Moiety formula	C16 H12 Cl N2 O7 Re S	C16 H12 Cl N2 O7 Re S
Sum formula	C16 H12 Cl N2 O7 Re S	C16 H12 Cl N2 O7 Re S
Mr	598.00	597.99
Dx,g cm-3	2.098	2.098
Z	2	2
Mu (mm-1)	6.711	6.711
F000	572.0	572.0
F000'	570.84	
h,k,lmax	12,13,13	12,13,13
Nref	4345	4341
Tmin,Tmax	0.204,0.511	0.637,1.000
Tmin'	0.171	

Correction method= # Reported T Limits: Tmin=0.637 Tmax=1.000
AbsCorr = MULTI-SCAN

Data completeness= 0.999 Theta(max)= 27.484

R(reflections)= 0.0319(3896) wR2(reflections)= 0.0686(4341)

S = 1.025 Npar= 250

The following ALERTS were generated. Each ALERT has the format
test-name_ALERT_alert-type_alert-level.
Click on the hyperlinks for more details of the test.

Alert level C

```
PLAT250_ALERT_2_C Large U3/U1 Ratio for <U(i,j)> Tensor(Resd    1)        2.2 Note
PLAT971_ALERT_2_C Check Calcd Resid. Dens.  1.09Ang From Re1             2.21 eA-3
PLAT971_ALERT_2_C Check Calcd Resid. Dens.  0.92Ang From Re1             2.02 eA-3
PLAT972_ALERT_2_C Check Calcd Resid. Dens.  0.72Ang From Re1            -1.55 eA-3
```

Alert level G

```
PLAT171_ALERT_4_G The CIF-Embedded .res File Contains EADP Records          1 Report
PLAT230_ALERT_2_G Hirshfeld Test Diff for    O2A      --C1A      .       10.2 s.u.
PLAT230_ALERT_2_G Hirshfeld Test Diff for    O2C      --C1C      .        5.7 s.u.
PLAT232_ALERT_2_G Hirshfeld Test Diff (M-X)  Re1      --Cl1      .        6.3 s.u.
PLAT232_ALERT_2_G Hirshfeld Test Diff (M-X)  Re1      --C1C      .        5.7 s.u.
PLAT910_ALERT_3_G Missing # of FCF Reflection(s) Below Theta(Min).          4 Note
              1  0  0,   0  1  0,   0  0  1,   1  0  1,
PLAT941_ALERT_3_G Average HKL Measurement Multiplicity ..........         2.0 Low
PLAT969_ALERT_5_G The 'Henn et al.' R-Factor-gap value ..........       1.415 Note
           Predicted wR2: Based on SigI**2  4.84 or SHELX Weight  6.69
```

PLAT978_ALERT_2_G Number C-C Bonds with Positive Residual Density. 1 Info

---

0 **ALERT level A** = Most likely a serious problem - resolve or explain
0 **ALERT level B** = A potentially serious problem, consider carefully
4 **ALERT level C** = Check. Ensure it is not caused by an omission or oversight
9 **ALERT level G** = General information/check it is not something unexpected

0 ALERT type 1 CIF construction/syntax error, inconsistent or missing data
9 ALERT type 2 Indicator that the structure model may be wrong or deficient
2 ALERT type 3 Indicator that the structure quality may be low
1 ALERT type 4 Improvement, methodology, query or suggestion
1 ALERT type 5 Informative message, check

Datablock: moc177

Bond precision: C-C = 0.0072 A Wavelength=0.71073

Cell:	a=12.3403(4)	b=8.1416(2)	c=25.0932(7)
	alpha=90	beta=102.897(3)	gamma=90
Temperature:	150 K		

	Calculated	Reported
Volume	2457.51(12)	2457.51(12)
Space group	P 21/n	P 1 21/n 1
Hall group	-P 2yn	-P 2yn
Moiety formula	C15 H10 Br N2 O7 Re S, C7 H8	C15 H10 Br N2 O7 Re S, C7 H8
Sum formula	C22 H18 Br N2 O7 Re S	C22 H18 Br N2 O7 Re S
Mr	720.55	720.55
Dx,g cm-3	1.947	1.948
Z	4	4
Mu (mm-1)	6.700	6.700
F000	1384.0	1384.0
F000'	1380.11	
h,k,lmax	16,10,32	16,10,32
Nref	5645	5585
Tmin,Tmax	0.676,0.715	0.516,1.000
Tmin'	0.259	

Correction method= # Reported T Limits: Tmin=0.516 Tmax=1.000
AbsCorr = MULTI-SCAN

Data completeness= 0.989 Theta(max)= 27.483

R(reflections)= 0.0334(4315) wR2(reflections)= 0.0605(5585)

S = 0.990 Npar= 292

The following ALERTS were generated. Each ALERT has the format
test-name_ALERT_alert-type_alert-level.
Click on the hyperlinks for more details of the test.

Alert level C

```
PLAT431_ALERT_2_C Short Inter HL..A Contact  Br1       ..S7       .        3.46 Ang.
                                                 x,-1+y,z  =      1_545 Check
PLAT906_ALERT_3_C Large K Value in the Analysis of Variance ......       3.234 Check
PLAT911_ALERT_3_C Missing FCF Refl Between Thmin & STh/L=    0.600          2 Report
                0  5  3,   0  2  4,
```

Alert level G

```
PLAT171_ALERT_4_G The CIF-Embedded .res File Contains EADP Records          3 Report
PLAT232_ALERT_2_G Hirshfeld Test Diff (M-X)  Re1       --Br1      .       14.5 s.u.
PLAT232_ALERT_2_G Hirshfeld Test Diff (M-X)  Re1       --C1A      .        8.5 s.u.
PLAT432_ALERT_2_G Short Inter X...Y Contact  O13       ..C16      .       2.95 Ang.
                                        3/2-x,1/2+y,3/2-z  =      2_656 Check
PLAT720_ALERT_4_G Number of Unusual/Non-Standard Labels .........          3 Note
              H7TA    H7TB    H7TC
PLAT910_ALERT_3_G Missing # of FCF Reflection(s) Below Theta(Min).          3 Note
               -1  0  1,   1  0  1,   0  0  2,
PLAT912_ALERT_4_G Missing # of FCF Reflections Above STh/L=  0.600         55 Note
PLAT933_ALERT_2_G Number of HKL-OMIT Records in Embedded .res File          2 Note
                0  2  4,   0  5  3,
PLAT969_ALERT_5_G The 'Henn et al.' R-Factor-gap value ..........       1.645 Note
              Predicted wR2: Based on SigI**2  3.68 or SHELX Weight  6.11
PLAT978_ALERT_2_G Number C-C Bonds with Positive Residual Density.          0 Info
```

0 **ALERT level A** = Most likely a serious problem - resolve or explain
0 **ALERT level B** = A potentially serious problem, consider carefully
3 **ALERT level C** = Check. Ensure it is not caused by an omission or oversight
10 **ALERT level G** = General information/check it is not something unexpected

0 ALERT type 1 CIF construction/syntax error, inconsistent or missing data
6 ALERT type 2 Indicator that the structure model may be wrong or deficient
3 ALERT type 3 Indicator that the structure quality may be low
3 ALERT type 4 Improvement, methodology, query or suggestion
1 ALERT type 5 Informative message, check

Datablock: moc179

Bond precision: C-C = 0.0071 A Wavelength=0.71073

Cell:	a=10.0127(3)	b=10.1780(3)	c=10.3325(3)
	alpha=87.887(2)	beta=70.324(3)	gamma=77.293(2)
Temperature:	150 K		

	Calculated	Reported
Volume	966.42(5)	966.42(5)
Space group	P -1	P -1
Hall group	-P 1	-P 1
Moiety formula	C16 H12 Br N2 O7 Re S	C16 H12 Br N2 O7 Re S
Sum formula	C16 H12 Br N2 O7 Re S	C16 H12 Br N2 O7 Re S
Mr	642.45	642.45
Dx,g cm-3	2.208	2.208
Z	2	2
Mu (mm-1)	8.504	8.504
F000	608.0	608.0
F000'	606.06	
h,k,lmax	12,13,13	12,13,13
Nref	4438	4424
Tmin,Tmax	0.090,0.427	0.384,1.000
Tmin'	0.068	

Correction method= # Reported T Limits: Tmin=0.384 Tmax=1.000
AbsCorr = MULTI-SCAN

Data completeness= 0.997 Theta(max)= 27.485

R(reflections)= 0.0311(4050) wR2(reflections)= 0.0748(4424)

S = 1.035 Npar= 250

The following ALERTS were generated. Each ALERT has the format
test-name_ALERT_alert-type_alert-level.
Click on the hyperlinks for more details of the test.

Alert level B

PLAT971_ALERT_2_B Check Calcd Resid. Dens. 0.19Ang From C1A 2.97 eA-3

Alert level C

PLAT250_ALERT_2_C Large U3/U1 Ratio for <U(i,j)> Tensor(Resd 1) 2.5 Note
PLAT971_ALERT_2_C Check Calcd Resid. Dens. 0.75Ang From Re1 2.28 eA-3
PLAT971_ALERT_2_C Check Calcd Resid. Dens. 1.11Ang From Br1 1.54 eA-3
PLAT972_ALERT_2_C Check Calcd Resid. Dens. 0.23Ang From O2A -1.79 eA-3
PLAT972_ALERT_2_C Check Calcd Resid. Dens. 2.20Ang From C7 -1.54 eA-3

Alert level G

```
PLAT171_ALERT_4_G The CIF-Embedded .res File Contains EADP Records          1 Report
PLAT232_ALERT_2_G Hirshfeld Test Diff (M-X)  Re1      --C1A      .       11.7 s.u.
PLAT232_ALERT_2_G Hirshfeld Test Diff (M-X)  Re1      --C1B      .        5.5 s.u.
PLAT910_ALERT_3_G Missing # of FCF Reflection(s) Below Theta(Min).          3 Note
                1  0  0,   0  1  0,   0  0  1,
PLAT912_ALERT_4_G Missing # of FCF Reflections Above STh/L=  0.600         10 Note
PLAT933_ALERT_2_G Number of HKL-OMIT Records in Embedded .res File          1 Note
                3 12  6,
PLAT969_ALERT_5_G The 'Henn et al.' R-Factor-gap value ..........       2.514 Note
              Predicted wR2: Based on SigI**2  2.97 or SHELX Weight  7.23
PLAT978_ALERT_2_G Number C-C Bonds with Positive Residual Density.          1 Info
```

---

0 **ALERT level A** = Most likely a serious problem - resolve or explain
1 **ALERT level B** = A potentially serious problem, consider carefully
5 **ALERT level C** = Check. Ensure it is not caused by an omission or oversight
8 **ALERT level G** = General information/check it is not something unexpected

0 ALERT type 1 CIF construction/syntax error, inconsistent or missing data
10 ALERT type 2 Indicator that the structure model may be wrong or deficient
1 ALERT type 3 Indicator that the structure quality may be low
2 ALERT type 4 Improvement, methodology, query or suggestion
1 ALERT type 5 Informative message, check

Datablock: mod26_auto

Bond precision:	C-C = 0.0048 A		Wavelength=0.71073
Cell:	a=9.4962(2)	b=11.1763(4)	c=11.4027(4)
	alpha=110.491(3)	beta=92.619(2)	gamma=96.465(2)
Temperature:	150 K		

	Calculated	Reported
Volume	1121.70(7)	1121.69(6)
Space group	P -1	P -1
Hall group	-P 1	-P 1
Moiety formula	C17 H14 N2 O8 Re, C H3 O3 S	C17 H14 N2 O8 Re, C H3 O3 S
Sum formula	C18 H17 N2 O11 Re S	C18 H17 N2 O11 Re S
Mr	655.61	655.59
Dx,g cm-3	1.941	1.941
Z	2	2
Mu (mm-1)	5.571	5.571
F000	636.0	636.0
F000'	634.60	
h,k,lmax	12,14,14	12,14,14
Nref	5146	5143
Tmin,Tmax	0.341,0.434	0.703,1.000
Tmin'	0.316	

Correction method= # Reported T Limits: Tmin=0.703 Tmax=1.000
AbsCorr = MULTI-SCAN

Data completeness= 0.999 Theta(max)= 27.483

R(reflections)= 0.0250(4655) wR2(reflections)= 0.0507(5143)

S = 1.050 Npar= 302

The following ALERTS were generated. Each ALERT has the format
test-name_ALERT_alert-type_alert-level.
Click on the hyperlinks for more details of the test.

Alert level C

PLAT244_ALERT_4_C Low 'Solvent' Ueq as Compared to Neighbors of S2MS Check

---

Alert level G

```
PLAT007_ALERT_5_G Number of Unrefined Donor-H Atoms ..............          2 Report
             H1WA  H1WB
PLAT230_ALERT_2_G Hirshfeld Test Diff for    O2B      --C1B      .        6.5 s.u.
PLAT230_ALERT_2_G Hirshfeld Test Diff for    O2C      --C1C      .        6.0 s.u.
PLAT232_ALERT_2_G Hirshfeld Test Diff (M-X)  Re1      --C1A      .        5.6 s.u.
PLAT232_ALERT_2_G Hirshfeld Test Diff (M-X)  Re1      --C1B      .        6.4 s.u.
PLAT232_ALERT_2_G Hirshfeld Test Diff (M-X)  Re1      --C1C      .        6.7 s.u.
PLAT720_ALERT_4_G Number of Unusual/Non-Standard Labels ..........         10 Note
             S2MS    H1WA    H1WB    O3MS    O5MS    O4MS    C1MS    H1MA
             H1MB    H1MC
PLAT910_ALERT_3_G Missing # of FCF Reflection(s) Below Theta(Min).          4 Note
               1  0  0,   0  1  0,   0 -1  1,   0  0  1,
PLAT941_ALERT_3_G Average HKL Measurement Multiplicity ..........         3.4 Low
```

```
PLAT969_ALERT_5_G The 'Henn et al.' R-Factor-gap value ...........      1.420 Note
              Predicted wR2: Based on SigI**2  3.57 or SHELX Weight  4.83
PLAT978_ALERT_2_G Number C-C Bonds with Positive Residual Density.         1 Info
```

```
   0 ALERT level A = Most likely a serious problem - resolve or explain
   0 ALERT level B = A potentially serious problem, consider carefully
   1 ALERT level C = Check. Ensure it is not caused by an omission or oversight
  11 ALERT level G = General information/check it is not something unexpected

   0 ALERT type 1 CIF construction/syntax error, inconsistent or missing data
   6 ALERT type 2 Indicator that the structure model may be wrong or deficient
   2 ALERT type 3 Indicator that the structure quality may be low
   2 ALERT type 4 Improvement, methodology, query or suggestion
   2 ALERT type 5 Informative message, check
```

Datablock: mod27_auto

Bond precision: C-C = 0.0057 A   Wavelength=0.71073

Cell:	a=8.0160(2)	b=11.8334(3)	c=12.7420(3)
	alpha=110.646(2)	beta=94.578(2)	gamma=94.117(2)
Temperature:	150 K		

	Calculated	Reported
Volume	1120.94(5)	1120.94(5)
Space group	P -1	P -1
Hall group	-P 1	-P 1
Moiety formula	C16 H14 N2 O8 Re S, C H3 O3 S	C16 H14 N2 O8 Re S, C H3 O3 S
Sum formula	C17 H17 N2 O11 Re S2	C17 H17 N2 O11 Re S2
Mr	675.66	675.64
Dx,g cm-3	2.002	2.002
Z	2	2
Mu (mm-1)	5.668	5.668
F000	656.0	656.0
F000'	654.84	
h,k,lmax	10,15,16	10,15,16
Nref	5147	5144
Tmin,Tmax	0.199,0.567	0.345,1.000
Tmin'	0.126	

Correction method= # Reported T Limits: Tmin=0.345 Tmax=1.000
AbsCorr = MULTI-SCAN

Data completeness= 0.999 Theta(max)= 27.482

R(reflections)= 0.0283(4734) wR2(reflections)= 0.0710(5144)

S = 1.069 Npar= 303

The following ALERTS were generated. Each ALERT has the format
test-name_ALERT_alert-type_alert-level.
Click on the hyperlinks for more details of the test.

Alert level C

```
PLAT971_ALERT_2_C Check Calcd Resid. Dens.  0.82Ang From Re1            2.06 eA-3
PLAT971_ALERT_2_C Check Calcd Resid. Dens.  0.86Ang From Re1            1.77 eA-3
PLAT971_ALERT_2_C Check Calcd Resid. Dens.  0.91Ang From C1A            1.73 eA-3
PLAT971_ALERT_2_C Check Calcd Resid. Dens.  1.87Ang From C8             1.70 eA-3
PLAT973_ALERT_2_C Check Calcd Positive Resid. Density on       Re1      1.14 eA-3
PLAT975_ALERT_2_C Check Calcd Resid. Dens.  0.96Ang From O1W     .      0.66 eA-3
PLAT975_ALERT_2_C Check Calcd Resid. Dens.  0.77Ang From O1W     .      0.64 eA-3
```

Alert level G

```
PLAT007_ALERT_5_G Number of Unrefined Donor-H Atoms .............          2 Report
              H1WA  H1WB
PLAT154_ALERT_1_G The s.u.'s on the Cell Angles are Equal ..(Note)      0.002 Degree
PLAT432_ALERT_2_G Short Inter X...Y Contact  O2B      ..C15      .       2.94 Ang.
                                           -1+x,y,-1+z  =      1_454 Check
PLAT432_ALERT_2_G Short Inter X...Y Contact  O2C      ..C19      .       2.98 Ang.
                                            -x,2-y,2-z  =      2_577 Check
PLAT720_ALERT_4_G Number of Unusual/Non-Standard Labels .........         10 Note
              S2MS    H1WA    H1WB    O4MS    O3MS    O5MS    C1MS    H1MA
              H1MB    H1MC
PLAT910_ALERT_3_G Missing # of FCF Reflection(s) Below Theta(Min).         3 Note
                0  1  0,   0 -1  1,   0  0  1,
PLAT969_ALERT_5_G The 'Henn et al.' R-Factor-gap value ..........      1.851 Note
              Predicted wR2: Based on SigI**2  3.83 or SHELX Weight  6.64
PLAT978_ALERT_2_G Number C-C Bonds with Positive Residual Density.         0 Info
```

---

0 **ALERT level A** = Most likely a serious problem - resolve or explain
0 **ALERT level B** = A potentially serious problem, consider carefully
7 **ALERT level C** = Check. Ensure it is not caused by an omission or oversight
8 **ALERT level G** = General information/check it is not something unexpected

1 ALERT type 1 CIF construction/syntax error, inconsistent or missing data
10 ALERT type 2 Indicator that the structure model may be wrong or deficient
1 ALERT type 3 Indicator that the structure quality may be low
1 ALERT type 4 Improvement, methodology, query or suggestion
2 ALERT type 5 Informative message, check

Datablock: moc331

Bond precision: C-C = 0.0081 A Wavelength=0.71073

Cell:	a=9.4366(3)	b=11.7454(4)	c=11.8245(4)
	alpha=65.301(3)	beta=83.502(3)	gamma=89.778(3)
Temperature:	150 K		

	Calculated	Reported
Volume	1181.67(7)	1181.67(7)
Space group	P -1	P -1
Hall group	-P 1	-P 1
Moiety formula	C16 H14 N2 O8 Re S, C F3 O3 S	C16 H14 N2 O8 Re S, C F3 O3 S
Sum formula	C17 H14 F3 N2 O11 Re S2	C17 H14 F3 N2 O11 Re S2
Mr	729.63	729.62
Dx,g cm-3	2.051	2.051
Z	2	2
Mu (mm-1)	5.402	5.402
F000	704.0	704.0
F000’	702.93	
h,k,lmax	12,15,15	12,15,15
Nref	5434	5360
Tmin,Tmax	0.284,0.850	0.354,1.000
Tmin’	0.111	

Correction method= # Reported T Limits: Tmin=0.354 Tmax=1.000
AbsCorr = MULTI-SCAN

Data completeness= 0.986 Theta(max)= 27.484

R(reflections)= 0.0359(4809) wR2(reflections)= 0.0876(5360)

S = 1.035 Npar= 333

The following ALERTS were generated. Each ALERT has the format
test-name_ALERT_alert-type_alert-level.
Click on the hyperlinks for more details of the test.

Alert level C

```
PLAT244_ALERT_4_C Low    'Solvent' Ueq as Compared to Neighbors of      S1TF Check
PLAT342_ALERT_3_C Low Bond Precision on  C-C Bonds ...............    0.00811 Ang.
PLAT971_ALERT_2_C Check Calcd Resid. Dens.  0.91Ang From Re1             1.73 eA-3
PLAT971_ALERT_2_C Check Calcd Resid. Dens.  0.94Ang From Re1             1.66 eA-3
PLAT971_ALERT_2_C Check Calcd Resid. Dens.  0.95Ang From Re1             1.52 eA-3
PLAT972_ALERT_2_C Check Calcd Resid. Dens.  0.71Ang From Re1            -1.51 eA-3
PLAT973_ALERT_2_C Check Calcd Positive Resid. Density on       Re1       1.42 eA-3
PLAT976_ALERT_2_C Check Calcd Resid. Dens.  1.00Ang From O2TF    .      -0.87 eA-3
```

Alert level G

```
PLAT002_ALERT_2_G Number of Distance or Angle Restraints on AtSite          2 Note
PLAT007_ALERT_5_G Number of Unrefined Donor-H Atoms .............           1 Report
              H1WA
PLAT154_ALERT_1_G The s.u.'s on the Cell Angles are Equal ..(Note)      0.003 Degree
PLAT172_ALERT_4_G The CIF-Embedded .res File Contains DFIX Records          1 Report
PLAT244_ALERT_4_G Low    'Solvent' Ueq as Compared to Neighbors of       C5TF Check
PLAT380_ALERT_4_G Incorrectly? Oriented X(sp2)-Methyl Moiety .....        C20 Check
PLAT434_ALERT_2_G Short Inter HL..HL Contact F7TF     ..F7TF     .       2.80 Ang.
                                                 -x,1-y,2-z  =      2_567 Check
PLAT720_ALERT_4_G Number of Unusual/Non-Standard Labels ..........         10 Note
              S1TF    H1WA    F6TF    O3TF    O4TF    F7TF    O2TF    F8TF
              C5TF    H1WB
PLAT860_ALERT_3_G Number of Least-Squares Restraints ............           1 Note
PLAT910_ALERT_3_G Missing # of FCF Reflection(s) Below Theta(Min).          4 Note
                1  0  0,   0  1  0,   0  0  1,   0  1  1,
PLAT912_ALERT_4_G Missing # of FCF Reflections Above STh/L=  0.600         70 Note
PLAT941_ALERT_3_G Average HKL Measurement Multiplicity ..........         4.8 Low
PLAT969_ALERT_5_G The 'Henn et al.' R-Factor-gap value ..........       1.941 Note
              Predicted wR2: Based on SigI**2  4.51 or SHELX Weight  8.46
PLAT978_ALERT_2_G Number C-C Bonds with Positive Residual Density.          0 Info
```

```
   0 ALERT level A = Most likely a serious problem - resolve or explain
   0 ALERT level B = A potentially serious problem, consider carefully
   8 ALERT level C = Check. Ensure it is not caused by an omission or oversight
  14 ALERT level G = General information/check it is not something unexpected

   1 ALERT type 1 CIF construction/syntax error, inconsistent or missing data
   9 ALERT type 2 Indicator that the structure model may be wrong or deficient
   4 ALERT type 3 Indicator that the structure quality may be low
   6 ALERT type 4 Improvement, methodology, query or suggestion
   2 ALERT type 5 Informative message, check
```

It is advisable to attempt to resolve as many as possible of the alerts in all categories. Often the minor alerts point to easily fixed oversights, errors and omissions in your CIF or refinement strategy, so attention to these fine details can be worthwhile. In order to resolve some of the more serious problems it may be necessary to carry out additional measurements or structure refinements. However, the purpose of your study may justify the reported deviations and the more serious of these should normally be commented upon in the discussion or experimental section of a paper or in the "special_details" fields of the CIF. checkCIF was carefully designed to identify outliers and unusual parameters, but every test has its limitations and alerts that are not important in a particular case may appear. Conversely, the absence of alerts does not guarantee there are no aspects of the results needing attention. It is up to the individual to critically assess their own results and, if necessary, seek expert advice.

Publication of your CIF in IUCr journals

A basic structural check has been run on your CIF. These basic checks will be run on all CIFs submitted for publication in IUCr journals (*Acta Crystallographica*, *Journal of Applied Crystallography*, *Journal of Synchrotron Radiation*); however, if you intend to submit to *Acta Crystallographica Section C* or *E* or *IUCrData*, you should make sure that full publication checks are run on the final version of your CIF prior to submission.

Publication of your CIF in other journals

Please refer to the *Notes for Authors* of the relevant journal for any special instructions relating to CIF submission.

PLATON version of 22/08/2024; check.def file version of 21/08/2024

Datablock mod25_auto - ellipsoid plot

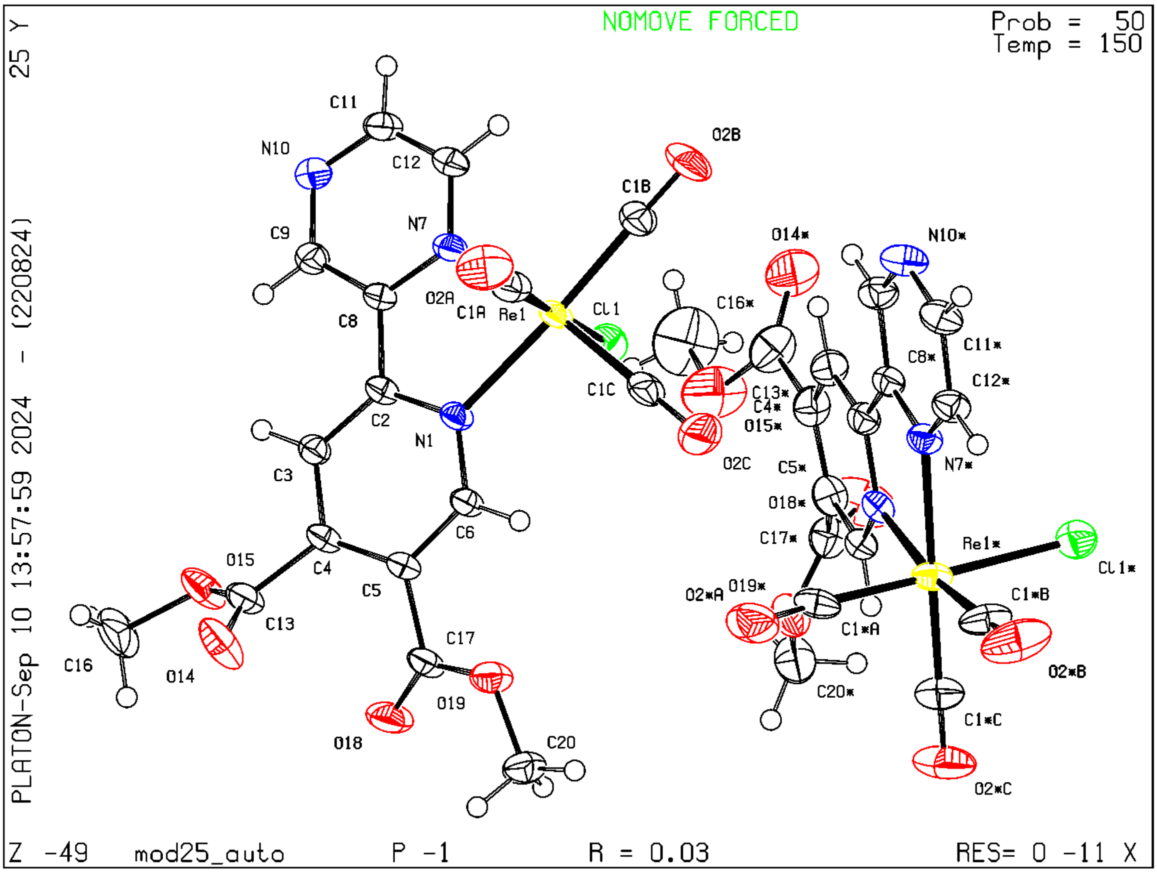

Datablock mod12_auto - ellipsoid plot

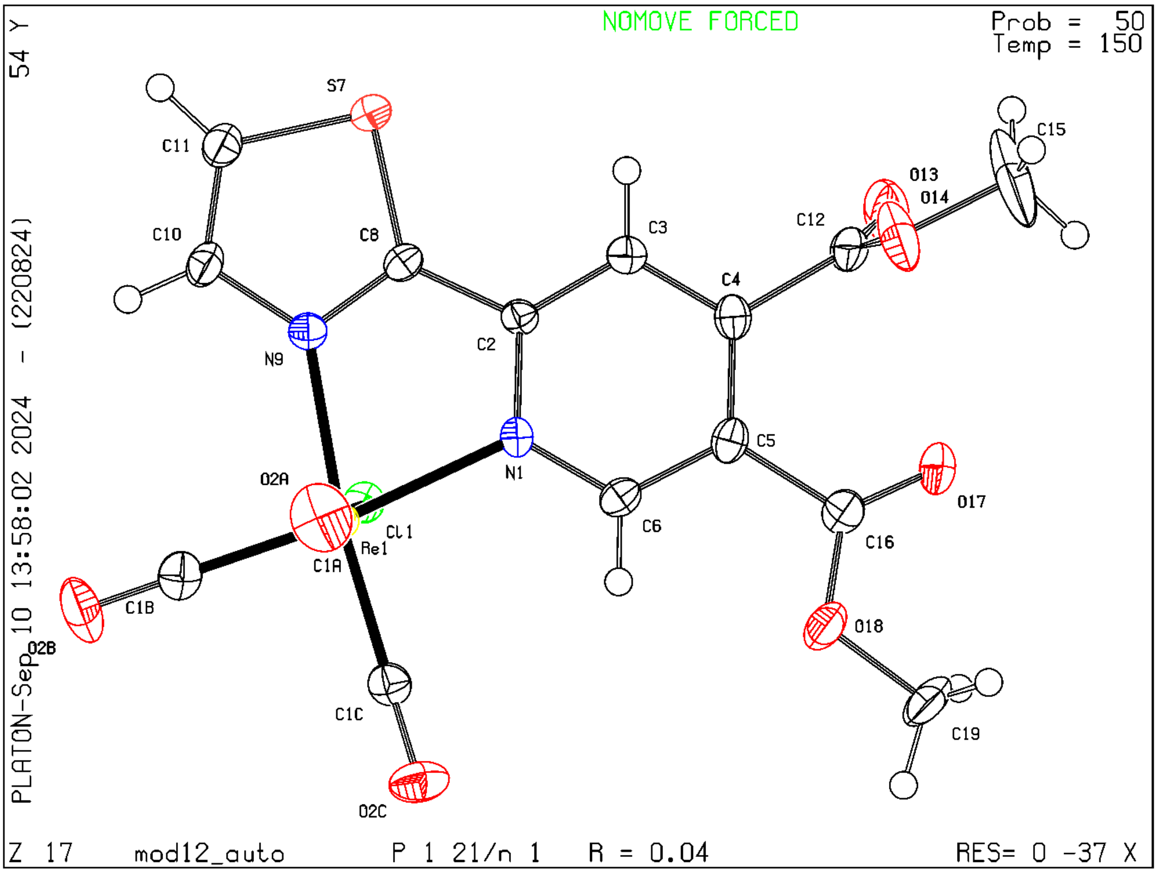

Datablock mod28_auto - ellipsoid plot

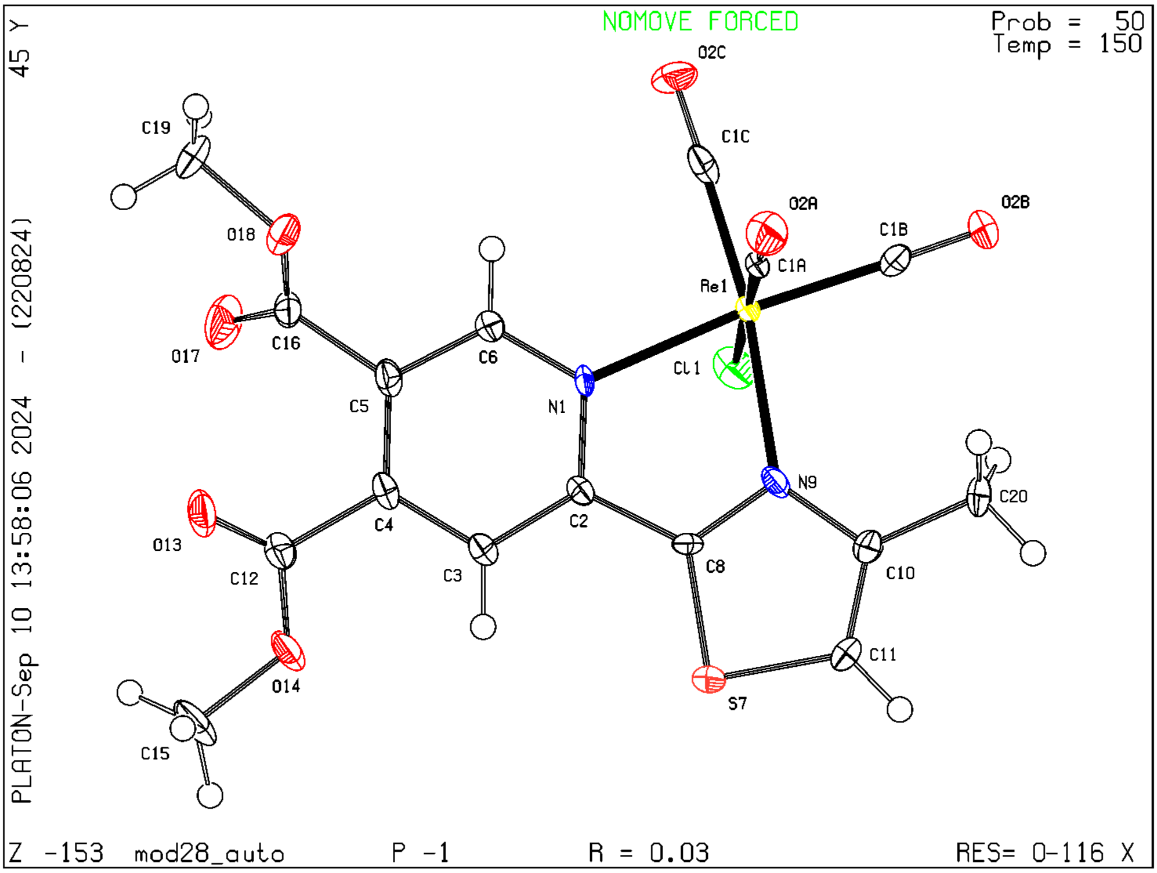

Datablock moc177 - ellipsoid plot

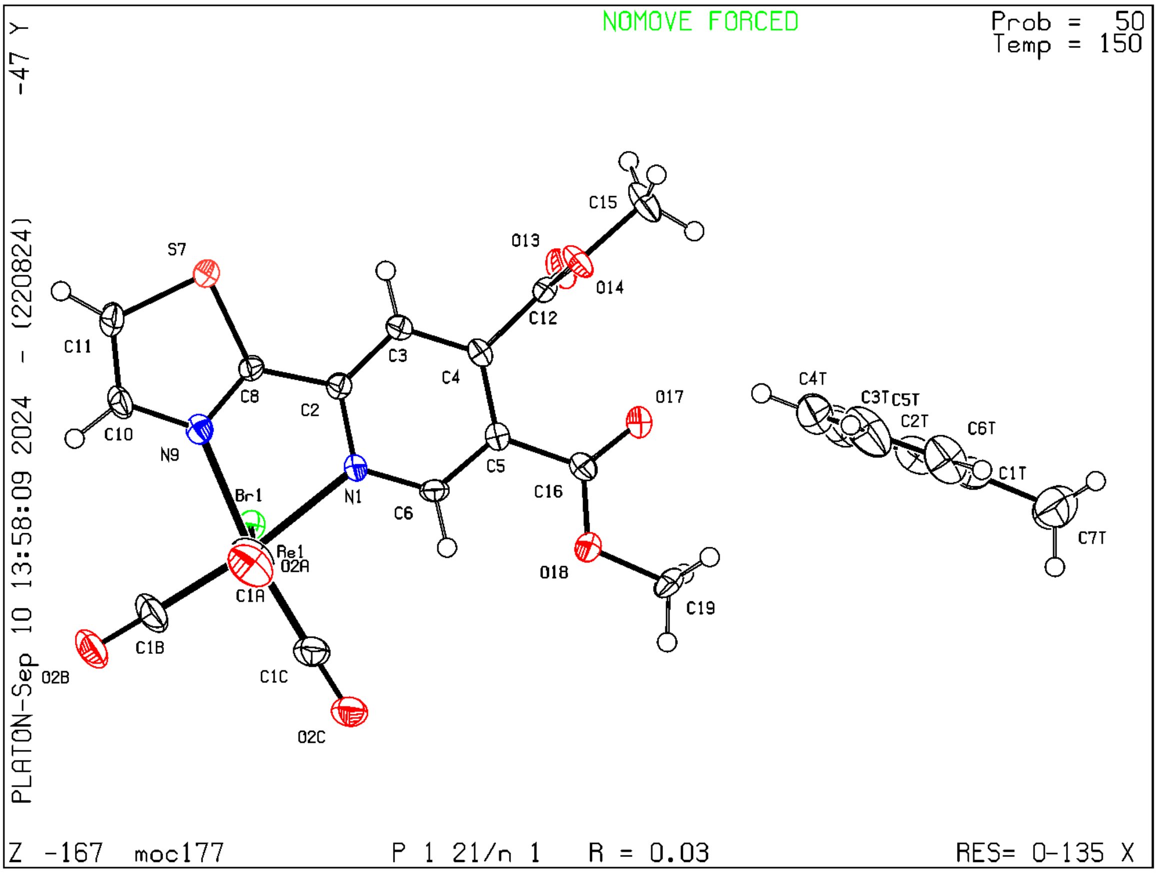

Datablock moc179 - ellipsoid plot

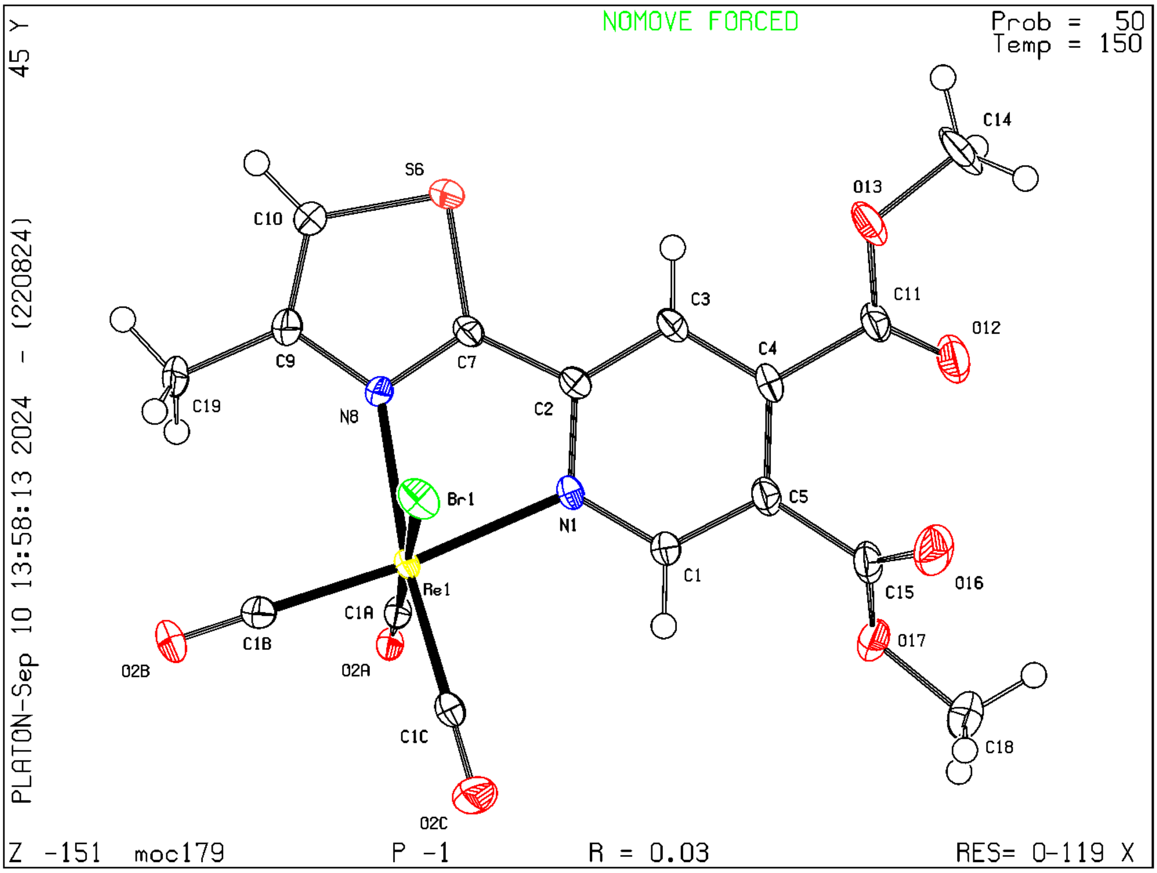

Datablock mod26_auto - ellipsoid plot

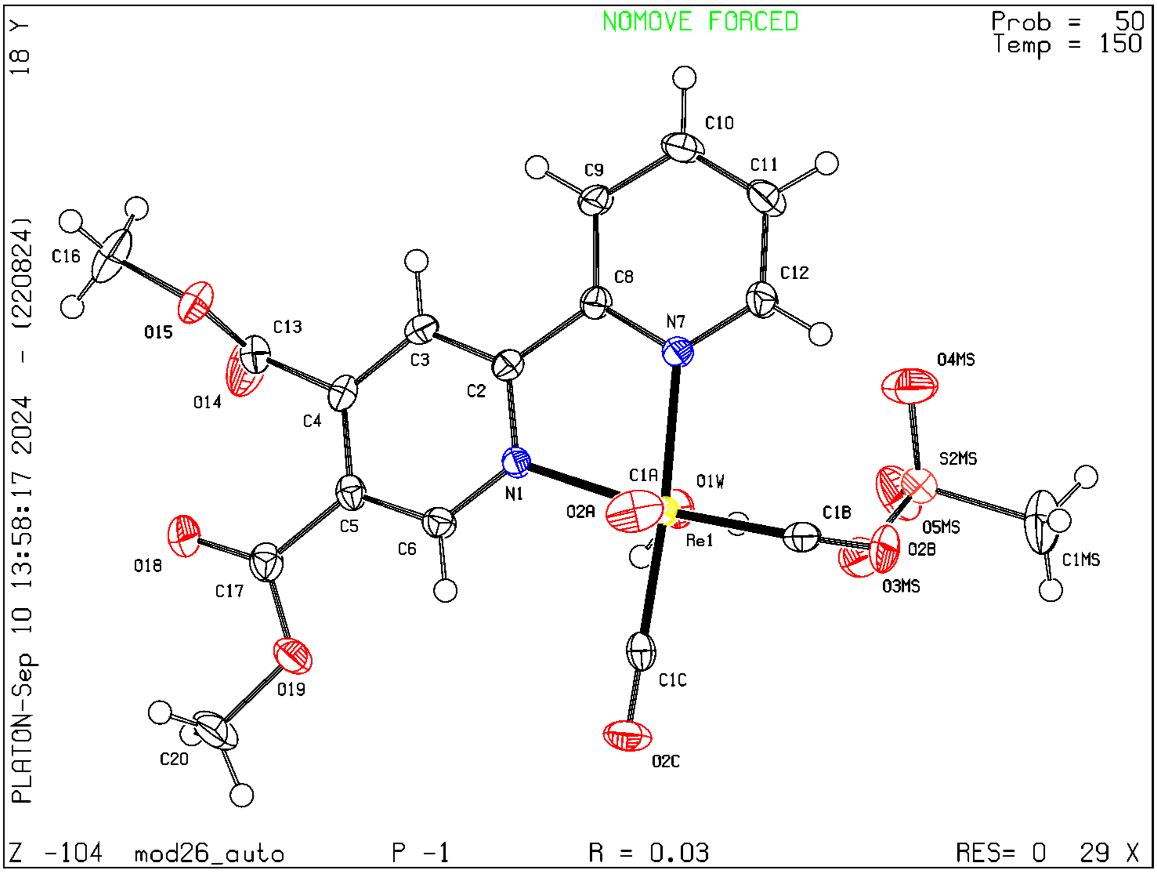

Datablock mod27_auto - ellipsoid plot

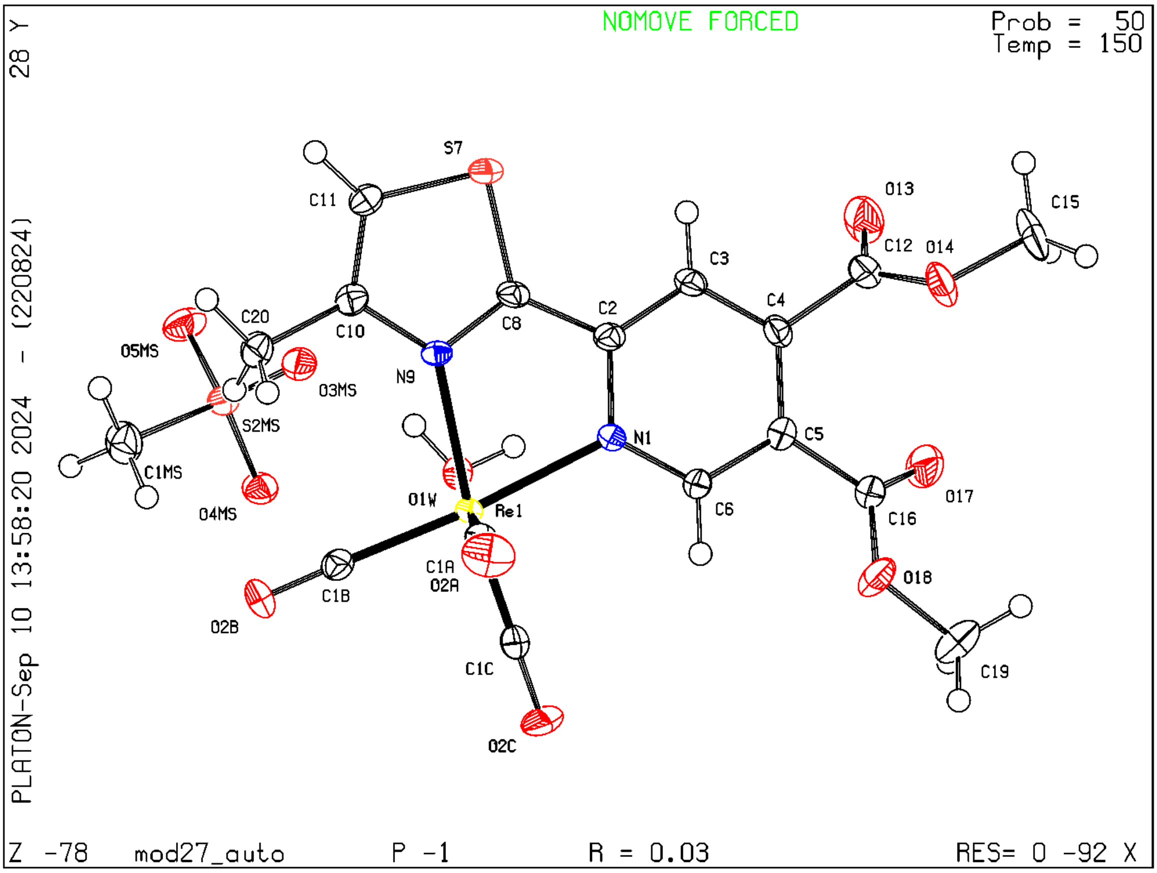

Datablock moc331 - ellipsoid plot

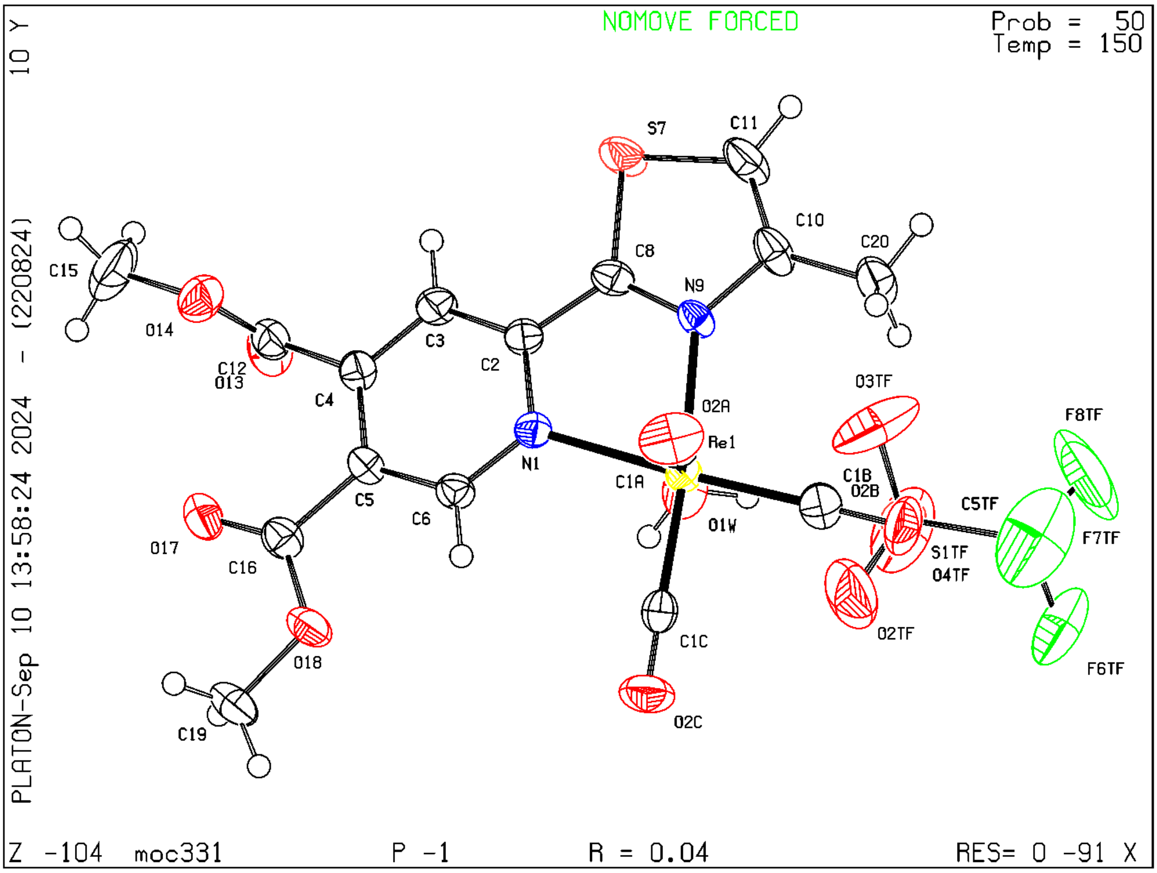

Supplement: Supplementary file 2 — ao4c07117_si_002.zip [file ao4c07117_si_002.zip › X-ray data/Crystal structures Re+pdca checkcif report sumbit.pdf]
